# Solvable Model of a Generic Driven Mixture of Trapped Bose–Einstein Condensates and Properties of a Many-Boson Floquet State at the Limit of an Infinite Number of Particles

**DOI:** 10.3390/e22121342

**Published:** 2020-11-26

**Authors:** Ofir E. Alon

**Affiliations:** 1 Department of Mathematics, University of Haifa, Haifa 3498838, Israel; ofir@research.haifa.ac.il; 2 Haifa Research Center for Theoretical Physics and Astrophysics, University of Haifa, Haifa 3498838, Israel

**Keywords:** solvable models, time-dependent Schrödinger equation, driven Bose–Einstein dondensates, mixtures, Floquet Hamiltonian, time-dependent reduced density matrices, coupled nonlinear Schrödinger equations, infinite-particle-number limit, angular momentum, harmo+nic-interaction models

## Abstract

A solvable model of a periodically driven trapped mixture of Bose–Einstein condensates, consisting of $N_{1}$ interacting bosons of mass $m_{1}$ driven by a force of amplitude $f_{L,1}$ and $N_{2}$ interacting bosons of mass $m_{2}$ driven by a force of amplitude $f_{L,2}$, is presented. The model generalizes the harmonic-interaction model for mixtures to the time-dependent domain. The resulting many-particle ground Floquet wavefunction and quasienergy, as well as the time-dependent densities and reduced density matrices, are prescribed explicitly and analyzed at the many-body and mean-field levels of theory for finite systems and at the limit of an infinite number of particles. We prove that the time-dependent densities per particle are given at the limit of an infinite number of particles by their respective mean-field quantities, and that the time-dependent reduced one-particle and two-particle density matrices per particle of the driven mixture are 100% condensed. Interestingly, the quasienergy per particle does not coincide with the mean-field value at this limit, unless the relative center-of-mass coordinate of the two Bose–Einstein condensates is not activated by the driving forces $f_{L,1}$ and $f_{L,2}$. As an application, we investigate the imprinting of angular momentum and its fluctuations when steering a Bose–Einstein condensate by an interacting bosonic impurity and the resulting modes of rotations. Whereas the expectation values per particle of the angular-momentum operator for the many-body and mean-field solutions coincide at the limit of an infinite number of particles, the respective fluctuations can differ substantially. The results are analyzed in terms of the transformation properties of the angular-momentum operator under translations and boosts, and as a function of the interactions between the particles. Implications are briefly discussed.

## 1. Introduction

Quantum dynamics is central to nuclear, atomic, molecular, and optical physics [1,2,3,4,5,6,7,8]. In order to theoretically describe the dynamics of a quantum many-particle system, it is generally required to solve and analyze the time-dependent Schrödinger equation. Usually, a numerical treatment of a faithful representation of the time-dependent system under investigation is the only available tool, whereas the existence of solvable time-dependent many-particle models is scarcer.

A particularly exciting arena for quantum dynamics across many disciplines is that in which the system’s Hamiltonian is periodic in time [9,10,11,12,13,14,15,16,17,18,19,20,21,22,23,24,25,26,27,28,29,30,31,32,33,34,35,36,37,38,39,40,41,42,43,44,45,46,47,48,49,50,51,52,53,54,55,56,57,58]. The time-periodic Hamiltonian defines the Floquet Hamiltonian and governs the Floquet eigenvalue equation in an extended Hilbert space [11,12], in analogy to the static Hamiltonian, which governs the Schrödinger eigenvalue equation in the standard Hilbert space of a time-independent system.

In the field of Bose–Einstein condensates [4,6], the relation between the many-body and mean-field treatments of bosonic systems at the limit of an infinite number of particles has drawn much attention [59,60,61,62,63,64,65,66,67,68,69,70,71,72,73,74]. At the infinite-particle-number limit, the interaction parameter (i.e., the product of the number of bosons times the interaction strength) is held fixed while the number of particles is increased to infinity. Under well-defined conditions, it has been proved for the ground state of trapped bosons that the many-body reduced one-particle and two-particle density matrices [75,76] per particle are 100% condensed and coincide with the respective Gross–Pitaevskii results [60,61]. Similarly, the many-body energy per particle as well as the one-particle and two-particle densities per particle coincide in this limit with the Gross–Pitaevskii results. Analogous results exist for the dynamics of interacting bosons with time-independent Hamiltonians at a definite propagation time [62,63]. Generalizations for trapped mixtures of two kinds of identical bosons cover the many-body energy per particle, intraspecies and interspecies densities per particle, and reduced density matrices per particle, and show coincidence in the limit of an infinite number of particles with the Gross–Pitaevskii results, 100% condensation of each of the species, and separability of interspecies quantities in the mixture [67,68,69].

However, differences between many-body and mean-field results of fully condensed bosonic systems can emerge in other quantities, in particular, variances of operators, such as the position, momentum, and angular-momentum operators [64,65,72,74], and stem from the differences between the many-body and mean-field wavefunctions themselves [66,70]. The differences in the variances of observables are instrumental in defining and quantifying correlations at the infinite-particle-number limit; for instance, distinguishing between 100% condensed trapped bosons, either in the ground state or undergoing dynamics in two spatial dimensions, in which the anisotropy along the *x* and *y* directions of their position variance per particle and density per particle are alike, and 100% condensed bosons, in which this anisotropy is opposite [71,73].

In this work, we present a solvable model of a periodically driven trapped mixture of Bose–Einstein condensates. Mixtures of Bose–Einstein condensates have been a vivid topic [77,78,79,80,81,82,83,84,85,86,87,88,89,90,91,92,93,94,95,96,97,98,99,100,101,102,103,104]. Here, we introduce the driven harmonic-interaction model for mixtures, that is, $N_{1}$ bosons of mass $m_{1}$ interacting by harmonic interparticle interaction of strength $\lambda_{1}$ and driven by a force of amplitude $f_{L,1}$, and $N_{2}$ bosons of mass $m_{2}$ interacting by harmonic interparticle interaction of strength $\lambda_{2}$ and driven by a force of amplitude $f_{L,1}$. All bosons are trapped in a harmonic potential of frequency ω and, furthermore, bosons of species 1 and species 2 interact with each other by harmonic interparticle interaction of strength $\lambda_{12}$. The model generalizes the harmonic-interaction model for mixtures to the time-dependent domain, and builds on a long-standing interest in harmonic-interaction models [69,105,106,107,108,109,110,111,112,113,114,115,116,117,118,119,120,121].

Besides solving the model and discussing properties, our main purpose is to investigate the infinite particle limit of a Floquet many-boson state, namely, the connection between the many-body and mean-field time-dependent reduced density matrices, time-dependent densities, quasienergies, and, eventually, variances. In this way, we expand on studies from the literature on the ground state and dynamics with time-independent Hamiltonians of bosonic systems to the realm of Floquet bosonic systems. Both the many-body and mean-field solutions of the trapped driven mixture are required and derived in closed form, making the proofs of properties explicit. As an application, the expectation value and variance of the many-boson angular momentum operator when a Bose–Einstein condensate is steered by interacting bosons consisting of a different species are explored fully analytically within our model. The modes of rotations are investigated as a function of the steering frequency and interspecies interaction, and the differences between the many-body and mean-field variances are explored.

The structure of the paper is as follows. In Section 2, we consider the driven single-species harmonic-interaction model, solve it at the many-body and mean-field levels, and derive working tools. In Section 3, we present the driven harmonic-interaction model for mixtures, solve it at the many-body and mean-field levels of theory, discuss its properties, and analyze the limit of an infinite number of particles. In Section 4, we analyze the imprinting of angular momentum when one of the species is steered and embedded in a second species that is not steered, and discuss the many-body and mean-field angular-momentum variances at the infinite-particle-number limit. In Section 5, a summary and outlook are given. Finally, Appendix A, Appendix B and Appendix C collect supplemental results, details of derivations, and further tools.

## 2. The Driven Harmonic-Interaction Model

Consider the driven classical harmonic oscillator in one spatial dimension. The classical equation of motion and its particular solution are given by

(1) $$\frac{d^{2}x\left(t\right)}{dt^{2}}+\omega^{2}x\left(t\right)=\frac{f_{L}\cos\left(\omega_{L}t\right)}{m},\;x\left(t\right)=a\cos\left(\omega_{L}t\right),\;a=\frac{f_{L}}{m(\omega^{2}-\omega_{L}^{2})}.$$

Here and hereafter, the amplitude of the driving force satisfies $f_{L}\neq 0$, the driving frequency $\omega_{L}>0$, and the trapping frequency w>0; for convenience, the off-resonance condition $\omega_{L}\neq \omega$ holds. For $\omega_{L}<\omega$, the amplitude of oscillations *a* is in phase with $f_{L}$, whereas for $\omega_{L}>\omega$ and the high-frequency limit, *a* has the opposite phase to $f_{L}$. The particular solution x(t) appears in the driven quantum harmonic oscillator problem and in its many-boson generalization, the driven harmonic-interaction model.

The driven quantum harmonic oscillator [122,123] obeys the time-dependent Schrödinger equation

(2) $$\begin{array}{ccc} & & \hat{h}\left(x,t\right)\psi \left(x,t\right)=i\frac{\partial \psi (x,t)}{\partial t},\;\hat{h}\left(x,t\right)=-\frac{1}{2m}\frac{\partial^{2}}{\partial x^{2}}+\frac{1}{2}m\omega^{2}x^{2}-xf_{L}\cos\left(\omega_{L}t\right)=\\ & & =-\frac{1}{2m}\frac{\partial^{2}}{\partial x^{2}}+\frac{1}{2}m\omega^{2}{\left[x-\frac{f_{L}}{m(\omega^{2}-\omega_{L}^{2})}\cos\left(\omega_{L}t\right)\right]}^{2}-\frac{f_{L}^{2}\omega^{2}}{2m{\left(\omega^{2}-\omega_{L}^{2}\right)}^{2}}\cos^{2}\left(\omega_{L}t\right)+\\ & & +\frac{\omega_{L}^{2}}{\omega^{2}-\omega_{L}^{2}}xf_{L}\cos\left(\omega_{L}t\right)=-\frac{1}{2m}\frac{\partial^{2}}{\partial x^{2}}+\frac{1}{2}m\omega^{2}{\left[x-x(t)\right]}^{2}-\frac{1}{2}m\omega^{2}x^{2}\left(t\right)+m\omega_{L}^{2}x\left(t\right)x,\; \end{array}$$

where, throughout this work, ℏ=1. It has a set of Floquet solutions corresponding to the eigenfunctions of the non-driven system. The solution corresponding to the ground state of the harmonic oscillator, hereafter termed, for brevity, the ground Floquet solution, is given by

(3) $$\begin{array}{ccc} & & \psi \left(x,t\right)={\left(\frac{m\omega }{\pi }\right)}^{\frac{1}{4}}e^{-i\varepsilon (t)}e^{-\frac{m\omega }{2}{\left[x-\frac{f_{L}}{m(\omega^{2}-\omega_{L}^{2})}\cos\left(\omega_{L}t\right)\right]}^{2}}e^{-i\frac{f_{L}\omega_{L}}{\left(\omega^{2}-\omega_{L}^{2}\right)}\sin\left(\omega_{L}t\right)x}=\\ & & ={\left(\frac{m\omega }{\pi }\right)}^{\frac{1}{4}}e^{-i\varepsilon (t)}e^{-\frac{m\omega }{2}{\left[x-x(t)\right]}^{2}}e^{+im\dot{x}\left(t\right)x},\; \end{array}$$

where

(4) $$\begin{array}{ccc} & & \varepsilon \left(t\right)=\frac{\omega }{2}t+\int^{t}dt^{\prime }\left[\frac{1}{2}m{\dot{x}}^{2}\left(t\right)-\frac{1}{2}m\omega^{2}x^{2}\left(t\right)\right]=\frac{\omega }{2}t+\int^{t}dt^{\prime }[\frac{f_{L}^{2}\omega_{L}^{2}}{2m{\left(\omega^{2}-\omega_{L}^{2}\right)}^{2}}\sin^{2}\left(\omega_{L}t^{\prime }\right)-\\ & & -\frac{f_{L}^{2}\omega^{2}}{2m{\left(\omega^{2}-\omega_{L}^{2}\right)}^{2}}\cos^{2}\left(\omega_{L}t^{\prime }\right)]=\varepsilon_{F}t-\frac{f_{L}^{2}\left(\omega^{2}+\omega_{L}^{2}\right)}{8m\omega_{L}{\left(\omega^{2}-\omega_{L}^{2}\right)}^{2}}\sin\left(2\omega_{L}t\right)\; \end{array}$$

is the time-dependent phase and [123]

(5) $$\varepsilon_{F}=\frac{\omega }{2}-\frac{f_{L}^{2}}{4m(\omega^{2}-\omega_{L}^{2})}$$

is an eigenvalue of the Floquet Hamiltonian, $\left[\hat{h}\left(x,t\right)-i\frac{\partial }{\partial t}\right]\bar{\psi }\left(x,t\right)=\varepsilon_{F}\bar{\psi }\left(x,t\right)$, and called quasienergy. The Floquet quasienergy state $\bar{\psi }\left(x,t\right)$ is the periodic part of $\psi \left(x,t\right)=e^{-i\varepsilon_{F}t}\bar{\psi }\left(x,t\right)$. There are three contributions to the time-dependent phase (4) and, hence, to the quasienergy (5): The first is the energy of the stationary problem, the second originates, upon acting with the kinetic-energy operator, from the spatial-dependent phase of the time-dependent wavefunction, and the third results from completing the square in the time-dependent Hamiltonian. The latter’s contribution and its renormalization by interaction in particular would play an interesting role in the driven many-boson problem. In the present work, we study interacting many-boson generalizations of the driven harmonic oscillator—explicitly, time-dependent harmonic-interaction models—and analyze their ground Floquet solutions at the many-body and mean-field levels of theory.

Consider now the single-species harmonic-interaction model [108] plus a driving term,

(6) $$\hat{H}\left(x_{1},\ldots ,x_{N},t\right)=\sum_{j=1}^{N}\left[-\frac{1}{2m}\frac{\partial^{2}}{\partial x_{j}^{2}}+\frac{1}{2}m\omega^{2}x_{j}^{2}-x_{j}f_{L}\cos\left(\omega_{L}t\right)\right]+\lambda \sum_{1\leq j<k}^{N}{\left(x_{j}-x_{k}\right)}^{2},$$

describing *N* harmonically interacting trapped bosons driven by a time-periodic force of amplitude $f_{L}$. Using the Jacobi coordinates

(7) $$Q_{k}=\frac{1}{\sqrt{k(k+1)}}\sum_{j=1}^{k}\left(x_{k+1}-x_{j}\right),\;1\leq k\leq N-1,\;Q_{N}=\frac{1}{\sqrt{N}}\sum_{j=1}^{N}x_{j}$$

the time-dependent Hamiltonian is readily diagonalized,

(8) $$\begin{array}{ccc} & & \hat{H}\left(Q_{1},\ldots ,Q_{N},t\right)=\\ & & =\sum_{j=1}^{N-1}\left(-\frac{1}{2m}\frac{\partial^{2}}{\partial Q_{j}^{2}}+\frac{1}{2}m\Omega^{2}Q_{j}^{2}\right)+\left[-\frac{1}{2m}\frac{\partial^{2}}{\partial Q_{N}^{2}}+\frac{1}{2}m\omega^{2}Q_{N}^{2}-Q_{N}\sqrt{N}f_{L}\cos\left(\omega_{L}t\right)\right],\; \end{array}$$

and describes a single driven center-of-mass oscillator and N−1 time-independent relative coordinates’ degrees of freedom. The frequency $\Omega =\sqrt{\omega^{2}+\frac{2\lambda N}{m}}$ of the relative coordinates is dressed by the interparticle interaction. The (driven) system is bound provided $\lambda >-\frac{m\omega^{2}}{2N}$, i.e., the interaction is either attractive (λ>0) or not too repulsive (λ<0).

The normalized ground Floquet solution of the time-dependent many-boson Schrödinger equation $\hat{H}\left(x_{1},\ldots ,x_{N},t\right)\Psi \left(x_{1},\ldots ,x_{N},t\right)=i\frac{\Psi (x_{1},\ldots ,x_{N},t)}{\partial t}$ is given in terms of the Jacobi coordinates by

(9) $$\begin{array}{ccc} & & \Psi \left(Q_{1},\ldots ,Q_{N},t\right)={\left(\frac{m\Omega }{\pi }\right)}^{\frac{N-1}{4}}{\left(\frac{m\omega }{\pi }\right)}^{\frac{1}{4}}e^{-iE(t)}e^{-\frac{1}{2}m\left\{\Omega \sum_{j=1}^{N-1}Q_{j}^{2}+\omega {\left[Q_{N}-Q_{N}\left(t\right)\right]}^{2}\right\}}e^{+im{\dot{Q}}_{N}\left(t\right)Q_{N}},\\ & & \;Q_{N}\left(t\right)=\frac{\sqrt{N}f_{L}}{m(\omega^{2}-\omega_{L}^{2})}\cos\left(\omega t\right)=\sqrt{N}x\left(t\right),\; \end{array}$$

where

(10) $$\begin{array}{ccc} & & E\left(t\right)=\frac{(N-1)\Omega +\omega }{2}t+\int^{t}dt^{\prime }\left[\frac{1}{2}m{\dot{Q}}_{N}^{2}\left(t\right)-\frac{1}{2}m\omega^{2}Q_{N}^{2}\left(t\right)\right]=\\ & & =\frac{(N-1)\Omega +\omega }{2}t+\int^{t}dt^{\prime }\left[\frac{Nf_{L}^{2}\omega_{L}^{2}}{2m{\left(\omega^{2}-\omega_{L}^{2}\right)}^{2}}\sin^{2}\left(\omega_{L}t^{\prime }\right)-\frac{Nf_{L}^{2}\omega^{2}}{2m{\left(\omega^{2}-\omega_{L}^{2}\right)}^{2}}\cos^{2}\left(\omega_{L}t^{\prime }\right)\right]=\\ & & =E_{F}t-\frac{Nf_{L}^{2}\left(\omega^{2}+\omega_{L}^{2}\right)}{8m\omega_{L}{\left(\omega^{2}-\omega_{L}^{2}\right)}^{2}}\sin\left(2\omega_{L}t\right)\; \end{array}$$

is the time-dependent phase, and

(11) $$E_{F}=\frac{(N-1)\Omega +\omega }{2}-\frac{Nf_{L}^{2}}{4m(\omega^{2}-\omega_{L}^{2})}$$

is the many-boson quasienergy. Comparison of the many-boson (11) and single-particle (5) quasienergies shows that they have a similar structure. The later emanates from the fact that for both systems the driving force is coupled to the center of mass only, the frequency of which being the trapping frequency ω. The many-boson quasienergy state $\bar{\Psi }\left(Q_{1},\ldots ,Q_{N},t\right)$ is an eigenfunction of the many-body Floquet Hamiltonian,

(12) $$\left[\hat{H}\left(Q_{1},\ldots ,Q_{N},t\right)-i\frac{\partial }{\partial t}\right]\bar{\Psi }\left(Q_{1},\ldots ,Q_{N},t\right)=E_{F}\bar{\Psi }\left(Q_{1},\ldots ,Q_{N},t\right),$$

where $\Psi \left(Q_{1},\ldots ,Q_{N},t\right)=e^{-iE_{F}t}\bar{\Psi }\left(Q_{1},\ldots ,Q_{N},t\right)$.

To proceed, we transform from the Jacobi coordinates, for which the wavefunction is separable, to the laboratory frame, and get

(13) $$\begin{array}{ccc} & & \Psi \left(x_{1},\ldots ,x_{N},t\right)={\left(\frac{m\Omega }{\pi }\right)}^{\frac{N-1}{4}}{\left(\frac{m\omega }{\pi }\right)}^{\frac{1}{4}}e^{-iE(t)}e^{+imN\dot{x}\left(t\right)x\left(t\right)}\times \\ & & \times e^{-\frac{\alpha }{2}\sum_{j=1}^{N}{\left[x_{j}-x\left(t\right)\right]}^{2}-\beta \sum_{1\leq j<k}^{N}\left[x_{j}-x\left(t\right)\right]\left[x_{k}-x\left(t\right)\right]}e^{+im\dot{x}\left(t\right)\sum_{j=1}^{N}\left[x_{j}-x\left(t\right)\right]},\\ & & \alpha =m\Omega +\beta ,\;\beta =m\frac{\omega -\Omega }{N}.\; \end{array}$$

In obtaining the wavefunction in the laboratory frame, we made use of the relations that $Q_{N}-Q_{N}\left(t\right)=\frac{1}{\sqrt{N}}\sum_{j=1}^{N}\left[x_{j}-x\left(t\right)\right]$ and that the relative Jacobi coordinates are invariant under the translation of all coordinates by x(t).

The determination of the time-dependent reduced density matrices follows in essentially the same way as for the undriven system [108]. Importantly, since the driving is coupled to the center-of-mass coordinate only, all coordinates are translated by the same amplitude, the classical amplitude x(t), and the coherence properties of the driven system, like the eigenvalues of the time-dependent reduced one-particle density matrix, are exactly those of the undriven system; in particular, these eigenvalues are time independent. Explicitly, using the normalized time-dependent wavefunction and from the *N*-particle density matrix,

(14) $$\begin{array}{ccc} & & \Psi \left(x_{1},\ldots ,x_{N},t\right)\Psi^{*}\left(x_{1}^{\prime },\ldots ,x_{N}^{\prime },t\right)={\left(\frac{m\Omega }{\pi }\right)}^{\frac{N-1}{2}}{\left(\frac{m\omega }{\pi }\right)}^{\frac{1}{2}}\times \\ & & \times e^{-\frac{\alpha }{2}\sum_{j=1}^{N}\left\{{\left[x_{j}-x\left(t\right)\right]}^{2}+{\left[x_{j}^{\prime }-x\left(t\right)\right]}^{2}\right\}-\beta \sum_{1\leq j<k}^{N}\left\{\left[x_{j}-x\left(t\right)\right]\left[x_{k}-x\left(t\right)\right]+\left[x_{j}^{\prime }-x\left(t\right)\right]\left[x_{k}^{\prime }-x\left(t\right)\right]\right\}}\times \\ & & \times e^{+im\dot{x}\left(t\right)\sum_{j=1}^{N}\left\{\left[x_{j}-x\left(t\right)\right]-\left[x_{j}^{\prime }-x\left(t\right)\right]\right\}},\; \end{array}$$

the reduced one-particle density matrix $\rho^{\left(1\right)}\left(x,x^{\prime },t\right)=N\int dx_{2}\cdots dx_{N}\Psi \left(x,x_{2},\ldots ,x_{N},t\right)\times$$\Psi^{*}\left(x^{\prime },x_{2},\ldots ,x_{N},t\right)$ is defined. Performing the integrations consecutively over $x_{j},x_{j}^{\prime }=x_{j},j=N,\ldots ,2$, for which the spatial-dependent phase factor falls, and while changing the variable $x_{j}-x\left(t\right)\to x_{j}$, the final result reads

(15) $$\begin{array}{ccc} & & \rho^{\left(1\right)}\left(x,x^{\prime },t\right)=N{\left(\frac{\alpha +C_{1}}{\pi }\right)}^{\frac{1}{2}}e^{-\frac{\alpha }{2}\left\{{\left[x-x(t)\right]}^{2}+{\left[x^{\prime }-x\left(t\right)\right]}^{2}\right\}}e^{-\frac{1}{4}C_{1}{\left\{\left[x-x(t)\right]+\left[x^{\prime }-x\left(t\right)\right]\right\}}^{2}}\times \\ & & \times e^{+im\dot{x}\left(t\right)\left\{\left[x-x(t)\right]-\left[x^{\prime }-x\left(t\right)\right]\right\}},\;C_{1}=-\beta^{2}\frac{N-1}{(\alpha -\beta )+(N-1)\beta }. \end{array}$$

The diagonal of the time-dependent reduced one-particle density matrix is the time-dependent density and is given by

(16) $$\rho^{\left(1\right)}\left(x,t\right)=N{\left(\frac{\alpha +C_{1}}{\pi }\right)}^{\frac{1}{2}}e^{-\left(\alpha +C_{1}\right){\left[x-x(t)\right]}^{2}}=N{\left(\frac{\alpha +C_{1}}{\pi }\right)}^{\frac{1}{2}}e^{-\left(\alpha +C_{1}\right){\left[x-\frac{f_{L}}{m(\omega^{2}-\omega_{L}^{2})}\cos\left(\omega_{L}t\right)\right]}^{2}}.$$

For the respective two-particle quantities, See Appendix A.

So far, we have presented the solution of the driven harmonic-oscillator model at the many-body level of theory. The similar structure of the time-dependent reduced one-particle density matrix per particle of the ground Floquet state (15) and the static reduced one-particle density matrix per particle of the ground state [108] already tells one that in the limit of an infinite number of particles, the former, like the latter, is 100% condensed. To discuss properties of the driven system at the infinite-particle-number limit, we now derive the solution at the mean-field level of theory. This would allow us to explicitly compare the time-dependent reduced density matrices per particle and quasienergies per particle, and prescribe the connection between the many-body and mean-field levels of theory for driven Bose–Einstein condensates, at least within the driven harmonic-interaction model.

Starting from the Gross–Pitaevskii mean-field ansatz for the time-dependent many-boson wavefunction,

(17) $$\Phi \left(x_{1},\ldots ,x_{N},t\right)=\prod_{j=1}^{N}\phi \left(x_{j},t\right),$$

and using the time-dependent variational principle, in either of its forms, the Gross–Pitaevskii mean-field equation for the driven system takes on the form

(18) $$\left[-\frac{1}{2m}\frac{\partial^{2}}{\partial x^{2}}+\frac{1}{2}m\omega^{2}x^{2}-xf_{L}\cos\left(\omega_{L}t\right)+\Lambda \int dx^{\prime }{\left|\phi \left(x^{\prime },t\right)\right|}^{2}{\left(x-x^{\prime }\right)}^{2}\right]\phi \left(x,t\right)=i\frac{\partial \phi (x,t)}{\partial t},$$

where Λ=λ(N−1) is the mean-field interaction parameter. Solving the Gross–Pitaevskii Equation (18) combines the approach used in the treatment of the non-interacting driven oscillator and that of the undriven static mean-field system, and is detailed in Appendix B. The final result can be cast as

(19) $$\begin{array}{ccc} & & \phi \left(x,t\right)={\left(\frac{m\Omega_{GP}}{\pi }\right)}^{\frac{1}{4}}e^{-i\mu (t)}e^{-\frac{m\Omega_{GP}}{2}{\left[x-\frac{f_{L}}{m(\omega^{2}-\omega_{L}^{2})}\cos\left(\omega_{L}t\right)\right]}^{2}}e^{-i\frac{f_{L}\omega_{L}}{\left(\omega^{2}-\omega_{L}^{2}\right)}\sin\left(\omega_{L}t\right)x}=\\ & & ={\left(\frac{m\Omega_{GP}}{\pi }\right)}^{\frac{1}{4}}e^{-i\mu (t)}e^{-\frac{m\Omega_{GP}}{2}{\left[x-x(t)\right]}^{2}}e^{+im\dot{x}\left(t\right)x},\; \end{array}$$

where $\Omega_{GP}=\sqrt{\omega^{2}+\frac{2\Lambda }{m}}$ is the mean-field interaction-dressed frequency and

(20) $$\mu \left(t\right)=\mu_{F}t-\frac{f_{L}^{2}\left(\omega^{2}+\omega_{L}^{2}\right)}{8m\omega_{L}{\left(\omega^{2}-\omega_{L}^{2}\right)}^{2}}\sin\left(2\omega_{L}t\right),\;\mu_{F}=\frac{\Omega_{GP}}{2}+\frac{\Lambda }{2\Omega_{GP}}-\frac{f_{L}^{2}}{4m(\omega^{2}-\omega_{L}^{2})}$$

is a time-dependent phase. Here, $\mu_{F}$ can, in analogy to the static case, be called the quasichemical potential, and is an eigenvalue of the mean-field Floquet Hamiltonian,

(21) $$\left[-\frac{1}{2m}\frac{\partial^{2}}{\partial x^{2}}+\frac{1}{2}m\omega^{2}x^{2}-xf_{L}\cos\left(\omega_{L}t\right)+\Lambda \int dx^{\prime }{\left|\bar{\phi }\left(x^{\prime },t\right)\right|}^{2}{\left(x-x^{\prime }\right)}^{2}-i\frac{\partial }{\partial t}\right]\bar{\phi }\left(x,t\right)=\mu_{F}\bar{\phi }\left(x,t\right).$$

Analogously, $\bar{\phi }\left(x,t\right)$, which may be called the mean-field quasichemical-potential state, is the periodic part of $\phi \left(x,t\right)=e^{-i\mu_{F}t}\bar{\phi }\left(x,t\right)$. Since ${\left|\phi \left(x,t\right)\right|}^{2}={\left|\bar{\phi }\left(x,t\right)\right|}^{2}$, the mean-field Floquet Hamiltonian (21) can be defined solely in terms of $\bar{\phi }\left(x,t\right)$. Of course, the Gross–Pitaevskii solution ϕ(x,t) boils down to ψ(x,t) for non-interacting particles, Λ=0.

The determination of the mean-field wavefunction (17) allows one to prescribe the reduced density matrices. Thus, the time-dependent reduced one-particle density matrix at the mean-field level reads

(22) $$\begin{array}{ccc} & & \rho_{MF}^{\left(1\right)}\left(x,x^{\prime },t\right)=N\rho_{GP}^{\left(1\right)}\left(x,x^{\prime },t\right),\\ & & \rho_{GP}^{\left(1\right)}\left(x,x^{\prime },t\right)={\left(\frac{m\Omega_{GP}}{\pi }\right)}^{\frac{1}{2}}e^{-\frac{m\Omega_{GP}}{2}\left\{{\left[x-x(t)\right]}^{2}+{\left[x^{\prime }-x\left(t\right)\right]}^{2}\right\}}e^{+im\dot{x}\left(t\right)\left\{\left[x-x(t)\right]-\left[x^{\prime }-x\left(t\right)\right]\right\}},\; \end{array}$$

and its diagonal, the density, is $\rho_{MF}^{\left(1\right)}\left(x,t\right)=N\rho_{GP}^{\left(1\right)}\left(x,t\right)$, $\rho_{GP}^{\left(1\right)}\left(x,t\right)={\left(\frac{m\Omega_{GP}}{\pi }\right)}^{\frac{1}{2}}e^{-m\Omega_{GP}{\left[x-x(t)\right]}^{2}}$.

Next, let $\bar{\Phi }\left(x_{1},\ldots ,x_{N},t\right)=\prod_{j=1}^{N}{\bar{\phi }}_{j}\left(x_{j},t\right)$ be the periodic part of the mean-field many-boson wavefunction (17), i.e., $\Phi \left(x_{1},\ldots ,x_{N},t\right)=e^{-iN\mu_{F}t}\bar{\Phi }\left(x_{1},\ldots ,x_{N},t\right)$. Then, the mean-field Floquet energy per particle is defined by sandwiching the many-boson Floquet Hamiltonian,

(23) $$\varepsilon_{F}^{GP}=\frac{\langle \bar{\Phi }|\hat{H}-i\frac{\partial }{\partial t}|\bar{\Phi }\rangle }{N}=\frac{\Omega_{GP}}{2}-\frac{f_{L}^{2}}{4m(\omega^{2}-\omega_{L}^{2})},$$

in analogy to a Bose–Einstein condensate in its ground state for which the mean-field energy per particle is obtained by sandwiching the static many-boson Hamiltonian with the static mean-field wavefunction. We are now in the position to examine the relations between the many-body and mean-field solutions of the driven interacting bosons at the limit of an infinite number of particles.

Thus, the following relations readily hold:

(24) $$\lim_{N\to \infty }\frac{\rho^{\left(1\right)}\left(x,x^{\prime },t\right)}{N}=\rho_{GP}^{\left(1\right)}\left(x,x^{\prime },t\right)$$

for the time-dependent reduced one-particle density matrix per particle, $\lim_{N\to \infty }\frac{\rho^{\left(1\right)}\left(x,t\right)}{N}=\rho_{GP}^{\left(1\right)}\left(x,t\right)$ for the density per particle, and

(25) $$\lim_{N\to \infty }\frac{E_{F}}{N}=\varepsilon_{F}^{GP}$$

for the quasienergy per particle, thus generalizing the literature results for the ground state of trapped interacting bosons at the limit of an infinite number of particles.

## 3. The Driven Harmonic-Interaction Model for Mixtures

Consider a driven mixture of two distinguishable kinds of identical bosons, which we denote as species 1 and species 2. The bosons are trapped in a one-dimensional harmonic potential of frequency ω and interact via harmonic particle–particle interactions. In the present work, we only deal with the ground Floquet solution of the driven mixture. We solve for the case of a generic driven mixture, i.e., a mixture comprising $N_{1}$ bosons of species 1 and mass $m_{1}$ and $N_{2}$ bosons of species 2 and mass $m_{2}$. The total number of bosons is denoted by $N=N_{1}+N_{2}$. The species are driven independently by forces of amplitude $f_{L,1}$ and $f_{L,2}$, respectively. Furthermore, the two intraspecies interaction strengths are denoted by $\lambda_{1}$ and $\lambda_{2}$, and the interspecies interaction strength by $\lambda_{12}$. Positive values of $\lambda_{1}$, $\lambda_{2}$, and $\lambda_{12}$ mean attractive particle–particle interactions, whereas negative values imply repulsive interactions. In what follows, we solve the driven harmonic-interaction model for mixtures at the many-body and mean-field levels, obtain the respective quasienergies, construct the time-dependent intraspecies and interspecies reduced density matrices and densities, discuss properties of these quantities as a function of the interactions between the bosons and strengths of the driving forces, and establish connections between the many-body and mean-field Floquet quantities at the infinite-particle-number limit.

### 3.1. Many-Body Solution and Time-Dependent Reduced Density Matrices

The Hamiltonian of the driven mixture in the laboratory frame is then that of the harmonic-interaction model for mixtures [69] plus two driving terms, and reads

(26) $$\begin{array}{ccc} & & \hat{H}\left(x_{1,1},\ldots ,x_{1,N_{1}},x_{2,1},\ldots ,x_{2,N_{2}},t\right)=\\ & & =\sum_{j=1}^{N_{1}}\left[-\frac{1}{2m_{1}}\frac{\partial^{2}}{\partial x_{1,j}^{2}}+\frac{1}{2}m_{1}\omega^{2}x_{1,j}^{2}-x_{1,j}f_{L,1}\cos\left(\omega_{L}t\right)\right]+\lambda_{1}\sum_{1\leq j<k}^{N_{1}}{\left(x_{1,j}-x_{1,k}\right)}^{2}+\\ & & +\sum_{j=1}^{N_{2}}\left[-\frac{1}{2m_{2}}\frac{\partial^{2}}{\partial x_{2,j}^{2}}+\frac{1}{2}m_{2}\omega^{2}x_{2,j}^{2}-x_{2,j}f_{L,2}\cos\left(\omega_{L}t\right)\right]+\lambda_{2}\sum_{1\leq j<k}^{N_{2}}{\left(x_{2,j}-x_{2,k}\right)}^{2}+\\ & & +\lambda_{12}\sum_{j=1}^{N_{1}}\sum_{k=1}^{N_{2}}{\left(x_{1,j}-x_{2,k}\right)}^{2}.\; \end{array}$$

Here, the coordinates $x_{1,j}$ with subscript 1 stand for species 1 bosons and $x_{2,k}$ with subscript 2 for species 2 bosons. In the present section, we solve the one-dimensional driven mixture, where the amplitudes of the two driving forces $f_{L,1}$ and $f_{L,2}$ are general. In Section 4, we examine an application in two spatial dimensions with $f_{L,2}=0$, i.e., when only species 1 in the interacting mixture is driven.

Using the Jacobi coordinates [109]

(27) $$\begin{array}{ccc} & & Q_{k}=\frac{1}{\sqrt{k(k+1)}}\sum_{j=1}^{k}\left(x_{1,k+1}-x_{1,j}\right),\;1\leq k\leq N_{1}-1,\\ & & Q_{N_{1}-1+k}=\frac{1}{\sqrt{k(k+1)}}\sum_{j=1}^{k}\left(x_{2,k+1}-x_{2,j}\right),\;1\leq k\leq N_{2}-1,\\ & & Q_{N-1}=\sqrt{\frac{N_{2}}{N_{1}}}\sum_{j=1}^{N_{1}}x_{1,j}-\sqrt{\frac{N_{1}}{N_{2}}}\sum_{j=1}^{N_{2}}x_{2,j},\\ & & Q_{N}=\frac{m_{1}}{M}\sum_{j=1}^{N_{1}}x_{1,j}+\frac{m_{2}}{M}\sum_{j=1}^{N_{2}}x_{2,j},\; \end{array}$$

the Hamiltonian (26) transforms to the diagonal form

(28) $$\begin{array}{ccc} & & \hat{H}\left(Q_{1},\ldots ,Q_{N},t\right)=\sum_{k=1}^{N_{1}-1}\left(-\frac{1}{2m_{1}}\frac{\partial^{2}}{\partial Q_{k}^{2}}+\frac{1}{2}m_{1}\Omega_{1}^{2}Q_{k}^{2}\right)+\sum_{k=N_{1}}^{N-2}\left(-\frac{1}{2m_{2}}\frac{\partial^{2}}{\partial Q_{k}^{2}}+\frac{1}{2}m_{2}\Omega_{2}^{2}Q_{k}^{2}\right)+\\ & & +\left[-\frac{1}{2M_{12}}\frac{\partial^{2}}{\partial Q_{N-1}^{2}}+\frac{1}{2}M_{12}\Omega_{12}^{2}Q_{N-1}^{2}-M_{12}\sqrt{N_{1}N_{2}}\left(\frac{f_{L,1}}{m_{1}}-\frac{f_{L,2}}{m_{2}}\right)\cos\left(\omega_{L}t\right)Q_{N-1}\right]+\\ & & +\left[-\frac{1}{2M}\frac{\partial^{2}}{\partial Q_{N}^{2}}+\frac{1}{2}M\omega^{2}Q_{N}^{2}-\left(N_{1}f_{L,1}+N_{2}f_{L,2}\right)\cos\left(\omega_{L}t\right)Q_{N}\right],\; \end{array}$$

where the masses $M_{12}=\frac{m_{1}m_{2}}{M}$, $M=N_{1}m_{1}+N_{2}m_{2}$, and inverse relations $\sum_{j=1}^{N_{1}}x_{1,j}=\sqrt{N_{1}N_{2}}\frac{m_{2}}{M}Q_{N-1}+N_{1}Q_{N}$, $\sum_{j=1}^{N_{1}}x_{2,j}=-\sqrt{N_{1}N_{2}}\frac{m_{1}}{M}Q_{N-1}+N_{2}Q_{N}$ are used. The transformed Hamiltonian of the mixture (28) is that of *N* uncoupled harmonic oscillators, two driven oscillators built on the center-of-mass $Q_{N}$ and relative center-of-mass $Q_{N-1}$ coordinates, and N−2 time-independent oscillators for the relative coordinates of the individual species. The frequencies of the relative coordinates are dressed by the interactions,

(29) $$\begin{array}{ccc} & & \Omega_{1}=\sqrt{\omega^{2}+\frac{2}{m_{1}}\left(N_{1}\lambda_{1}+N_{2}\lambda_{12}\right)},\;\Omega_{2}=\sqrt{\omega^{2}+\frac{2}{m_{2}}\left(N_{2}\lambda_{2}+N_{1}\lambda_{12}\right)},\\ & & \Omega_{12}=\sqrt{\omega^{2}+\frac{2\lambda_{12}}{M_{12}}},\; \end{array}$$

and have to be positive for the driven mixture to be bound, implying the following conditions for the interspecies $\lambda_{12}>-\frac{M_{12}\omega^{2}}{2}$ and the intraspecies $\lambda_{1}>-\frac{N_{2}}{N_{1}}\lambda_{12}-\frac{m_{1}\omega^{2}}{2N_{1}}$ and $\lambda_{2}>-\frac{N_{1}}{N_{2}}\lambda_{12}-\frac{m_{2}\omega^{2}}{2N_{2}}$ interaction strengths.

We can now proceed and prescribe the normalized ground Floquet wavefunction of (28),

(30) $$\begin{array}{ccc} & & \Psi \left(Q_{1},\ldots ,Q_{N},t\right)={\left(\frac{m_{1}\Omega_{1}}{\pi }\right)}^{\frac{N_{1}-1}{4}}{\left(\frac{m_{2}\Omega_{2}}{\pi }\right)}^{\frac{N_{2}-1}{4}}{\left(\frac{M_{12}\Omega_{12}}{\pi }\right)}^{\frac{1}{4}}{\left(\frac{M\omega }{\pi }\right)}^{\frac{1}{4}}\times \\ & & \times e^{-iE(t)}e^{-\frac{1}{2}\left\{m_{1}\Omega_{1}\sum_{k=1}^{N_{1}-1}Q_{k}^{2}+m_{2}\Omega_{2}\sum_{k=N_{1}}^{N-2}Q_{k}^{2}+M_{12}\Omega_{12}{\left[Q_{N-1}-Q_{N-1}\left(t\right)\right]}^{2}+M\omega {\left[Q_{N}-Q_{N}\left(t\right)\right]}^{2}\right\}}\times \\ & & \times e^{+i\left[M_{12}{\dot{Q}}_{N-1}\left(t\right)Q_{N-1}+M{\dot{Q}}_{N}\left(t\right)Q_{N}\right]}=e^{-iE_{F}t}\bar{\Psi }\left(Q_{1},\cdots ,Q_{N},t\right),\; \end{array}$$

where

(31) $$\begin{array}{ccc} & & Q_{N-1}\left(t\right)=\frac{\sqrt{N_{1}N_{2}}\left(\frac{f_{L,1}}{m_{1}}-\frac{f_{L,2}}{m_{2}}\right)}{\Omega_{12}^{2}-\omega_{L}^{2}}\cos\left(\omega_{L}t\right),\;Q_{N}\left(t\right)=\frac{1}{M}\frac{N_{1}f_{L,1}+N_{2}f_{L,2}}{\omega^{2}-\omega_{L}^{2}}\cos\left(\omega_{L}t\right)\; \end{array}$$

are the time-dependent oscillating amplitudes of the relative center-of-mass and center-of-mass coordinates, respectively,

(32) $$\begin{array}{ccc} & & E\left(t\right)=\frac{\left(N_{1}-1\right)\Omega_{1}+\left(N_{2}-1\right)\Omega_{2}+\Omega_{12}+\omega }{2}t+\\ & & +\int^{t}dt^{\prime }\left\{\left[\frac{1}{2}M_{12}{\dot{Q}}_{N-1}^{2}\left(t\right)-\frac{1}{2}M_{12}\Omega_{12}^{2}Q_{N-1}^{2}\left(t\right)\right]+\left[\frac{1}{2}M{\dot{Q}}_{N}^{2}\left(t\right)-\frac{1}{2}M\omega^{2}Q_{N}^{2}\left(t\right)\right]\right\}=\\ & & =E_{F}t-\\ & & -\frac{1}{8\omega_{L}M}\left[\frac{m_{1}m_{2}N_{1}N_{2}{\left(\frac{f_{L,1}}{m_{1}}-\frac{f_{L,2}}{m_{2}}\right)}^{2}\left(\Omega_{12}^{2}+\omega_{L}^{2}\right)}{{\left(\Omega_{12}^{2}-\omega_{L}^{2}\right)}^{2}}+\frac{{\left(N_{1}f_{L,1}+N_{2}f_{L,2}\right)}^{2}\left(\omega^{2}+\omega_{L}^{2}\right)}{{\left(\omega^{2}-\omega_{L}^{2}\right)}^{2}}\right]\sin\left(2\omega_{L}t\right)\; \end{array}$$

is the time-dependent phase, and

(33) $$\begin{array}{ccc} & & E_{F}=\frac{\left(N_{1}-1\right)\Omega_{1}+\left(N_{2}-1\right)\Omega_{2}+\Omega_{12}+\omega }{2}-\\ & & -\frac{1}{4M}\left[\frac{m_{1}m_{2}N_{1}N_{2}{\left(\frac{f_{L,1}}{m_{1}}-\frac{f_{L,2}}{m_{2}}\right)}^{2}}{\Omega_{12}^{2}-\omega_{L}^{2}}+\frac{{\left(N_{1}f_{L,1}+N_{2}f_{L,2}\right)}^{2}}{\omega^{2}-\omega_{L}^{2}}\right]\; \end{array}$$

is the many-particle quasienergy. The quasienergy for the driven mixture (33) is, of course, more intricate than the above quasienergy of the driven Bose–Einstein condensate alone (see (11)). In particular, the appearance of the interspecies interaction-dependent resonance is appealing. For simplicity, in what follows, we assume the off-resonance condition $\omega_{L}\neq \Omega_{12}$ with the relative center-of-mass frequency, in addition to the above-used off-resonance condition $\omega_{L}\neq \omega$ with the center-of-mass frequency.

It is instructive to analyze the solution of the driven mixture. We begin with the poles. There are two poles in the quasienergy; the first is associated with driving the center-of-mass coordinate $Q_{N}$ of the mixture, and the second with driving the relative center-of-mass coordinate $Q_{N-1}$. In the absence of interspecies interaction, the two poles coincide, $\Omega_{12}=\omega$, and the solution boils down to two independent driven Bose–Einstein condensates. Let us move to the zeros. For general forces $f_{L,1}$ and $f_{L,2}$, both the $Q_{N}$ and $Q_{N-1}$ oscillations are activated. There are, however, particular relations between the forces and parameters of the mixture for which either of the oscillations is not activated, i.e., there is zero contribution to the quasienergy. Explicitly, for the relation $\frac{f_{L,1}}{m_{1}}=\frac{f_{L,2}}{m_{2}}$ between the forces and masses, the relative center-of-mass coordinate is not activated, whereas for the relation $f_{L,1}N_{1}+f_{L,2}N_{2}=0$ between the forces and numbers of particles, the center-of-mass coordinate is not activated. Importantly, both modes of oscillations are (non-trivially) activated if only species 1 is driven, i.e., when $f_{L,2}=0$, due to the interspecies interaction. We shall be investigating this particular case in two spatial dimensions in Section 4, focusing on angular-momentum matters.

To move from the Jacobi coordinate to the laboratory-frame representation for the driven mixture, one must express the time-dependent oscillation amplitudes $Q_{N-1}\left(t\right)$ and $Q_{N}\left(t\right)$ in the former representation in terms of corresponding amplitudes for each of the species in the latter. Since each species is made of identical particles, only two such amplitudes are required, which we shall denote by $x_{1}\left(t\right)$ and $x_{2}\left(t\right)$ for species 1 and 2, respectively. Recall that the Jacobi coordinates of the relative motions, $Q_{j},j=1,\ldots ,N-2$, are invariant under translations of each species. Using the definition of the relative center-of-mass and center-of-mass Jacobi coordinates (27), we require $Q_{N-1}-Q_{N-1}\left(t\right)=\sqrt{\frac{N_{2}}{N_{1}}}\sum_{j=1}^{N_{1}}\left[x_{1,j}-x_{1}\left(t\right)\right]-\sqrt{\frac{N_{1}}{N_{2}}}\sum_{j=1}^{N_{2}}\left[x_{2,j}-x_{2}\left(t\right)\right]$ and $Q_{N}-Q_{N}\left(t\right)=\frac{m_{1}}{M}\sum_{j=1}^{N_{1}}\left[x_{1,j}-x_{1}\left(t\right)\right]+\frac{m_{2}}{M}\sum_{j=1}^{N_{2}}\left[x_{2,j}-x_{2}\left(t\right)\right]$ or $\sqrt{N_{1}N_{2}}\left[x_{1}\left(t\right)-x_{2}\left(t\right)\right]=Q_{N-1}\left(t\right)$ and $\frac{1}{M}\left[m_{1}N_{1}x\left(t\right)+m_{2}N_{2}y\left(t\right)\right]=Q_{N}\left(t\right)$. The solutions to these conditions are the translations

(34) $$\begin{array}{ccc} & & x_{1}\left(t\right)=Q_{N}\left(t\right)+\frac{m_{2}}{M}\sqrt{\frac{N_{2}}{N_{1}}}Q_{N-1}\left(t\right)=\frac{1}{M}\left[\frac{N_{1}f_{L,1}+N_{2}f_{L,2}}{\omega^{2}-\omega_{L}^{2}}+\frac{m_{2}N_{2}\left(\frac{f_{L,1}}{m_{1}}-\frac{f_{L,2}}{m_{2}}\right)}{\Omega_{12}^{2}-\omega_{L}^{2}}\right]\cos\left(\omega_{L}t\right),\\ & & x_{2}\left(t\right)=Q_{N}\left(t\right)-\frac{m_{1}}{M}\sqrt{\frac{N_{1}}{N_{2}}}Q_{N-1}\left(t\right)=\frac{1}{M}\left[\frac{N_{1}f_{L,1}+N_{2}f_{L,2}}{\omega^{2}-\omega_{L}^{2}}-\frac{m_{1}N_{1}\left(\frac{f_{L,1}}{m_{1}}-\frac{f_{L,2}}{m_{2}}\right)}{\Omega_{12}^{2}-\omega_{L}^{2}}\right]\cos\left(\omega_{L}t\right),\; \end{array}$$

which are the relations between the individual species’ center-of-mass coordinates and the respective Jacobi coordinates [121]. We see that, in the laboratory frame, both contributing poles (31) are intermixed, and the time-dependent translation of each species is more intricate.

We can now translate the Floquet wavefunction (30) from the Jacobi to Cartesian coordinates,

(35) $$\begin{array}{ccc} & & \Psi \left(x_{1,1},\ldots ,x_{1,N_{1}},x_{2,1},\ldots ,x_{2,N_{2}},t\right)={\left(\frac{m_{1}\Omega_{1}}{\pi }\right)}^{\frac{N_{1}-1}{4}}{\left(\frac{m_{2}\Omega_{2}}{\pi }\right)}^{\frac{N_{2}-1}{4}}{\left(\frac{M_{12}\Omega_{12}}{\pi }\right)}^{\frac{1}{4}}{\left(\frac{M\omega }{\pi }\right)}^{\frac{1}{4}}\times \\ & & \times e^{-iE(t)}e^{+i\left[m_{1}N_{1}{\dot{x}}_{1}\left(t\right)x_{1}\left(t\right)+m_{2}N_{2}{\dot{x}}_{2}\left(t\right)x_{2}\left(t\right)\right]}e^{-\frac{\alpha_{1}}{2}\sum_{j=1}^{N_{1}}{\left[x_{1,j}-x_{1}\left(t\right)\right]}^{2}-\beta_{1}\sum_{1\leq j<k}^{N_{1}}\left[x_{1,j}-x_{1}\left(t\right)\right]\left[x_{1,k}-x_{1}\left(t\right)\right]}\times \\ & & \times e^{-\frac{\alpha_{1}}{2}\sum_{j=1}^{N_{2}}{\left[x_{2,j}-x_{2}\left(t\right)\right]}^{2}-\beta_{2}\sum_{1\leq j<k}^{N_{2}}\left[x_{2,j}-x_{2}\left(t\right)\right]\left[x_{2,k}-x_{2}\left(t\right)\right]}\times e^{+\gamma \sum_{j=1}^{N_{1}}\sum_{k=1}^{N_{2}}\left[x_{1,j}-x_{1}\left(t\right)\right]\left[x_{2,k}-x_{2}\left(t\right)\right]}\times \\ & & \times e^{+i\left\{m_{1}{\dot{x}}_{1}\left(t\right)\sum_{j=1}^{N_{1}}\left[x_{1,j}-x_{1}\left(t\right)\right]+m_{2}{\dot{x}}_{2}\left(t\right)\sum_{j=1}^{N_{2}}\left[x_{2,j}-x_{2}\left(t\right)\right]\right\}},\; \end{array}$$

where the parameters are

(36) $$\begin{array}{ccc} & & \alpha_{1}=m_{1}\Omega_{1}+\beta_{1},\;\beta_{1}=m_{1}\left[-\Omega_{1}\frac{1}{N_{1}}+\left(m_{2}N_{2}\Omega_{12}+m_{1}N_{1}\omega \right)\frac{1}{MN_{1}}\right],\\ & & \alpha_{2}=m_{2}\Omega_{2}+\beta_{2},\;\beta_{2}=m_{2}\left[-\Omega_{2}\frac{1}{N_{2}}+\left(m_{1}N_{1}\Omega_{12}+m_{2}N_{2}\omega \right)\frac{1}{MN_{2}}\right],\\ & & \gamma =M_{12}\left(\Omega_{12}-\omega \right)\; \end{array}$$

and $M_{12}{\dot{Q}}_{N-1}\left(t\right)Q_{N-1}+M{\dot{Q}}_{N}\left(t\right)Q_{N}=m_{1}{\dot{x}}_{1}\left(t\right)\sum_{j=1}^{N_{1}}x_{1,j}+m_{2}{\dot{x}}_{2}\left(t\right)\sum_{j=1}^{N_{2}}x_{2,j}$ is used.

From the time-dependent wavefunction (35,36), the $\left(N_{1}+N_{2}\right)$-particle density matrix of the driven mixture is defined and given by

(37) $$\begin{array}{ccc} & & \Psi \left(x_{1,1},\ldots ,x_{1,N_{1}},x_{2,1},\ldots ,x_{2,N_{2}},t\right)\Psi^{*}\left(x_{1,1}^{\prime },\ldots ,x_{1,N_{1}}^{\prime },x_{2,1}^{\prime },\ldots ,x_{2,N_{2}}^{\prime },t\right)=\\ & & ={\left(\frac{m_{1}\Omega_{1}}{\pi }\right)}^{\frac{N_{1}-1}{2}}{\left(\frac{m_{2}\Omega_{2}}{\pi }\right)}^{\frac{N_{2}-1}{2}}{\left(\frac{M_{12}\Omega_{12}}{\pi }\right)}^{\frac{1}{2}}{\left(\frac{M\omega }{\pi }\right)}^{\frac{1}{2}}\times \\ & & \times e^{-\frac{\alpha_{1}}{2}\sum_{j=1}^{N_{1}}\left\{{\left[x_{1,j}-x_{1}\left(t\right)\right]}^{2}+{\left[x_{1,j}^{\prime }-x_{1}\left(t\right)\right]}^{2}\right\}-\beta_{1}\sum_{1\leq j<k}^{N_{1}}\left\{\left[x_{1,j}-x_{1}\left(t\right)\right]\left[x_{1,k}-x_{1}\left(t\right)\right]+\left[x_{1,j}^{\prime }-x_{1}\left(t\right)\right]\left[x_{1,k}^{\prime }-x_{1}\left(t\right)\right]\right\}}\times \\ & & \times e^{-\frac{\alpha_{1}}{2}\sum_{j=1}^{N_{2}}\left\{{\left[x_{2,j}-x_{2}\left(t\right)\right]}^{2}+{\left[x_{2,j}^{\prime }-x_{2}\left(t\right)\right]}^{2}\right\}-\beta_{2}\sum_{1\leq j<k}^{N_{2}}\left\{\left[x_{2,j}-x_{2}\left(t\right)\right]\left[x_{2,k}-x_{2}\left(t\right)\right]+\left[x_{2,j}^{\prime }-x_{2}\left(t\right)\right]\left[x_{2,k}^{\prime }-x_{2}\left(t\right)\right]\right\}}\times \\ & & \times e^{+\gamma \sum_{j=1}^{N_{1}}\sum_{k=1}^{N_{2}}\left\{\left[x_{1,j}-x_{1}\left(t\right)\right]\left[x_{2,k}-x_{2}\left(t\right)\right]+\left[x_{1,j}^{\prime }-x_{1}\left(t\right)\right]\left[x_{2,k}^{\prime }-x_{2}\left(t\right)\right]\right\}}\times \\ & & \times e^{+im_{1}{\dot{x}}_{1}\left(t\right)\sum_{j=1}^{N_{1}}\left\{\left[x_{1,j}-x_{1}\left(t\right)\right]-\left[x_{1,j}^{\prime }-x_{1}\left(t\right)\right]\right\}}e^{+im_{2}{\dot{x}}_{2}\left(t\right)\sum_{j=1}^{N_{2}}\left\{\left[x_{2,j}-x_{2}\left(t\right)\right]-\left[x_{2,j}^{\prime }-x_{2}\left(t\right)\right]\right\}}. \end{array}$$

This allows us to compute the reduced density matrices of the driven mixture.

It turns out that the determination of the time-dependent reduced density matrices of the driven mixture follows in practically the same way as for the undriven mixtures [69], although this is not a straightforward result. The driving forces are coupled to the relative center-of-mass $Q_{N-1}$ and center-of-mass $Q_{N}$ coordinates only. Consequently, as shown above, all coordinates of species 1 are translated by the same amplitude $x_{1}\left(t\right)$, and likewise, all coordinates of species 2 are translated by $x_{2}\left(t\right)$. Performing the integrations consecutively over $x_{1,j},x_{1,j}^{\prime }=x_{1,j},j=N_{1},\ldots ,2,1$ and $x_{2,k},x_{2,k}^{\prime }=x_{2,k},k=N_{2},\ldots ,2,1$, for which the spatial-dependent phase factors are canceled, and while changing the variables $x_{1,j}-x_{1}\left(t\right)\to x_{1,j}$ and $x_{2,k}-x_{2}\left(t\right)\to x_{2,k}$, one finds the same recurrence relations [69] determining the coefficients of the now time-dependent reduced density matrices (see below). As a result of this, the coherence properties of the driven mixture described by the ground Floquet wavefunction (35,36), like the eigenvalues of the time-dependent intraspecies reduced one-particle density matrices and interspecies reduced two-particle density matrix, are exactly those of the undriven mixture; in particular, these eigenvalues are time independent. Thus, the time-dependent intraspecies reduced one-particle density matrices are explicitly given as

(38) $$\begin{array}{ccc} & & \rho_{1}^{\left(1\right)}\left(x_{1},x_{1}^{\prime },t\right)=N_{1}{\left(\frac{\alpha +C_{1,0}}{\pi }\right)}^{\frac{1}{2}}e^{-\frac{\alpha_{1}}{2}\left\{{\left[x_{1}-x_{1}\left(t\right)\right]}^{2}+{\left[x_{1}^{\prime }-x_{1}\left(t\right)\right]}^{2}\right\}}\times \\ & & \times e^{-\frac{1}{4}C_{1,0}{\left\{\left[x_{1}-x_{1}\left(t\right)\right]+\left[x_{1}^{\prime }-x_{1}\left(t\right)\right]\right\}}^{2}}e^{+im_{1}{\dot{x}}_{1}\left(t\right)\left\{\left[x_{1}-x_{1}\left(t\right)\right]-\left[x_{1}^{\prime }-x_{1}\left(t\right)\right]\right\}},\\ & & C_{1,0}=\frac{\left(\alpha_{1}-\beta_{1}\right)C_{N_{1},0}-\left(N_{1}-1\right)\left(C_{N_{1},0}+\beta_{1}\right)\beta_{1}}{\left(\alpha_{1}-\beta_{1}\right)+\left(N_{1}-1\right)\left(C_{N_{1},0}+\beta_{1}\right)},\;C_{N_{1},0}=-\gamma^{2}\frac{N_{2}}{\left(\alpha_{2}-\beta_{2}\right)+N_{2}\beta_{2}},\\ & & \rho_{2}^{\left(1\right)}\left(x_{2},x_{2}^{\prime },t\right)=N_{2}{\left(\frac{\alpha +C_{0,1}^{\prime }}{\pi }\right)}^{\frac{1}{2}}e^{-\frac{\alpha_{2}}{2}\left\{{\left[x_{2}-x_{2}\left(t\right)\right]}^{2}+{\left[x_{2}^{\prime }-x_{2}\left(t\right)\right]}^{2}\right\}}\times \\ & & e^{-\frac{1}{4}C_{0,1}^{\prime }{\left\{\left[x_{2}-x_{2}\left(t\right)\right]+\left[x_{2}^{\prime }-x_{2}\left(t\right)\right]\right\}}^{2}}e^{+im_{2}{\dot{x}}_{2}\left(t\right)\left\{\left[x_{2}-x_{2}\left(t\right)\right]-\left[x_{2}^{\prime }-x_{2}\left(t\right)\right]\right\}},\\ & & C_{0,1}^{\prime }=\frac{\left(\alpha_{2}-\beta_{2}\right)C_{0,N_{2}}^{\prime }-\left(N_{2}-1\right)\left(C_{0,N_{2}}^{\prime }+\beta_{2}\right)\beta_{2}}{\left(\alpha_{2}-\beta_{2}\right)+\left(N_{2}-1\right)\left(C_{0,N_{2}}^{\prime }+\beta_{2}\right)},\;C_{0,N_{2}}^{\prime }=-\gamma^{2}\frac{N_{1}}{\left(\alpha_{1}-\beta_{1}\right)+N_{1}\beta_{1}}.\; \end{array}$$

The diagonals of $\rho_{1}^{\left(1\right)}\left(x_{1},x_{1}^{\prime },t\right)$ and $\rho_{2}^{\left(1\right)}\left(x_{2},x_{2}^{\prime },t\right)$ are the intraspecies time-dependent densities and are given explicitly by

(39) $$\begin{array}{ccc} & & \rho_{1}^{\left(1\right)}\left(x_{1},t\right)=N_{1}{\left(\frac{\alpha_{1}+C_{1,0}}{\pi }\right)}^{\frac{1}{2}}e^{-\left(\alpha_{1}+C_{1,0}\right){\left[x_{1}-x_{1}\left(t\right)\right]}^{2}}=\\ & & =N_{1}{\left(\frac{\alpha_{1}+C_{1,0}}{\pi }\right)}^{\frac{1}{2}}e^{-\left(\alpha_{1}+C_{1,0}\right){\left\{x-\frac{1}{M}\left[\frac{N_{1}f_{L,1}+N_{2}f_{L,2}}{\omega^{2}-\omega_{L}^{2}}+\frac{m_{2}N_{2}\left(\frac{f_{L,1}}{m_{1}}-\frac{f_{L,2}}{m_{2}}\right)}{\Omega_{12}^{2}-\omega_{L}^{2}}\right]\cos\left(\omega_{L}t\right)\right\}}^{2}},\\ & & \rho_{2}^{\left(1\right)}\left(x_{2},t\right)=N_{2}{\left(\frac{\alpha_{2}+C_{0,1}^{\prime }}{\pi }\right)}^{\frac{1}{2}}e^{-\left(\alpha_{2}+C_{0,1}^{\prime }\right){\left[x_{2}-x_{2}\left(t\right)\right]}^{2}}=\\ & & =N_{2}{\left(\frac{\alpha_{2}+C_{0,1}^{\prime }}{\pi }\right)}^{\frac{1}{2}}e^{-\left(\alpha_{2}+C_{0,1}^{\prime }\right){\left\{x-\frac{1}{M}\left[\frac{N_{1}f_{L,1}+N_{2}f_{L,2}}{\omega^{2}-\omega_{L}^{2}}-\frac{m_{1}N_{1}\left(\frac{f_{L,1}}{m_{1}}-\frac{f_{L,2}}{m_{2}}\right)}{\Omega_{12}^{2}-\omega_{L}^{2}}\right]\cos\left(\omega_{L}t\right)\right\}}^{2}}.\; \end{array}$$

We see that the two species generally oscillate with different amplitudes due to the intermixing of the two poles, with the specific cases of a single-pole contribution when only the center-of-mass (same-amplitude motion; for $\frac{f_{L,1}}{m_{1}}=\frac{f_{L,2}}{m_{2}}$) or relative center-of-mass (anti-phase motion; for $f_{L,1}N_{1}+f_{L,2}N_{2}=0$) coordinate is activated.

Finally, the time-dependent interspecies reduced two-particle density matrix ofthe mixture, $\rho_{12}^{\left(2\right)}\left(x_{1},x_{1}^{\prime },x_{2},x_{2}^{\prime },t\right)=N_{1}N_{2}\int dx_{1,2}\cdots dx_{1,N_{1}}dx_{2,2}\cdots dx_{2,N_{2}}\times$$\times \Psi \left(x_{1},x_{1,2},\ldots ,x_{1,N_{1}},x_{2},x_{2,2},\ldots ,x_{2,N_{2}},t\right)\Psi^{*}\left(x_{1}^{\prime },x_{1,2},\ldots ,x_{1,N_{1}},x_{2}^{\prime },x_{2,2},\ldots ,x_{2,N_{2}},t\right)$, which is the lowest-order interspecies quantity, is given by

(40) $$\begin{array}{ccc} & & \rho_{12}^{\left(2\right)}\left(x_{1},x_{1}^{\prime },x_{2},x_{2}^{\prime },t\right)=N_{1}N_{2}{\left[\frac{\left(\alpha_{1}+C_{1,1}\right)\left(\alpha_{2}+C_{1,1}^{\prime }\right)-D_{1,1}^{2}}{\pi^{2}}\right]}^{\frac{1}{2}}\times \\ & & \times e^{-\frac{\alpha_{1}}{2}\left\{{\left[x_{1}-x_{1}\left(t\right)\right]}^{2}+{\left[x_{1}^{\prime }-x_{1}\left(t\right)\right]}^{2}\right\}}e^{-\frac{\alpha_{2}}{2}\left\{{\left[x_{2}-x_{2}\left(t\right)\right]}^{2}+{\left[x_{2}^{\prime }-x_{2}\left(t\right)\right]}^{2}\right\}}e^{-\frac{1}{4}C_{1,1}{\left\{\left[x_{1}-x_{1}\left(t\right)\right]+\left[x_{1}^{\prime }-x_{1}\left(t\right)\right]\right\}}^{2}}\times \\ & & e^{-\frac{1}{4}C_{1,1}^{\prime }{\left\{\left[x_{2}-x_{2}\left(t\right)\right]+\left[x_{2}^{\prime }-x_{2}\left(t\right)\right]\right\}}^{2}}e^{+\frac{1}{2}D_{1,1}\left\{\left[x_{1}-x_{1}\left(t\right)\right]+\left[x_{1}^{\prime }-x_{1}\left(t\right)\right]\right\}\left\{{\left[x_{2}-x_{2}\left(t\right)\right]}^{2}+\left[x_{2}^{\prime }-x_{2}\left(t\right)\right]\right\}}\times \\ & & \times e^{+\frac{1}{2}D_{1,1}^{\prime }\left\{\left[x_{1}-x_{1}\left(t\right)\right]-\left[x_{1}^{\prime }-x_{1}\left(t\right)\right]\right\}\left\{{\left[x_{2}-x_{2}\left(t\right)\right]}^{2}-\left[x_{2}^{\prime }-x_{2}\left(t\right)\right]\right\}}e^{+im_{1}{\dot{x}}_{1}\left(t\right)\left\{\left[x_{1}-x_{1}\left(t\right)\right]-\left[x_{1}^{\prime }-x_{1}\left(t\right)\right]\right\}}\times \\ & & \times e^{+im_{2}{\dot{x}}_{2}\left(t\right)\left\{\left[x_{2}-x_{2}\left(t\right)\right]-\left[x_{2}^{\prime }-x_{2}\left(t\right)\right]\right\}},\; \end{array}$$

where

(41) $$\begin{array}{ccc} & & C_{1,1}=\frac{\left(\alpha_{1}-\beta_{1}\right)C_{N_{1},1}-\left(N_{1}-1\right)\left(C_{N_{1},1}+\beta_{1}\right)\beta_{1}}{\left(\alpha_{1}-\beta_{1}\right)+\left(N_{1}-1\right)\left(C_{N_{1},1}+\beta_{1}\right)},\;C_{N_{1},1}=-\gamma^{2}\frac{N_{2}-1}{\left(\alpha_{2}-\beta_{2}\right)+\left(N_{2}-1\right)\beta_{2}},\\ & & C_{1,1}^{\prime }=\frac{\left(\alpha_{2}-\beta_{2}\right)C_{1,N_{2}}^{\prime }-\left(N_{2}-1\right)\left(C_{1,N_{2}}^{\prime }+\beta_{2}\right)\beta_{2}}{\left(\alpha_{2}-\beta_{2}\right)+\left(N_{2}-1\right)\left(C_{1,N_{2}}^{\prime }+\beta_{2}\right)},\;C_{1,N_{2}}^{\prime }=-\gamma^{2}\frac{N_{1}-1}{\left(\alpha_{1}-\beta_{1}\right)+\left(N_{1}-1\right)\beta_{1}},\\ & & D_{1,1}=\gamma \frac{\left(\alpha_{1}-\beta_{1}\right)\left(\alpha_{2}-\beta_{2}\right)}{\left[\left(\alpha_{1}-\beta_{1}\right)+\left(N_{1}-1\right)\beta_{1}\right]\left[\left(\alpha_{2}-\beta_{2}\right)+\left(N_{2}-1\right)\beta_{2}\right]-\gamma^{2}\left(N_{1}-1\right)\left(N_{2}-1\right)},\\ & & D_{1,1}^{\prime }=\gamma . \end{array}$$

Then, the diagonal part of $\rho_{12}^{\left(2\right)}\left(x_{1},x_{1}^{\prime },x_{2},x_{2}^{\prime },t\right)$ is the time-dependent two-particle interspecies density and reads

(42) $$\begin{array}{ccc} & & \rho_{12}^{\left(2\right)}\left(x_{1},x_{2},t\right)=N_{1}N_{2}{\left[\frac{\left(\alpha_{1}+C_{1,1}\right)\left(\alpha_{2}+C_{1,1}^{\prime }\right)-D_{1,1}^{2}}{\pi^{2}}\right]}^{\frac{1}{2}}\times \\ & & \times e^{-\left(\alpha_{1}+C_{1,1}\right){\left[x_{1}-x_{1}\left(t\right)\right]}^{2}}e^{-\left(\alpha_{2}+C_{1,1}^{\prime }\right){\left[x_{2}-x_{2}\left(t\right)\right]}^{2}}e^{+2D_{1,1}\left[x_{1}-x_{1}\left(t\right)\right]\left[x_{2}-x_{2}\left(t\right)\right]}=\\ & & =N_{1}N_{2}{\left[\frac{\left(\alpha_{1}+C_{1,1}\right)\left(\alpha_{2}+C_{1,1}^{\prime }\right)-D_{1,1}^{2}}{\pi^{2}}\right]}^{\frac{1}{2}}\times \\ & & \times e^{-\left(\alpha_{1}+C_{1,1}\right){\left\{x_{1}-\frac{1}{M}\left[\frac{N_{1}f_{L,1}+N_{2}f_{L,2}}{\omega^{2}-\omega_{L}^{2}}+\frac{m_{2}N_{2}\left(\frac{f_{L,1}}{m_{1}}-\frac{f_{L,2}}{m_{2}}\right)}{\Omega_{12}^{2}-\omega_{L}^{2}}\right]\cos\left(\omega_{L}t\right)\right\}}^{2}}\times \\ & & \times e^{-\left(\alpha_{2}+C_{1,1}^{\prime }\right){\left\{x_{2}-\frac{1}{M}\left[\frac{N_{1}f_{L,1}+N_{2}f_{L,2}}{\omega^{2}-\omega_{L}^{2}}-\frac{m_{1}N_{1}\left(\frac{f_{L,1}}{m_{1}}-\frac{f_{L,2}}{m_{2}}\right)}{\Omega_{12}^{2}-\omega_{L}^{2}}\right]\cos\left(\omega_{L}t\right)\right\}}^{2}}\times \\ & & \;\;\;\;\;\;\;\;\;\;\;\times e^{+2D_{1,1}\left\{x_{1}-\frac{1}{M}\left[\frac{N_{1}f_{L,1}+N_{2}f_{L,2}}{\omega^{2}-\omega_{L}^{2}}+\frac{m_{2}N_{2}\left(\frac{f_{L,1}}{m_{1}}-\frac{f_{L,2}}{m_{2}}\right)}{\Omega_{12}^{2}-\omega_{L}^{2}}\right]\cos\left(\omega_{L}t\right)\right\}\left\{x_{2}-\frac{1}{M}\left[\frac{N_{1}f_{L,1}+N_{2}f_{L,2}}{\omega^{2}-\omega_{L}^{2}}-\frac{m_{1}N_{1}\left(\frac{f_{L,1}}{m_{1}}-\frac{f_{L,2}}{m_{2}}\right)}{\Omega_{12}^{2}-\omega_{L}^{2}}\right]\cos\left(\omega_{L}t\right)\right\}}.\; \end{array}$$

The coupling term in $\rho_{12}^{\left(2\right)}\left(x_{1},x_{2},t\right)$ stems from the interspecies interaction and renders the time-dependent two-particle density not separable at the many-body level of theory. Furthermore, it mixes the time-dependent oscillation amplitudes $x_{1}\left(t\right)$ and $x_{2}\left(t\right)$ of both species. This could raise interesting questions pertaining to the intensities of $\omega_{L}$-harmonics in many-body quantities describing driven mixtures, which are left for investigations elsewhere.

### 3.2. Mean-Field Solution and the Limit of an Infinite Number of Particles

We are now in the position to solve the driven mixture at the mean-field level, and thereafter to compare the many-body and mean-field outcomes at the limit of an infinite number of particles. The related structures of the time-dependent reduced one-particle density matrices per particle of the mixture’s ground Floquet state (38) and of the static reduced one-particle density matrices per particle of the mixture’s ground state [69] already tell us that at the infinite-particle-number limit, each of the driven species is 100% condensed. Furthermore and on similar grounds, the time-dependent interspecies reduced two-particle density matrix per particle (40,41) is separable and given by the product of the time-dependent intraspecies reduced one-particle density matrices per particle at the limit of an infinite number of particles. To obtain further properties of the driven mixture at this limit, we now derive the solution of the driven harmonic-interaction model for mixtures at the mean-field level of theory. This would allow us to explicitly compare the respective intraspecies and interspecies time-dependent reduced density matrices per particle and quasienergies per particle and identify the connection between many-body and mean-field levels of theory for driven interacting mixtures, at least within the driven harmonic-interaction model for mixtures. Beyond the above, the Floquet solution of the coupled non-linear Schrödinger equations is interesting and instrumental for itself.

The time-dependent many-body wavefunction is given at the mean-field level by

(43) $$\Phi \left(x_{1,1},\ldots ,x_{1,N_{1}},x_{2,1},\ldots ,x_{2,N_{2}},t\right)=\prod_{j=1}^{N_{1}}\phi_{1}\left(x_{1,j},t\right)\prod_{k=1}^{N_{2}}\phi_{2}\left(x_{2,k},t\right).$$

All species 1 bosons are described by one and the same time-dependent orbital $\phi_{1}\left(x_{1},t\right)$, and all species 2 bosons are similarly described by another time-dependent orbital $\phi_{2}\left(x_{2},t\right)$, which are to be determined self-consistently. Employing the time-dependent variational principle in any of its forms leads to the coupled time-dependent Gross–Pitaevskii equations

(44) $$\begin{array}{ccc} & & \{-\frac{1}{2m_{1}}\frac{\partial^{2}}{\partial x_{1}^{2}}+\frac{1}{2}m_{1}\omega^{2}x_{1}^{2}-x_{1}f_{L,1}\cos\left(\omega_{L}t\right)+\\ & & +\Lambda_{1}\int dx_{1}^{\prime }|\phi_{1}\left(x_{1}^{\prime },t\right){|}^{2}{\left(x_{1}-x_{1}^{\prime }\right)}^{2}+\Lambda_{21}\int dx_{2}{\left|\phi_{2}\left(x_{2},t\right)\right|}^{2}{\left(x_{1}-x_{2}\right)}^{2}\}\phi_{1}\left(x_{1},t\right)=i\frac{\partial \phi_{1}\left(x_{1},t\right)}{\partial t},\\ & & \{-\frac{1}{2m_{2}}\frac{\partial^{2}}{\partial x_{2}^{2}}+\frac{1}{2}m_{2}\omega^{2}x_{2}^{2}-x_{2}f_{L,2}\cos\left(\omega_{L}t\right)+\\ & & +\Lambda_{2}\int dx_{2}^{\prime }|\phi_{2}\left(x_{2}^{\prime },t\right){|}^{2}{\left(x_{2}-x_{2}^{\prime }\right)}^{2}+\Lambda_{12}\int dx_{1}{\left|\phi_{1}\left(x_{1},t\right)\right|}^{2}{\left(x_{1}-x_{2}\right)}^{2}\}\phi_{2}\left(x_{2},t\right)=i\frac{\partial \phi_{2}\left(x_{2},t\right)}{\partial t},\; \end{array}$$

where the mean-field interaction parameters are given by $\Lambda_{1}=\lambda_{1}\left(N_{1}-1\right)$, $\Lambda_{2}=\lambda_{1}\left(N_{2}-1\right)$, $\Lambda_{12}=\lambda_{12}N_{1}$, and $\Lambda_{21}=\lambda_{12}N_{2}$, and satisfy $N_{1}\Lambda_{21}=N_{2}\Lambda_{12}$. Only the interaction parameters appear within the mean-field treatment of the (driven) mixture. To compare the many-body and mean-field treatments, the mean-field interaction parameters are held fixed, while the numbers of particles $N_{1}$ and $N_{2}$ are increased towards the infinite-particle-number limit. In this way, the total number of particles $N=N_{1}+N_{2}$ is increased to infinity while the ratios $\frac{N_{1}}{N_{2}}=\frac{\Lambda_{12}}{\Lambda_{21}}$, $\frac{N_{1}}{N}=\frac{\Lambda_{12}}{\Lambda_{12}+\Lambda_{21}}$, $\frac{N_{2}}{N}=\frac{\Lambda_{21}}{\Lambda_{12}+\Lambda_{21}}$, defined using the (non-zero) intraspecies interaction strength $\lambda_{12}$, are held fixed.

The solution of the time-dependent coupled non-linear Schrödinger Equation (44) is somewhat involved, and for brevity and completeness it is detailed in Appendix B. The final result for the time-dependent mean-field orbitals can be written as

(45) $$\begin{array}{ccc} & & \phi_{1}\left(x_{1},t\right)={\left(\frac{m_{1}\Omega_{1,GP}}{\pi }\right)}^{\frac{1}{4}}e^{-i\mu_{1}\left(t\right)}\times \\ & & \times e^{-\frac{m_{1}\Omega_{1,GP}}{2}{\left[x_{1}-\frac{1}{m_{1}\Lambda_{12}+m_{2}\Lambda_{21}}\left[\frac{\Lambda_{12}f_{L,1}+\Lambda_{21}f_{L,2}}{\omega^{2}-\omega_{L}^{2}}+\frac{m_{2}\Lambda_{21}\left(\frac{f_{L,1}}{m_{1}}-\frac{f_{L,2}}{m_{2}}\right)}{\Omega_{12}^{2}-\omega_{L}^{2}}\right]\cos\left(\omega_{L}t\right)\right]}^{2}}\times \\ & & \times e^{-i\frac{m_{1}\omega_{L}}{m_{1}\Lambda_{12}+m_{2}\Lambda_{21}}\left[\frac{\Lambda_{12}f_{L,1}+\Lambda_{21}f_{L,2}}{\omega^{2}-\omega_{L}^{2}}+\frac{m_{2}\Lambda_{21}\left(\frac{f_{L,1}}{m_{1}}-\frac{f_{L,2}}{m_{2}}\right)}{\Omega_{12}^{2}-\omega_{L}^{2}}\right]\sin\left(\omega_{L}t\right)x_{1}}=\\ & & ={\left(\frac{m_{1}\Omega_{1,GP}}{\pi }\right)}^{\frac{1}{4}}e^{-i\mu_{1}\left(t\right)}e^{-\frac{m_{1}\Omega_{1,GP}}{2}{\left[x_{1}-x_{1}\left(t\right)\right]}^{2}}e^{+im_{1}{\dot{x}}_{1}\left(t\right)x_{1}},\\ & & \phi_{2}\left(x_{2},t\right)={\left(\frac{m_{2}\Omega_{2,GP}}{\pi }\right)}^{\frac{1}{4}}e^{-i\mu_{2}\left(t\right)}\times \\ & & \times e^{-\frac{m_{2}\Omega_{2,GP}}{2}{\left[x_{2}-\frac{1}{m_{1}\Lambda_{12}+m_{2}\Lambda_{21}}\left[\frac{\Lambda_{12}f_{L,1}+\Lambda_{21}f_{L,2}}{\omega^{2}-\omega_{L}^{2}}-\frac{m_{1}\Lambda_{12}\left(\frac{f_{L,1}}{m_{1}}-\frac{f_{L,2}}{m_{2}}\right)}{\Omega_{12}^{2}-\omega_{L}^{2}}\right]\cos\left(\omega_{L}t\right)\right]}^{2}}\times \\ & & \times e^{-i\frac{m_{2}\omega_{L}}{m_{1}\Lambda_{12}+m_{2}\Lambda_{21}}\left[\frac{\Lambda_{12}f_{L,1}+\Lambda_{21}f_{L,2}}{\omega^{2}-\omega_{L}^{2}}-\frac{m_{1}\Lambda_{12}\left(\frac{f_{L,1}}{m_{1}}-\frac{f_{L,2}}{m_{2}}\right)}{\Omega_{12}^{2}-\omega_{L}^{2}}\right]\sin\left(\omega_{L}t\right)x_{2}}=\\ & & ={\left(\frac{m_{2}\Omega_{2,GP}}{\pi }\right)}^{\frac{1}{4}}e^{-i\mu_{2}\left(t\right)}e^{-\frac{m_{2}\Omega_{2,GP}}{2}{\left[x_{2}-x_{2}\left(t\right)\right]}^{2}}e^{+im_{2}{\dot{x}}_{2}\left(t\right)x_{2}},\; \end{array}$$

where $\Omega_{1,GP}=\sqrt{\omega^{2}+\frac{2(\Lambda_{1}+\Lambda_{21})}{m_{1}}}$ and $\Omega_{2,GP}=\sqrt{\omega^{2}+\frac{2(\Lambda_{2}+\Lambda_{12})}{m_{2}}}$ are interaction-dressed frequencies. The main finding at this point (see Appendix B for details) is that the time-dependent mean-field orbitals (45) oscillate with the same amplitudes, $x_{1}\left(t\right)$ and $x_{2}\left(t\right)$, found in the many-body solution (see (34)), and are otherwise similar to the ground-state mean-field orbitals of the static problem being dressed by both the intraspecies and intersepcies interactions [69]. These results suggest that the respective many-body and mean-field time-dependent reduced density matrices per particle of the driven mixture are to coincide at the limit of an infinite number of particles (see explicitly below). More delicate and intricate are the respective relations of the quasienergies.

As in the driven single-species case, the time-dependent phases $\mu_{1}\left(t\right)$ and $\mu_{2}\left(t\right)$ in (45) consist of periodic and linear in *t* parts. The former do not enter the quasienergy and can be read off (A20) in Appendix B, and the latter are given explicitly as

(46) $$\begin{array}{ccc} & & \mu_{1,F}=\left(\frac{\Omega_{1,GP}}{2}+\frac{\Lambda_{1}}{2\Omega_{1,GP}}+\frac{\Lambda_{21}}{2\Omega_{2,GP}}\right)-\frac{1}{4(m_{1}\Lambda_{12}+m_{2}\Lambda_{21})}\{\frac{m_{1}}{m_{1}\Lambda_{12}+m_{2}\Lambda_{21}}\left(\omega^{2}-\omega_{L}^{2}\right)\times \\ & & \times {\left[\frac{\Lambda_{12}f_{L,1}+\Lambda_{21}f_{L,2}}{\omega^{2}-\omega_{L}^{2}}+\frac{m_{2}\Lambda_{21}\left(\frac{f_{L,1}}{m_{1}}-\frac{f_{L,2}}{m_{2}}\right)}{\Omega_{12}^{2}-\omega_{L}^{2}}\right]}^{2}+\\ & & +2\Lambda_{21}\left[2\;\frac{\Lambda_{12}f_{L,1}+\Lambda_{21}f_{L,2}}{\omega^{2}-\omega_{L}^{2}}\;\frac{\frac{f_{L_{1}}}{m_{1}}-\frac{f_{L_{2}}}{m_{2}}}{\Omega_{12}^{2}-\omega_{L}^{2}}+\left(m_{2}\Lambda_{21}-m_{1}\Lambda_{12}\right){\left(\frac{\frac{f_{L,1}}{m_{1}}-\frac{f_{L,2}}{m_{2}}}{\Omega_{12}^{2}-\omega_{L}^{2}}\right)}^{2}\right]\},\\ & & \mu_{2,F}=\left(\frac{\Omega_{2,GP}}{2}+\frac{\Lambda_{2}}{2\Omega_{2,GP}}+\frac{\Lambda_{12}}{2\Omega_{1,GP}}\right)-\frac{1}{4(m_{1}\Lambda_{12}+m_{2}\Lambda_{21})}\{\frac{m_{2}}{m_{1}\Lambda_{12}+m_{2}\Lambda_{21}}\left(\omega^{2}-\omega_{L}^{2}\right)\times \\ & & \times {\left[\frac{\Lambda_{12}f_{L,1}+\Lambda_{21}f_{L,2}}{\omega^{2}-\omega_{L}^{2}}-\frac{m_{1}\Lambda_{12}\left(\frac{f_{L,1}}{m_{1}}-\frac{f_{L,2}}{m_{2}}\right)}{\Omega_{12}^{2}-\omega_{L}^{2}}\right]}^{2}-\\ & & -2\Lambda_{12}\left[2\;\frac{\Lambda_{12}f_{L,1}+\Lambda_{21}f_{L,2}}{\omega^{2}-\omega_{L}^{2}}\;\frac{\frac{f_{L_{1}}}{m_{1}}-\frac{f_{L_{2}}}{m_{2}}}{\Omega_{12}^{2}-\omega_{L}^{2}}+\left(m_{2}\Lambda_{21}-m_{1}\Lambda_{12}\right){\left(\frac{\frac{f_{L,1}}{m_{1}}-\frac{f_{L,2}}{m_{2}}}{\Omega_{12}^{2}-\omega_{L}^{2}}\right)}^{2}\right]\}.\; \end{array}$$

The quantities $\mu_{1,F}$ and $\mu_{2,F}$ may be called quasichemical potentials of the driven mixture, and are simultaneous eigenvalues of the coupled non-linear Floquet equations

(47) $$\begin{array}{ccc} & & \{-\frac{1}{2m_{1}}\frac{\partial^{2}}{\partial x_{1}^{2}}+\frac{1}{2}m_{1}\omega^{2}x_{1}^{2}-x_{1}f_{L,1}\cos\left(\omega_{L}t\right)+\Lambda_{1}\int dx_{1}^{\prime }{\left|{\bar{\phi }}_{1}\left(x_{1}^{\prime },t\right)\right|}^{2}{\left(x_{1}-x_{1}^{\prime }\right)}^{2}+\\ & & +\Lambda_{21}\int dx_{2}{\left|{\bar{\phi }}_{2}\left(x_{2},t\right)\right|}^{2}{\left(x_{1}-x_{2}\right)}^{2}-i\frac{\partial }{\partial t}\}{\bar{\phi }}_{1}\left(x_{1},t\right)=\mu_{1,F}{\bar{\phi }}_{1}\left(x_{1},t\right),\\ & & \{-\frac{1}{2m_{2}}\frac{\partial^{2}}{\partial x_{2}^{2}}+\frac{1}{2}m_{2}\omega^{2}x_{2}^{2}-x_{2}f_{L,2}\cos\left(\omega_{L}t\right)+\Lambda_{2}\int dx_{2}^{\prime }{\left|{\bar{\phi }}_{2}\left(x_{2}^{\prime },t\right)\right|}^{2}{\left(x_{2}-x_{2}^{\prime }\right)}^{2}+\\ & & +\Lambda_{12}\int dx_{1}{\left|{\bar{\phi }}_{1}\left(x_{1},t\right)\right|}^{2}{\left(x_{1}-x_{2}\right)}^{2}-i\frac{\partial }{\partial t}\}{\bar{\phi }}_{2}\left(x_{2},t\right)=\mu_{2,F}{\bar{\phi }}_{2}\left(x_{2},t\right),\; \end{array}$$

where ${\bar{\phi }}_{1}\left(x_{1},t\right)$ and ${\bar{\phi }}_{1}\left(x_{2},t\right)$ are the corresponding periodic parts of the time-dependent orbitals, $\phi_{1}\left(x_{1},t\right)=e^{-i\mu_{1,F}t}{\bar{\phi }}_{1}\left(x_{1},t\right)$ and $\phi_{2}\left(x_{2},t\right)=e^{-i\mu_{2,F}t}{\bar{\phi }}_{2}\left(x_{2},t\right)$. Thus, when solved at the mean-field level of theory, a driven mixture gives rise to coupled non-linear Floquet equations whose eigenvalues are analogs of the chemical potentials of a static mixture.

As can be seen from (46), the quasichemical potentials $\mu_{1,F}$ and $\mu_{2,F}$ are built from two parts. The first is the contribution of the static problem to the solution and is dressed by the intraspecies and interspecies interactions, and the second originates from the driving forces and depends on these forces and the interspecies interaction only. In particular, the poles’ structure of $\mu_{1,F}$ and $\mu_{2,F}$ would play a central role in the mean-field quasienergy and its relation to the many-body quasienergy (33).

With the mean-field wavefunction (43) computed, we proceed to the time-dependent reduced density matrices and thereafter to the limit of an infinite number of particles. Thus, the lowest-order intraspecies and interspecies reduced density matrices are given at the mean-field level by

(48) $$\begin{array}{ccc} & & \rho_{1,MF}^{\left(1\right)}\left(x_{1},x_{1}^{\prime },t\right)=N_{1}\rho_{1,GP}^{\left(1\right)}\left(x_{1},x_{1}^{\prime },t\right),\;\rho_{1,GP}^{\left(1\right)}\left(x_{1},x_{1}^{\prime },t\right)={\left(\frac{m_{1}\Omega_{1,GP}}{\pi }\right)}^{\frac{1}{2}}\times \\ & & \times e^{-\frac{m_{1}\Omega_{1,GP}}{2}\left\{{\left[x_{1}-x_{1}\left(t\right)\right]}^{2}+{\left[x_{1}^{\prime }-x_{1}\left(t\right)\right]}^{2}\right\}}e^{+im_{1}\dot{x_{1}}\left(t\right)\left\{\left[x_{1}-x_{1}\left(t\right)\right]-\left[x_{1}^{\prime }-x_{1}\left(t\right)\right]\right\}},\\ & & \rho_{2,MF}^{\left(1\right)}\left(x_{2},x_{2}^{\prime },t\right)=N_{2}\rho_{2,GP}^{\left(1\right)}\left(x_{2},x_{2}^{\prime },t\right),\;\rho_{2,GP}^{\left(1\right)}\left(x_{2},x_{2}^{\prime },t\right)={\left(\frac{m_{2}\Omega_{2,GP}}{\pi }\right)}^{\frac{1}{2}}\times \\ & & \times e^{-\frac{m_{2}\Omega_{2,GP}}{2}\left\{{\left[x_{2}-x_{2}\left(t\right)\right]}^{2}+{\left[x_{2}^{\prime }-x_{2}\left(t\right)\right]}^{2}\right\}}e^{+im_{2}\dot{x_{2}}\left(t\right)\left\{\left[x_{2}-x_{2}\left(t\right)\right]-\left[x_{2}^{\prime }-x_{2}\left(t\right)\right]\right\}},\\ & & \rho_{12,MF}^{\left(2\right)}\left(x_{1},x_{1}^{\prime },x_{2},x_{2}^{\prime },t\right)=\rho_{1,MF}^{\left(1\right)}\left(x_{1},x_{1}^{\prime },t\right)\rho_{2,MF}^{\left(1\right)}\left(x_{2},x_{2}^{\prime },t\right).\; \end{array}$$

Recall that, at the mean-field level, $\rho_{12,MF}^{\left(2\right)}\left(x_{1},x_{1}^{\prime },x_{2},x_{2}^{\prime },t\right)$ is naturally built as a product of intraspecies reduced one-particle density matrices. Correspondingly, the diagonal parts of (48) are the intraspecies, $\rho_{1,MF}^{\left(1\right)}\left(x_{1},t\right)=N_{1}\rho_{1,GP}^{\left(1\right)}\left(x_{2},t\right)$, $\rho_{1,GP}^{\left(1\right)}\left(x_{1},t\right)={\left(\frac{m_{1}\Omega_{1,GP}}{\pi }\right)}^{\frac{1}{2}}\times$$\times e^{-m_{1}\Omega_{1,GP}{\left[x_{1}-x_{1}\left(t\right)\right]}^{2}}$ and $\rho_{2,MF}^{\left(1\right)}\left(x_{2},t\right)=N_{2}\rho_{2,GP}^{\left(1\right)}\left(x_{2},t\right)$, $\rho_{2,GP}^{\left(1\right)}\left(x_{2},t\right)={\left(\frac{m_{2}\Omega_{2,GP}}{\pi }\right)}^{\frac{1}{2}}\times$$\times e^{-m_{2}\Omega_{2,GP}{\left[x_{2}-x_{2}\left(t\right)\right]}^{2}}$, and interspecies, $\rho_{12,MF}^{\left(2\right)}\left(x_{1},x_{2},t\right)=\rho_{1,MF}^{\left(1\right)}\left(x_{1},t\right)\rho_{2,MF}^{\left(1\right)}\left(x_{2},t\right)$, densities.

To proceed, let $\bar{\Phi }\left(x_{1,1},\ldots ,x_{1,N_{1}},x_{2,1},\ldots ,x_{2,N_{2}},t\right)=\prod_{j=1}^{N_{1}}{\bar{\phi }}_{1}\left(x_{1,j},t\right)\prod_{k=1}^{N_{2}}{\bar{\phi }}_{2}\left(x_{2,k},t\right)$ be the periodic part of (43), i.e., $\Phi \left(x_{1,1},\ldots ,x_{1,N_{1}},x_{2,1},\ldots ,x_{2,N_{2}},t\right)=e^{-i\left(N_{1}\mu_{1,F}+N_{2}\mu_{2,F}\right)t}\times$$\times \bar{\Phi }\left(x_{1,1},\ldots ,x_{1,N_{1}},x_{2,1},\ldots ,x_{2,N_{2}},t\right)$. The combination $N_{1}\mu_{1,F}+N_{2}\mu_{2,F}$ in the phase leads to cancellation of some terms in the mean-field Floquet energy per particle (see Appendix B), which is defined by sandwiching the many-boson Floquet Hamiltonian with the mean-field quasienergy wavefunction,

(49) $$\begin{array}{ccc} & & \varepsilon_{F}^{GP}=\frac{\langle \bar{\Phi }|\hat{H}-i\frac{\partial }{\partial t}|\bar{\Phi }\rangle }{N}=\frac{1}{2}\frac{\Lambda_{12}\Omega_{1,GP}+\Lambda_{21}\Omega_{2,GP}}{\Lambda_{12}+\Lambda_{21}}-\frac{1}{4\left(\Lambda_{12}+\Lambda_{21}\right)\left(m_{1}\Lambda_{12}+m_{2}\Lambda_{21}\right)}\times \\ & & \times \left[\frac{m_{1}m_{2}\Lambda_{12}\Lambda_{21}{\left(\frac{f_{L,1}}{m_{1}}-\frac{f_{L,2}}{m_{2}}\right)}^{2}\left(\omega^{2}-\omega_{L}^{2}\right)}{{\left(\Omega_{12}^{2}-\omega_{L}^{2}\right)}^{2}}+\frac{{\left(\Lambda_{12}f_{L,1}+\Lambda_{21}f_{L,2}\right)}^{2}}{\omega^{2}-\omega_{L}^{2}}\right].\; \end{array}$$

Expression (49) is in analogy to a mixture’s ground state, in which the mean-field energy per particle is obtained by sandwiching the static many-boson Hamiltonian with the static mean-field wavefunction.

We are now in the position to examine the relations between the many-body and mean-field solutions of the driven bosonic mixture at the limit of an infinite number of particles. Thus, the following relations readily hold:

(50) limN1→∞N2→∞ρ1(1)(x1,x1′,t)N1=ρ1,GP(1)(x1,x1′,t),limN1→∞N2→∞ρ2(1)(x2,x2′,t)N2=ρ2,GP(1)(x2,x2′,t)

for the time-dependent reduced one-particle density matrices per particle, with limN1→∞N2→∞ρ1(1)(x1,t)N1=ρ1,GP(1)(x1,t), limN1→∞N2→∞ρ2(1)(x2,t)N2=ρ2,GP(1)(x2,t) for the time-dependent densities per particle, and

(51) limN1→∞N2→∞ρ12(2)(x1,x1′,x2,x2′,t)N1N2=ρ1,GP(1)(x1,x1′,t)ρ2,GP(1)(x2,x2′,t),

with limN1→∞N2→∞ρ12(2)(x1,x2,t)N1N2=ρ1,GP(1)(x1,t)ρ2,GP(1)(x2,t) for the time-dependent interspecies two-particle density.

The above relations between the time-dependent many-body and mean-field reduced density matrices generalize for time-periodic (Floquet) mixtures results existing in the literature for the ground state of trapped mixtures and, when available, results for the dynamics of interacting bosons with time-independent Hamiltonians. On top of and contrary to that, an appealing and different result emerges for the quasienergy per particle. Here, we find

(52) limN1→∞N2→∞EFN=12Λ12Ω1,GP+Λ21Ω2,GPΛ12+Λ21−14(Λ12+Λ21)(m1Λ12+m2Λ21)××m1m2Λ12Λ21fL,1m1−fL,2m22Ω122−ωL2+Λ12fL,1+Λ21fL,22ω2−ωL2≠εFGP,

implying that the quasienergy per particle computed at the many-body level of theory (33) is different from that at the mean-field level (49), even at the infinite-particle-number limit.

Comparatively, the result (52) for the quasienergy per particle of the driven mixture differs from the literature results for the ground-state energy per particle of trapped interacting bosons and mixtures at the limit of an infinite number of particles. Here, the difference can be seen as the additional factor $\frac{\omega^{2}-\omega_{L}^{2}}{\Omega_{12}^{2}-\omega_{L}^{2}}$, the ratio of the two resonance frequencies’ poles, appearing in the first term inside the square brackets of the mean-field quasienergy (49). This additional factor emerges because the non-linear interaction terms in the mean-field treatment cannot compensate both poles in the driven amplitudes $x_{1}\left(t\right)$ and $x_{2}\left(t\right)$. More precisely, the mean-field treatment renormalizes the corresponding phases only to the center-of-mass frequency $\omega^{2}$ and not also to the relative center-of-mass frequency $\Omega_{12}^{2}$ (see Appendix B for the derivation).

When are the quasienergies equal? Trivially, if there is no interaction between the two species, implying that $\Omega_{12}=\omega$ and, therefore, $\frac{\omega^{2}-\omega_{L}^{2}}{\Omega_{12}^{2}-\omega_{L}^{2}}=1$, i.e., we have then two harmonically trapped non-coupled driven Bose–Einstein condensates; otherwise, as long as the relative center-of-mass coordinate is not activated by the forces, the respective quasienergies per particle are equal, namely,

(53) limN1→∞N2→∞EFN|fL,1m1=fL,2m2=12Λ12Ω1,GP+Λ21Ω2,GPΛ12+Λ21−−fL,12(m1Λ12+m2Λ21)4m12(Λ12+Λ21)(ω2−ωL2)=εFGP|fL,1m1=fL,2m2.

The latter occurs independently of the number of particles and strengths of interactions, when the forces and masses are interrelated by $\frac{f_{L,1}}{m_{1}}=\frac{f_{L,2}}{m_{2}}$. This brings the present section on many-body and mean-field quasienergies and Floquet reduced density matrices to an end.

## 4. Angular Momentum and Its Fluctuations in a Bose–Einstein Condensate Steered by an Interacting Bosonic Impurity

So far, we considered periodically driving a mixture in one spatial dimension. A natural extension and application is to consider a two-dimensional mixture and drive it correspondingly along the *x* and *y* directions such that it is steered, namely, circularly driven. Then, angular momentum is imprinted on the bosons. We use the harmonic-interaction model for the driven mixture to investigate the roles of intraspecies and interspecies interactions and resulting angular-momentum fluctuations [72,74] at the many-body and mean-field levels. The availability of a solvable model is instructive.

Our strategy in this section is to begin with steering a single particle in two spatial dimensions and computing the angular momentum and fluctuations, namely, the expectation value of the angular-momentum operator and its variance. Then, we steer the *N*-boson problem and investigate the role of interaction at the many-body and mean-field levels of theory. Finally, we steer species 1 bosons, which serve as an interacting bosonic impurity, embedded in species 2 bosons, where the latter are not steered, and then investigate the modes of rotation, distribution of angular momentum between the species, and fluctuations.

Consider the circularly driven two-dimensional quantum harmonic oscillator [r=(x,y)],

(54) $$\hat{h}\left(r,t\right)=-\frac{1}{2m}\frac{\partial^{2}}{\partial r^{2}}+\frac{1}{2}m\omega^{2}r^{2}-f_{L}\left[x\cos\left(\omega_{L}t\right)+y\sin\left(\omega_{L}t\right)\right].$$

The ground Floquet solution of the time-dependent Schrödinger equation is given by

(55) $$\begin{array}{ccc} & & \psi \left(r,t\right)={\left(\frac{m\omega }{\pi }\right)}^{\frac{1}{2}}e^{-i2\varepsilon_{F}t}e^{-\frac{m\omega }{2}\left\{{\left[x-x(t)\right]}^{2}+{\left[y-y(t)\right]}^{2}\right\}}e^{+im\left[\dot{x}\left(t\right)x+\dot{y}\left(t\right)y\right]},\\ & & x\left(t\right)=a\cos\left(\omega_{L}t\right),\;y\left(t\right)=a\sin\left(\omega_{L}t\right),\;a=\frac{f_{L}}{m(\omega^{2}-\omega_{L}^{2})}, \end{array}$$

where $\varepsilon_{F}$ is given in (5).

The time-dependent wavefunction (55) can be seen, apart from the time-dependent phase $e^{-i2\varepsilon_{F}t}$, which is irrelevant for the properties discussed in this section, as the ground state of the two-dimensional isotropic harmonic oscillator being translated by x(t) and y(t) and boosted by $m\dot{x}\left(t\right)$ and $m\dot{y}\left(t\right)$ along the *x* and *y* directions, respectively. A useful way to evaluate the expectation value of the angular-momentum operator and its variance carried by a translated and boosted wavefunction is to use the transformation properties of the angular-momentum operator under translations and boosts. In Appendix C, we derive and discuss the corresponding expressions for the most general wavefunction treated in this work, that of driven mixtures, where each species can be translated and boosted differently.

Using (A25) for N=1 particles, we can readily evaluate the expectation value of the angular-momentum operator ${\hat{l}}_{z}=\hat{x}{\hat{p}}_{y}-\hat{y}{\hat{p}}_{x}$, which reads

(56) $$\langle {\hat{l}}_{z}\rangle {|}_{\psi (r,t)}=x\left(t\right)m\dot{y}\left(t\right)-y\left(t\right)m\dot{x}\left(t\right)=m\omega_{L}{\left[\frac{f_{L}}{m(\omega^{2}-\omega_{L}^{2})}\right]}^{2}.$$

The value (56) is that of a ‘classical’ particle encircling the origin with angular velocity $\omega_{L}$ and radius $\left|\frac{f_{L}}{m(\omega^{2}-\omega_{L}^{2})}\right|$. The radius is determined by the driving force and diverges as one approaches the resonance. On the other end, the radius is always non-zero (for $f_{L}\neq 0$), meaning that the expectation value of the angular momentum for the driven particle is non-zero (and positive).

The Floquet solution (55) of the steered harmonic oscillator is not an eigenfunction of the angular-momentum operator. Using the transformation properties of the latter under translations and boosts, one can readily compute the angular-momentum variance, which is found to depend on the position and momentum variances along the *x* and *y* directions (see Appendix C). Thus, employing (A27) for N=1 particles, we find

(57) $$\begin{array}{ccc} & & \Delta_{{\hat{l}}_{z}}^{2}{|}_{\psi (r,t)}=\left[x^{2}\left(t\right)+y^{2}\left(t\right)\right]\frac{m\omega }{2}+\left[m^{2}{\dot{x}}^{2}\left(t\right)+m^{2}{\dot{y}}^{2}\left(t\right)\right]\frac{1}{2m\omega }=\\ & & =\frac{m\left(\omega^{2}+\omega_{L}^{2}\right)}{2\omega }{\left[\frac{f_{L}}{m(\omega^{2}-\omega_{L}^{2})}\right]}^{2}. \end{array}$$

The dependence of the variance (57) on the radius $\left|\frac{f_{L}}{m(\omega^{2}-\omega_{L}^{2})}\right|$ is in conjunction with the expectation value (56). For instance, for tight confinements (ω≫1), the radius diminishes, and so does the variance. Note, however, that for ω→0, the variance diverges, whereas the expectation value of the angular momentum does not. This signifies that the angular-momentum variance depends on the size of the wavepacket, and for weaker confinements, this size increases. We will return to these points when interactions set in.

Let us move to the circularly driven isotropic harmonic-interaction model in two spatial dimensions,

(58) $$\begin{array}{ccc} & & \hat{H}\left(r_{1},\ldots ,r_{N},t\right)=\sum_{j=1}^{N}\{-\frac{1}{2m}\frac{\partial^{2}}{\partial r_{j}^{2}}+\frac{1}{2}m\omega^{2}r_{j}^{2}-\\ & & -f_{L}\left[x_{j}\cos\left(\omega_{L}t\right)+y_{j}\sin\left(\omega_{L}t\right)\right]\}+\lambda \sum_{1\leq j<k}^{N}{\left(r_{j}-r_{k}\right)}^{2}.\; \end{array}$$

The ground Floquet solution is given by

(59) $$\begin{array}{ccc} & & \Psi \left(r_{1},\ldots ,r_{N},t\right)={\left(\frac{m\Omega }{\pi }\right)}^{\frac{N-1}{2}}{\left(\frac{m\omega }{\pi }\right)}^{\frac{1}{2}}e^{-i2E_{F}t}e^{-\frac{\alpha }{2}\sum_{j=1}^{N}\left\{{\left[x_{j}-x\left(t\right)\right]}^{2}+{\left[y_{j}-y\left(t\right)\right]}^{2}\right\}}\times \\ & & \times e^{-\beta \sum_{1\leq j<k}^{N}\left\{\left[x_{j}-x\left(t\right)\right]\left[x_{k}-x\left(t\right)\right]+\left[y_{j}-y\left(t\right)\right]\left[y_{k}-y\left(t\right)\right]\right\}}e^{+im\left[\dot{x}\left(t\right)\sum_{j=1}^{N}x_{j}+\dot{y}\left(t\right)\sum_{j=1}^{N}y_{j}\right]}, \end{array}$$

where $E_{F}$ is given in (11). Now, the expectation value of the many-particle angular-momentum operator ${\hat{L}}_{Z}=\sum_{j=1}^{N}\left({\hat{x}}_{j}{\hat{p}}_{y,j}-{\hat{y}}_{j}{\hat{p}}_{x,j}\right)$ and the respective variance are given by

(60) $$\begin{array}{ccc} & & \frac{1}{N}\langle {\hat{L}}_{Z}\rangle {|}_{\Psi (r_{1},\ldots ,r_{N},t)}=m\omega_{L}{\left[\frac{f_{L}}{m(\omega^{2}-\omega_{L}^{2})}\right]}^{2},\\ & & \frac{1}{N}\Delta_{{\hat{L}}_{Z}}^{2}{|}_{\Psi (r_{1},\ldots ,r_{N},t)}=\frac{m\left(\omega^{2}+\omega_{L}^{2}\right)}{2\omega }{\left[\frac{f_{L}}{m(\omega^{2}-\omega_{L}^{2})}\right]}^{2},\; \end{array}$$

where (A25) and (A27) are employed. We see that the interaction between particles enters neither the expectation value nor the variance of the angular-momentum operator at the many-body level, and the values per particle are given by those of the steered single-particle system (see (56) and (57)).

Let us examine the respective quantities at the mean-field level, where the Floquet solution was analytically derived in Section 2 and reads in two spatial dimensions:

(61) $$\begin{array}{ccc} & & \Phi \left(r_{1},\ldots ,r_{N},t\right)={\left(\frac{m\Omega_{GP}}{\pi }\right)}^{\frac{N}{2}}e^{-i2N\mu_{F}t}e^{-\frac{m\Omega_{GP}}{2}\sum_{j=1}^{N}\left\{{\left[x_{j}-x\left(t\right)\right]}^{2}+{\left[y_{j}-y\left(t\right)\right]}^{2}\right\}}\times \\ & & \times e^{+im\left[\dot{x}\left(t\right)\sum_{j=1}^{N}x_{j}+\dot{y}\left(t\right)\sum_{j=1}^{N}y_{j}\right]}, \end{array}$$

where $\mu_{F}$ is given in (20). Computing now the expectation value and variance of ${\hat{L}}_{Z}$, we find

(62) $$\begin{array}{ccc} & & \frac{1}{N}\langle {\hat{L}}_{Z}\rangle {|}_{\Phi (r_{1},\ldots ,r_{N},t)}=m\omega_{L}{\left[\frac{f_{L}}{m(\omega^{2}-\omega_{L}^{2})}\right]}^{2},\\ & & \frac{1}{N}\Delta_{{\hat{L}}_{Z}}^{2}{|}_{\Phi (r_{1},\ldots ,r_{N},t)}=\frac{m\left(\omega_{GP}^{2}+\omega_{L}^{2}\right)}{2\omega_{GP}}{\left[\frac{f_{L}}{m(\omega^{2}-\omega_{L}^{2})}\right]}^{2}=\frac{m\left(\omega^{2}+\frac{2\Lambda }{m}+\omega_{L}^{2}\right)}{2\sqrt{\omega^{2}+\frac{2\Lambda }{m}}}{\left[\frac{f_{L}}{m(\omega^{2}-\omega_{L}^{2})}\right]}^{2},\; \end{array}$$

where the variance exhibits dependence on the interaction strength. This behavior is because the position and momentum variances (of the non-boosted/translated wavefunction Φ) themselves depend, at the mean-field level, on the interaction strength, $\frac{1}{N}\Delta_{\hat{X}}^{2}{|}_{\Phi }=\frac{1}{N}\Delta_{\hat{Y}}^{2}{|}_{\Phi }=\frac{1}{2m\sqrt{\omega^{2}+\frac{2\Lambda }{m}}}$ and $\frac{1}{N}\Delta_{{\hat{P}}_{X}}^{2}{|}_{\Phi }=\frac{1}{N}\Delta_{{\hat{P}}_{Y}}^{2}{|}_{\Phi }=\frac{m\sqrt{\omega^{2}+\frac{2\Lambda }{m}}}{2}$. In particular, for repulsive [$\Lambda \to {\left(-m\omega^{2}/2\right)}^{+}$] as well as for attractive (Λ≫1) interactions, the angular-momentum variance (62) can diverge, unlike the situation at the many-body level of theory (60). On the other end, the variance does not vanish for any interacting strength. The situation becomes more interesting when we steer species 1 bosons embedded in species 2 bosons (see below). Finally, we note that the expectation values per particle of the angular momentum at the many-body and mean-field levels of theory coincide (for finite systems and) at the limit of an infinite number of particles, as is expected for one-body operators [64,72].

Let us proceed to the scenario of a steered mixture. Consider the harmonic-interaction model in two spatial dimensions for circularly-driven species 1 bosons embedded in un-steered species 2 bosons:

(63) $$\begin{array}{ccc} & & \hat{H}\left(r_{1,1},\ldots ,r_{1,N_{1}},r_{2,1},\ldots ,r_{2,N_{2}},t\right)=\sum_{j=1}^{N_{1}}\{-\frac{1}{2m_{1}}\frac{\partial^{2}}{\partial r_{1,j}^{2}}+\frac{1}{2}m_{1}\omega^{2}r_{1,j}^{2}-\\ & & -f_{L,1}\left[x_{1,j}\cos\left(\omega_{L}t\right)+y_{1,j}\sin\left(\omega_{L}t\right)\right]\}+\lambda_{1}\sum_{1\leq j<k}^{N_{1}}{\left(r_{1,j}-r_{1,k}\right)}^{2}+\\ & & +\sum_{j=1}^{N_{2}}\left(-\frac{1}{2m_{2}}\frac{\partial^{2}}{\partial r_{2,j}^{2}}+\frac{1}{2}m_{2}\omega^{2}r_{2,j}^{2}\right)+\lambda_{2}\sum_{1\leq j<k}^{N_{2}}{\left(r_{2,j}-r_{2,k}\right)}^{2}+\lambda_{12}\sum_{j=1}^{N_{1}}\sum_{k=1}^{N_{2}}{\left(r_{1,j}-r_{2,k}\right)}^{2}.\; \end{array}$$

We expect the interspecies interaction $\lambda_{12}$ to mediate the rotation from species 1 to species 2, and the question to answer is in what way.

The ground Floquet solution to (63) is given by

(64) $$\begin{array}{ccc} & & \Psi \left(r_{1,1},\ldots ,r_{1,N_{1}},r_{2,1},\ldots ,r_{2,N_{2}},t\right)={\left(\frac{m_{1}\Omega_{1}}{\pi }\right)}^{\frac{N_{1}-1}{2}}{\left(\frac{m_{2}\Omega_{2}}{\pi }\right)}^{\frac{N_{2}-1}{2}}{\left(\frac{M_{12}\Omega_{12}}{\pi }\right)}^{\frac{1}{2}}{\left(\frac{M\omega }{\pi }\right)}^{\frac{1}{2}}\times \\ & & \times e^{-i2E_{F}t}e^{-\frac{\alpha_{1}}{2}\sum_{j=1}^{N_{1}}\left\{{\left[x_{1,j}-x_{1}\left(t\right)\right]}^{2}+{\left[y_{1,j}-y_{1}\left(t\right)\right]}^{2}\right\}-\beta_{1}\sum_{1\leq j<k}^{N_{1}}\left\{\left[x_{1,j}-x_{1}\left(t\right)\right]\left[x_{1,k}-x_{1}\left(t\right)\right]+\left[y_{1,j}-y_{1}\left(t\right)\right]\left[y_{1,k}-y_{1}\left(t\right)\right]\right\}}\times \\ & & \times e^{-\frac{\alpha_{2}}{2}\sum_{j=1}^{N_{2}}\left\{{\left[x_{2,j}-x_{2}\left(t\right)\right]}^{2}+{\left[y_{2,j}-y_{2}\left(t\right)\right]}^{2}\right\}-\beta_{2}\sum_{1\leq j<k}^{N_{2}}\left\{\left[x_{2,j}-x_{2}\left(t\right)\right]\left[x_{2,k}-x_{2}\left(t\right)\right]+\left[y_{2,j}-y_{2}\left(t\right)\right]\left[y_{2,k}-y_{2}\left(t\right)\right]\right\}}\times \\ & & \times e^{+\gamma \sum_{j=1}^{N_{1}}\sum_{k=1}^{N_{2}}\left\{\left[x_{1,j}-x_{1}\left(t\right)\right]\left[x_{2,k}-x_{2}\left(t\right)\right]+\left[y_{1,j}-y_{1}\left(t\right)\right]\left[y_{2,k}-y_{2}\left(t\right)\right]\right\}}\times \\ & & \times e^{+i\left\{m_{1}\left[{\dot{x}}_{1}\left(t\right)\sum_{j=1}^{N_{1}}x_{1,j}+{\dot{y}}_{1}\left(t\right)\sum_{j=1}^{N_{1}}y_{1,j}\right]+m_{2}\left[{\dot{x}}_{2}\left(t\right)\sum_{j=1}^{N_{2}}x_{2,j}+{\dot{y}}_{2}\left(t\right)\sum_{j=1}^{N_{2}}y_{2,j}\right]\right\}},\;\; \end{array}$$

where the quasienergy $E_{F}$ is given in (33), $x_{1}\left(t\right)=a_{1}\cos\left(\omega_{L}t\right)$, $y_{1}\left(t\right)=a_{1}\sin\left(\omega_{L}t\right)$, $x_{2}\left(t\right)=a_{2}\cos\left(\omega_{L}t\right)$, and $y_{2}\left(t\right)=a_{2}\sin\left(\omega_{L}t\right)$, and the amplitudes $a_{1}$ and $a_{2}$, determining the respective radii of rotations, are:

(65) $$\begin{array}{ccc} & & a_{1}=\frac{f_{L,1}N_{1}}{M}\left[\frac{1}{\omega^{2}-\omega_{L}^{2}}+\frac{m_{2}N_{2}}{m_{1}N_{1}}\frac{1}{\Omega_{12}^{2}-\omega_{L}^{2}}\right]=\frac{f_{L,1}\left[\omega^{2}+\frac{2\Lambda_{12}}{m_{2}}-\omega_{L}^{2}\right]}{m_{1}\left(\omega^{2}-\omega_{L}^{2}\right)\left[\omega^{2}+2\left(\frac{\Lambda_{12}}{m_{2}}+\frac{\Lambda_{21}}{m_{1}}\right)-\omega_{L}^{2}\right]},\\ & & a_{2}=\frac{f_{L,1}N_{1}}{M}\left[\frac{1}{\omega^{2}-\omega_{L}^{2}}-\frac{1}{\Omega_{12}^{2}-\omega_{L}^{2}}\right]=\frac{f_{L,1}\frac{2\Lambda_{12}}{m_{2}}}{m_{1}\left(\omega^{2}-\omega_{L}^{2}\right)\left[\omega^{2}+2\left(\frac{\Lambda_{12}}{m_{2}}+\frac{\Lambda_{21}}{m_{1}}\right)-\omega_{L}^{2}\right]}. \end{array}$$

The resulting amplitudes $a_{1}$ and $a_{2}$ deserve a discussion. Despite that only species 1 is being steered, there are contributions from two distinct poles, at $\omega^{2}$ and at $\Omega_{12}^{2}=\omega^{2}+2\left(\frac{\Lambda_{12}}{m_{2}}+\frac{\Lambda_{21}}{m_{1}}\right)$, signifying the activation of the center-of-mass $Q_{N}$ and relative center-of-mass $Q_{N-1}$ coordinates in the presence of coupling between the two species. The radius of rotation of species 2, $a_{2}$, is linear in the intraspecies interacting parameter, $\Lambda_{12}=N_{1}\lambda_{12}$, and vanishes, as expected for diminishing coupling between the steered species 1 and un-steered species 2 Bose–Einstein condensates.

It is appealing to see that there is also a zero for $a_{1}$ when

(66) $$\omega_{L}^{0}=\sqrt{\omega^{2}+\frac{2\Lambda_{12}}{m_{2}}}>0\;⟹\;a_{1}{|}_{\omega_{L}=\omega_{L}^{0}}=0,\;a_{2}{|}_{\omega_{L}=\omega_{L}^{0}}=-\frac{f_{L,1}}{2\Lambda_{21}},$$

which is ‘born’ only due to the interaction between species 1 and species 2 bosons. Physically, a vanishing value for $a_{1}$ means that species 1 does not rotate or move, despite being driven by force $f_{L,1}$ at the angular frequency $\omega_{L}^{0}$. Alternatively, we may say that the effect of the driving force $f_{L,1}$ exerted on species 1 is then completely transferred to species 2. The value of $a_{2}$ at $\omega_{L}^{0}$ (see (66)) is inversely proportional to the intraspecies interaction parameter $\Lambda_{21}=N_{2}\lambda_{12}$. Further analysis of $a_{1}$ and $a_{2}$ is given below when analyzing the distribution of angular momentum between the two species.

The mean-field Floquet wavefunction in two spatial dimensions is needed to compute the respective properties and is given by

(67) $$\begin{array}{ccc} & & \Phi \left(r_{1,1},\ldots ,r_{1,N_{1}},r_{2,1},\ldots ,r_{2,N_{2}},t\right)={\left(\frac{m_{1}\Omega_{1,GP}}{\pi }\right)}^{\frac{N_{1}}{2}}{\left(\frac{m_{2}\Omega_{2,GP}}{\pi }\right)}^{\frac{N_{2}}{2}}e^{-i2\left(N_{1}\mu_{1,F}+N_{2}\mu_{2,F}\right)t}\times \\ & & \times e^{-\frac{m_{1}\Omega_{1,GP}}{2}\sum_{j=1}^{N_{1}}\left\{{\left[x_{1,j}-x_{1}\left(t\right)\right]}^{2}+{\left[y_{1,j}-y_{1}\left(t\right)\right]}^{2}\right\}}e^{-\frac{m_{2}\Omega_{2,GP}}{2}\sum_{j=1}^{N_{2}}\left\{{\left[x_{2,j}-x_{2}\left(t\right)\right]}^{2}+{\left[y_{2,j}-y_{2}\left(t\right)\right]}^{2}\right\}}\times \\ & & \times e^{+i\left\{m_{1}\left[{\dot{x}}_{1}\left(t\right)\sum_{j=1}^{N_{1}}x_{1,j}+{\dot{y}}_{1}\left(t\right)\sum_{j=1}^{N_{1}}y_{1,j}\right]+m_{2}\left[{\dot{x}}_{2}\left(t\right)\sum_{j=1}^{N_{2}}x_{2,j}+{\dot{y}}_{2}\left(t\right)\sum_{j=1}^{N_{2}}y_{2,j}\right]\right\}},\;\; \end{array}$$

where the quasichemical potentials $\mu_{1,F}$ and $\mu_{2,F}$ are given in (46).

We are now in the position to compute the expectation value of the angular-momentum operator and its variance and to compare the many-body and mean-field results. For a mixture ${\hat{L}}_{Z}={\hat{L}}_{Z_{1}}+{\hat{L}}_{Z_{2}}$, we shall also investigate the connection between the mixture’s and individual species’ angular momenta. We start with the expectation values at the many-body level of theory, which take on the form:

(68) $$\begin{array}{ccc} & & \;\;\;\;\;\;\frac{1}{N}\langle {\hat{L}}_{Z}\rangle {|}_{\Psi (r_{1,1},\ldots ,r_{2,N_{2}},t)}=\frac{\Lambda_{12}}{\Lambda_{12}+\Lambda_{21}}\cdot \frac{1}{N_{1}}\langle {\hat{L}}_{Z_{1}}\rangle {|}_{\Psi (r_{1,1},\ldots ,r_{2,N_{2}},t)}+\frac{\Lambda_{21}}{\Lambda_{12}+\Lambda_{21}}\cdot \frac{1}{N_{2}}\langle {\hat{L}}_{Z_{2}}\rangle {|}_{\Psi (r_{1,1},\ldots ,r_{2,N_{2}},t)}=\\ & & =\frac{\Lambda_{12}^{2}f_{L,1}^{2}\omega_{L}}{\left(\Lambda_{12}+\Lambda_{21}\right)\left(m_{1}\Lambda_{12}+m_{2}\Lambda_{21}\right)}\left\{\frac{1}{{\left(\omega^{2}-\omega_{L}^{2}\right)}^{2}}+\frac{m_{2}\Lambda_{21}}{m_{1}\Lambda_{12}}\frac{1}{{\left[\omega^{2}+2\left(\frac{\Lambda_{12}}{m_{2}}+\frac{\Lambda_{21}}{m_{1}}\right)-\omega_{L}^{2}\right]}^{2}}\right\},\\ & & \frac{1}{N_{1}}\langle {\hat{L}}_{Z_{1}}\rangle |{}_{\Psi (r_{1,1},\ldots ,r_{2,N_{2}},t)}=\frac{f_{L,1}^{2}\omega_{L}{\left(\omega^{2}+\frac{2\Lambda_{12}}{m_{2}}-\omega_{L}^{2}\right)}^{2}}{m_{1}{\left(\omega^{2}-\omega_{L}^{2}\right)}^{2}{\left[\omega^{2}+2\left(\frac{\Lambda_{12}}{m_{2}}+\frac{\Lambda_{21}}{m_{1}}\right)-\omega_{L}^{2}\right]}^{2}},\\ & & \frac{1}{N_{2}}\langle {\hat{L}}_{Z_{2}}\rangle |{}_{\Psi (r_{1,1},\ldots ,r_{2,N_{2}},t)}=\frac{f_{L,1}^{2}\omega_{L}{\left(\frac{2\Lambda_{12}}{m_{2}}\right)}^{2}\frac{m_{2}}{m_{1}}}{m_{1}{\left(\omega^{2}-\omega_{L}^{2}\right)}^{2}{\left[\omega^{2}+2\left(\frac{\Lambda_{12}}{m_{2}}+\frac{\Lambda_{21}}{m_{1}}\right)-\omega_{L}^{2}\right]}^{2}}.\; \end{array}$$

The amount of angular momentum carried by each species is proportional to the square of the radius of its rotation. The poles and zeros of the radii govern the sizes of angular momenta, yet only the zeros dictate the relative distribution of angular momentum between the two species; recall that only species 1 is steered. Explicitly, the ratio of the angular momenta per particle of the two species is $\frac{\frac{1}{N_{1}}\langle {\hat{L}}_{Z_{1}}\rangle {|}_{\Psi (r_{1,1},\ldots ,r_{2,N_{2}},t)}}{\frac{1}{N_{2}}\langle {\hat{L}}_{Z_{2}}\rangle {|}_{\Psi (r_{1,1},\ldots ,r_{2,N_{2}},t)}}=\frac{m_{1}}{m_{2}}\frac{{\left(\omega^{2}+\frac{2\Lambda_{12}}{m_{2}}-\omega_{L}^{2}\right)}^{2}}{{\left(\frac{2\Lambda_{12}}{m_{2}}\right)}^{2}}$ and can vary from arbitrarily large for small interspecies interaction parameters $|\Lambda_{12}|$ to arbitrarily small due to the zero of $a_{1}$ at $\omega_{L}^{0}=\sqrt{\omega^{2}+\frac{2\Lambda_{12}}{m_{2}}}$ (see (66)). Another interesting regime occurs in the vicinity of the center-of-mass pole $\frac{1}{{\left(\omega^{2}-\omega_{L}^{2}\right)}^{2}}$ (see (68)), where the ratio of the angular momenta per particle approaches the mass ratio $\frac{m_{1}}{m_{2}}$ and is independent of other parameters. Of course, in the vicinity of any of the poles, the rotation radii increase ad infinitum.

For the mean-field expectation values, $\frac{1}{N_{1}}\langle {\hat{L}}_{Z_{1}}\rangle {|}_{\Phi (r_{1,1},\ldots ,r_{2,N_{2}},t)}$ and $\frac{1}{N_{2}}\langle {\hat{L}}_{Z_{2}}\rangle {|}_{\Phi (r_{1,1},\ldots ,r_{2,N_{2}},t)}$, and, therefore, for $\frac{1}{N}\langle {\hat{L}}_{Z}\rangle {|}_{\Phi (r_{1,1},\ldots ,r_{2,N_{2}},t)}$, we find explicitly from (67), as expected, the same results as the many-body ones (see (68)). On the other hand, the variances computed at the many-body and mean-field levels of theory are different and intricate, as we now explicitly show.

To compute the variances of ${\hat{L}}_{Z}$, ${\hat{L}}_{Z_{1}}$, and ${\hat{L}}_{Z_{2}}$ of the time-dependent mixture, we need, according to Appendix C, the respective position, momentum, and angular-momentum variances of the individual species, computed at the many-body and mean-field levels with respect to the wavefunctions Ψ and Φ without translations and boosts (see (A28) and (A30)). Using the definition of the center-of-mass and relative center-of-mass Jacobi coordinates in terms of the individual species’ center-of-mass coordinates, the final result at the many-body level reads [121]:

(69) $$\begin{array}{ccc} & & \frac{1}{N_{1}}\Delta_{{\hat{X}}_{1}}^{2}{|}_{\Psi }=\frac{1}{N_{1}}\Delta_{{\hat{Y}}_{1}}^{2}{|}_{\Psi }=\frac{\Lambda_{12}}{2(m_{1}\Lambda_{12}+m_{2}\Lambda_{21})}\left[\frac{1}{\omega }+\frac{m_{2}\Lambda_{21}}{m_{1}\Lambda_{12}}\frac{1}{\sqrt{\omega^{2}+2\left(\frac{\Lambda_{12}}{m_{2}}+\frac{\Lambda_{21}}{m_{1}}\right)}}\right],\\ & & \frac{1}{N_{2}}\Delta_{{\hat{X}}_{2}}^{2}{|}_{\Psi }=\frac{1}{N_{2}}\Delta_{{\hat{Y}}_{2}}^{2}{|}_{\Psi }=\frac{\Lambda_{21}}{2(m_{1}\Lambda_{12}+m_{2}\Lambda_{21})}\left[\frac{1}{\omega }+\frac{m_{1}\Lambda_{12}}{m_{2}\Lambda_{21}}\frac{1}{\sqrt{\omega^{2}+2\left(\frac{\Lambda_{12}}{m_{2}}+\frac{\Lambda_{21}}{m_{1}}\right)}}\right],\\ & & \frac{1}{N_{1}}\Delta_{{\hat{P}}_{X_{1}}}^{2}{|}_{\Psi }=\frac{1}{N_{1}}\Delta_{{\hat{P}}_{Y_{1}}}^{2}{|}_{\Psi }=\frac{m_{1}^{2}\Lambda_{12}}{2(m_{1}\Lambda_{12}+m_{2}\Lambda_{21})}\left[\omega +\frac{m_{2}\Lambda_{21}}{m_{1}\Lambda_{12}}\sqrt{\omega^{2}+2\left(\frac{\Lambda_{12}}{m_{2}}+\frac{\Lambda_{21}}{m_{1}}\right)}\right],\\ & & \frac{1}{N_{2}}\Delta_{{\hat{P}}_{X_{2}}}^{2}{|}_{\Psi }=\frac{1}{N_{2}}\Delta_{{\hat{P}}_{Y_{2}}}^{2}{|}_{\Psi }=\frac{m_{2}^{2}\Lambda_{21}}{2(m_{1}\Lambda_{12}+m_{2}\Lambda_{21})}\left[\omega +\frac{m_{1}\Lambda_{12}}{m_{2}\Lambda_{21}}\sqrt{\omega^{2}+2\left(\frac{\Lambda_{12}}{m_{2}}+\frac{\Lambda_{21}}{m_{1}}\right)}\right],\\ & & \Delta_{{\hat{L}}_{Z_{1}}}^{2}{|}_{\Psi }=\Delta_{{\hat{L}}_{Z_{2}}}^{2}{|}_{\Psi }=\frac{\frac{\Lambda_{12}}{m_{2}}\frac{\Lambda_{21}}{m_{1}}}{2{\left(\frac{\Lambda_{12}}{m_{2}}+\frac{\Lambda_{21}}{m_{1}}\right)}^{2}}\frac{{\left[\sqrt{\omega^{2}+2\left(\frac{\Lambda_{12}}{m_{2}}+\frac{\Lambda_{21}}{m_{1}}\right)}-\omega \right]}^{2}}{\omega \sqrt{\omega^{2}+2\left(\frac{\Lambda_{12}}{m_{2}}+\frac{\Lambda_{21}}{m_{1}}\right)}}.\; \end{array}$$

The respective quantities at the mean-field level are simpler to evaluate, since Φ is a product state, and the final result reads [121]:

(70) $$\begin{array}{ccc} & & \frac{1}{N_{1}}\Delta_{{\hat{X}}_{1}}^{2}{|}_{\Phi }=\frac{1}{N_{1}}\Delta_{{\hat{Y}}_{1}}^{2}{|}_{\Phi }=\frac{1}{2m_{1}\sqrt{\omega^{2}+\frac{2}{m_{1}}\left(\Lambda_{1}+\Lambda_{21}\right)}},\\ & & \frac{1}{N_{2}}\Delta_{{\hat{X}}_{2}}^{2}{|}_{\Phi }=\frac{1}{N_{2}}\Delta_{{\hat{Y}}_{2}}^{2}{|}_{\Phi }=\frac{1}{2m_{2}\sqrt{\omega^{2}+\frac{2}{m_{2}}\left(\Lambda_{2}+\Lambda_{12}\right)}},\\ & & \frac{1}{N_{1}}\Delta_{{\hat{P}}_{X_{1}}}^{2}{|}_{\Phi }=\frac{1}{N_{1}}\Delta_{{\hat{P}}_{Y_{1}}}^{2}{|}_{\Phi }=\frac{m_{1}}{2}\sqrt{\omega^{2}+\frac{2}{m_{1}}\left(\Lambda_{1}+\Lambda_{21}\right)},\\ & & \frac{1}{N_{2}}\Delta_{{\hat{P}}_{X_{2}}}^{2}{|}_{\Phi }=\frac{1}{N_{2}}\Delta_{{\hat{P}}_{Y_{2}}}^{2}{|}_{\Phi }=\frac{m_{2}}{2}\sqrt{\omega^{2}+\frac{2}{m_{2}}\left(\Lambda_{2}+\Lambda_{12}\right)},\\ & & \Delta_{{\hat{L}}_{Z_{1}}}^{2}{|}_{\Phi }=\Delta_{{\hat{L}}_{Z_{2}}}^{2}{|}_{\Phi }=0.\; \end{array}$$

It is worthwhile to list the main properties of and differences between the variances (69) and (70), starting with the position and momentum variances, as far as they are needed for our investigations: (i) The individual species’ variances depend only on the interspecies interaction parameters $\Lambda_{12}$ and $\Lambda_{21}$ at the many-body level, whereas at the mean-field level, they additionally depend on the intraspecies interaction parameters $\Lambda_{1}$ and $\Lambda_{2}$. (ii) For attractive interactions, the momentum variances can diverge, and for repulsive interactions, the position variances can diverge. Combining (i) and (ii), one can have situations where a position or momentum variance at the many-body level does not diverge, whereas it does diverge at the mean-field level, and vice versa, i.e., mean-field variances tend to zero, whereas many-body variances remain finite. Furthermore, for the individual species’ angular-momentum variances: (iii) There is a non-vanishing value (69) at the many-body level of theory, which solely originates from the interaction between the species, and which can diverge for both repulsive and attractive interspecies interaction. Interestingly, it is marginal in the number of particles, that is, the angular-momentum variance per particle, unlike the position and momentum variances per particle, diminishes for large particle numbers and at the limit of an infinite number. Finally, at the mean-field level of theory, the angular-momentum variance (70) is zero due to separability of Φ. We are now in the position to put the pieces together.

We find for the angular-momentum variance at the many-body level

(71a) $$\begin{array}{ccc} & & \frac{1}{N}\Delta_{{\hat{L}}_{Z}}^{2}{|}_{\Psi (r_{1,1},\ldots ,r_{2,N_{2}},t)}=\frac{\Lambda_{12}^{2}f_{L,1}^{2}}{2\left(\Lambda_{12}+\Lambda_{21}\right)\left(m_{1}\Lambda_{12}+m_{2}\Lambda_{21}\right)}\times \\ & & \times \left\{\frac{\omega^{2}+\omega_{L}^{2}}{\omega {\left(\omega^{2}-\omega_{L}^{2}\right)}^{2}}+\frac{m_{2}\Lambda_{21}}{m_{1}\Lambda_{12}}\frac{\omega^{2}+2\left(\frac{\Lambda_{12}}{m_{2}}+\frac{\Lambda_{21}}{m_{1}}\right)+\omega_{L}^{2}}{\sqrt{\omega^{2}+2\left(\frac{\Lambda_{12}}{m_{2}}+\frac{\Lambda_{21}}{m_{1}}\right)}{\left[\omega^{2}+2\left(\frac{\Lambda_{12}}{m_{2}}+\frac{\Lambda_{21}}{m_{1}}\right)-\omega_{L}^{2}\right]}^{2}}\right\}, \end{array}$$

where the individual species’ values are

(71b) $$\begin{array}{ccc} & & \frac{1}{N_{1}}\Delta_{{\hat{L}}_{Z_{1}}}^{2}{|}_{\Psi (r_{1,1},\ldots ,r_{2,N_{2}},t)}=\frac{1}{N_{1}}\cdot \frac{\frac{\Lambda_{12}}{m_{2}}\frac{\Lambda_{21}}{m_{1}}}{2{\left(\frac{\Lambda_{12}}{m_{2}}+\frac{\Lambda_{21}}{m_{1}}\right)}^{2}}\frac{{\left[\sqrt{\omega^{2}+2\left(\frac{\Lambda_{12}}{m_{2}}+\frac{\Lambda_{21}}{m_{1}}\right)}-\omega \right]}^{2}}{\omega \sqrt{\omega^{2}+2\left(\frac{\Lambda_{12}}{m_{2}}+\frac{\Lambda_{21}}{m_{1}}\right)}}+\\ & & +\frac{f_{L,1}^{2}{\left[\omega^{2}+\frac{2\Lambda_{12}}{m_{2}}-\omega_{L}^{2}\right]}^{2}}{m_{1}{\left(\omega^{2}-\omega_{L}^{2}\right)}^{2}{\left[\omega^{2}+2\left(\frac{\Lambda_{12}}{m_{2}}+\frac{\Lambda_{21}}{m_{1}}\right)-\omega_{L}^{2}\right]}^{2}}\times \\ & & \times \frac{m_{1}\Lambda_{12}}{2(m_{1}\Lambda_{12}+m_{2}\Lambda_{21})}\left[\frac{\omega^{2}+\omega_{L}^{2}}{\omega }+\frac{m_{2}\Lambda_{21}}{m_{1}\Lambda_{12}}\frac{\omega^{2}+2\left(\frac{\Lambda_{12}}{m_{2}}+\frac{\Lambda_{21}}{m_{1}}\right)+\omega_{L}^{2}}{\sqrt{\omega^{2}+2\left(\frac{\Lambda_{12}}{m_{2}}+\frac{\Lambda_{21}}{m_{1}}\right)}}\right],\\ & & \frac{1}{N_{2}}\Delta_{{\hat{L}}_{Z_{2}}}^{2}{|}_{\Psi (r_{1,1},\ldots ,r_{2,N_{2}},t)}=\frac{1}{N_{2}}\cdot \frac{\frac{\Lambda_{12}}{m_{2}}\frac{\Lambda_{21}}{m_{1}}}{2{\left(\frac{\Lambda_{12}}{m_{2}}+\frac{\Lambda_{21}}{m_{1}}\right)}^{2}}\frac{{\left[\sqrt{\omega^{2}+2\left(\frac{\Lambda_{12}}{m_{2}}+\frac{\Lambda_{21}}{m_{1}}\right)}-\omega \right]}^{2}}{\omega \sqrt{\omega^{2}+2\left(\frac{\Lambda_{12}}{m_{2}}+\frac{\Lambda_{21}}{m_{1}}\right)}}+\\ & & +\frac{f_{L,1}^{2}{\left(\frac{2\Lambda_{12}}{m_{2}}\right)}^{2}\frac{m_{2}}{m_{1}}}{m_{1}{\left(\omega^{2}-\omega_{L}^{2}\right)}^{2}{\left[\omega^{2}+2\left(\frac{\Lambda_{12}}{m_{2}}+\frac{\Lambda_{21}}{m_{1}}\right)-\omega_{L}^{2}\right]}^{2}}\times \\ & & \times \frac{m_{2}\Lambda_{21}}{2(m_{1}\Lambda_{12}+m_{2}\Lambda_{21})}\left[\frac{\omega^{2}+\omega_{L}^{2}}{\omega }+\frac{m_{1}\Lambda_{12}}{m_{2}\Lambda_{21}}\frac{\omega^{2}+2\left(\frac{\Lambda_{12}}{m_{2}}+\frac{\Lambda_{21}}{m_{1}}\right)+\omega_{L}^{2}}{\sqrt{\omega^{2}+2\left(\frac{\Lambda_{12}}{m_{2}}+\frac{\Lambda_{21}}{m_{1}}\right)}}\right].\; \end{array}$$

Examining the expressions (71) for the angular-momentum variances, the first point to note is that the individual species’ variances (71b) do not sum up to the mixture’s variance (71a). This is due to the position $\langle \Psi |{\hat{X}}_{1}{\hat{X}}_{2}|\Psi \rangle =\langle \Psi |{\hat{Y}}_{1}{\hat{Y}}_{2}|\Psi \rangle$ and momentum $\langle \Psi |{\hat{P}}_{X_{1}}{\hat{P}}_{X_{2}}|\Psi \rangle =\langle \Psi |{\hat{P}}_{Y_{1}}{\hat{P}}_{Y_{2}}|\Psi \rangle$ cross-terms appearing in (A30). For the individual species (see (A28)), one of course does not have contributions from any cross-term. On the other hand, for the mixture’s angular-momentum variance, the angular-momentum cross-term $\langle \Psi |{\hat{L}}_{Z_{1}}{\hat{L}}_{Z_{2}}|\Psi \rangle$ cancels the individual species’ contributions $\langle \Psi |{\hat{L}}_{Z_{1}}^{2}|\Psi \rangle$ and $\langle \Psi |{\hat{L}}_{Z_{2}}^{2}|\Psi \rangle$ due to conservation of angular momentum, $\left({\hat{L}}_{Z_{1}}+{\hat{L}}_{Z_{2}}\right)|\Psi \rangle =0$, and hence, this cross-term does not appear in (A30). All in all, the angular-momentum variance of the driven mixture (71a) does not vanish, depends on the interspecies interaction parameters $\Lambda_{12}$ and $\Lambda_{21}$ only, can diverge either due to repulsive or attractive interspecies interaction, and is governed by the poles of the center-of-mass and relative center-of-mass coordinates.

To proceed, the angular-momentum variances at the mean-field level of theory are evaluated and read:

(72) $$\begin{array}{ccc} & & \frac{1}{N}\Delta_{{\hat{L}}_{Z}}^{2}{|}_{\Phi (r_{1,1},\ldots ,r_{2,N_{2}},t)}=\frac{\Lambda_{12}}{\Lambda_{12}+\Lambda_{21}}\cdot \frac{1}{N_{1}}\Delta_{{\hat{L}}_{Z_{1}}}^{2}{|}_{\Phi (r_{1,1},\ldots ,r_{2,N_{2}},t)}+\frac{\Lambda_{21}}{\Lambda_{12}+\Lambda_{21}}\cdot \frac{1}{N_{2}}\Delta_{{\hat{L}}_{Z_{2}}}^{2}{|}_{\Phi (r_{1,1},\ldots ,r_{2,N_{2}},t)},\\ & & \frac{1}{N_{1}}\Delta_{{\hat{L}}_{Z_{1}}}^{2}{|}_{\Phi (r_{1,1},\ldots ,r_{2,N_{2}},t)}=\frac{f_{L,1}^{2}{\left[\omega^{2}+\frac{2\Lambda_{12}}{m_{2}}-\omega_{L}^{2}\right]}^{2}}{m_{1}{\left(\omega^{2}-\omega_{L}^{2}\right)}^{2}{\left[\omega^{2}+2\left(\frac{\Lambda_{12}}{m_{2}}+\frac{\Lambda_{21}}{m_{1}}\right)-\omega_{L}^{2}\right]}^{2}}\frac{\omega^{2}+\frac{2}{m_{1}}\left(\Lambda_{1}+\Lambda_{21}\right)+\omega_{L}^{2}}{\sqrt{\omega^{2}+\frac{2}{m_{1}}\left(\Lambda_{1}+\Lambda_{21}\right)}},\\ & & \frac{1}{N_{2}}\Delta_{{\hat{L}}_{Z_{2}}}^{2}{|}_{\Phi (r_{1,1},\ldots ,r_{2,N_{2}},t)}=\frac{f_{L,1}^{2}{\left(\frac{2\Lambda_{12}}{m_{2}}\right)}^{2}\frac{m_{2}}{m_{1}}}{m_{1}{\left(\omega^{2}-\omega_{L}^{2}\right)}^{2}{\left[\omega^{2}+2\left(\frac{\Lambda_{12}}{m_{2}}+\frac{\Lambda_{21}}{m_{1}}\right)-\omega_{L}^{2}\right]}^{2}}\frac{\omega^{2}+\frac{2}{m_{2}}\left(\Lambda_{2}+\Lambda_{12}\right)+\omega_{L}^{2}}{\sqrt{\omega^{2}+\frac{2}{m_{2}}\left(\Lambda_{2}+\Lambda_{12}\right)}}, \end{array}$$

where (A28) and (A30) are employed. At the mean-field level, in contrast with the outcome (71) at the many-body level, the individual species’ angular-momentum variances add up to the mixture’s angular-momentum variance, since all cross-terms $\langle \Phi |{\hat{X}}_{1}{\hat{X}}_{2}|\Phi \rangle$, $\langle \Phi |{\hat{Y}}_{1}{\hat{Y}}_{2}|\Phi \rangle$, $\langle \Phi |{\hat{P}}_{X_{1}}{\hat{P}}_{X_{2}}|\Phi \rangle$, $\langle \Phi |{\hat{P}}_{Y_{1}}{\hat{P}}_{Y_{2}}|\Phi \rangle$, and $\langle \Phi |{\hat{L}}_{Z_{1}}{\hat{L}}_{Z_{2}}|\Phi \rangle$ vanish. On top of that, the mean-field quantities depend on all interaction parameters, intraspecies and interspecies, not only on the latter ones. Altogether, the angular-momentum variances per particle at the mean-field level (72) are different from those at the many-body level (71) for finite systems as well as at the limit of an infinite number of particles where each of the species is 100% condensed, i.e., limN1→∞N2→∞1NΔL^Z2|Ψ(r1,1,…,r2,N2,t)≠limN1→∞N2→∞1NΔL^Z2|Φ(r1,1,…,r2,N2,t) for the mixture’s angular-momentum variance and limN1→∞N2→∞1N1ΔL^Z12|Ψ(r1,1,…,r2,N2,t)≠limN1→∞N2→∞1N1ΔL^Z12|Φ(r1,1,…,r2,N2,t), limN1→∞N2→∞1N2ΔL^Z22|Ψ(r1,1,…,r2,N2,t)≠limN1→∞N2→∞1N2ΔL^Z22|Φ(r1,1,…,r2,N2,t) for the individual species’ angular-momentum variances.

A final point: What happens to the angular-momentum expectation values and variances at the frequency $\omega_{L}=\omega_{L}^{0}$ when the effect of the steering force exerted on species 1 is completely transferred to species 2? First, since $a_{1}{|}_{\omega_{L}=\omega_{L}^{0}}=0$, the expectation value of the mixture’s angular momentum and its fluctuations at the many-body and mean-field levels are now given by the respective expressions of the un-steered species 2 only. Second, one gets for the angular-momentum variance of species 1 that the only contribution at the many-body level is the marginal part, which diminishes to zero, i.e., to the mean-field value, as the number of particles increases. Third, the respective many-body and mean-field expressions for the mixture’s and species 2 variances remain different, even at the limit of an infinite number of particles when both species are 100% condensed. This wraps up the present work.

## 5. Summary and Outlook

We have presented in this work a solvable time-dependent model of a many-particle system and used it to amalgamate together several themes: (i) Mixtures of trapped Bose–Einstein condensates; (ii) Periodically driven many-particle systems; (iii) Many-body versus mean-field theory of Floquet bosonic systems at the limit of an infinite number of particles; (iv) The imprinting and fluctuations of angular momentum when a Bose–Einstein condensate is steered by interacting bosons consisting of a different species.

We introduced the driven harmonic-interaction model for mixtures, namely, $N_{1}$ bosons of mass $m_{1}$ interacting by harmonic interparticle interaction of strength $\lambda_{1}$ and driven by a time-periodic force of amplitude $f_{L,1}$ and frequency $\omega_{L}$, and $N_{2}$ bosons of mass $m_{2}$ interacting by harmonic interparticle interaction of strength $\lambda_{2}$ and driven by a time-periodic force of amplitude $f_{L,1}$ and the same frequency $\omega_{L}$. All bosons are trapped in a harmonic potential of frequency ω, and, furthermore, bosons of species 1 and species 2 interact with each other by harmonic interparticle interaction of strength $\lambda_{12}$. The model generalizes the harmonic-interaction model for mixtures to the time-dependent domain.

Using Jacobi coordinates, the time-dependent model is diagonalized, and governed by two time-dependent many-particle oscillators, which are associated with the center of mass and relative center of mass of the driven mixture, as well as two poles of respective frequencies. The frequency of the center-of-mass motion does not depend on any interaction strength, whereas the frequency of the relative center-of-mass motion depends on the interspecies interaction strength. We concentrated in this work on the ground Floquet solution of the driven mixture, and began by computing its quasienergy, the eigenvalue of the time-periodic many-particle Floquet Hamiltonian, as a function of the above parameters.

Back in the laboratory frame, all Cartesian coordinates in the time-dependent many-particle quasienergy state are coupled, and the coordinates of each species oscillate with a different amplitude that combines the two poles associated with the center-of-mass and relative center-of-mass motions. This structure of the time-dependent many-boson wavefunction allows one to explicitly obtain the intraspecies and interspecies time-dependent reduced one-particle and two-particle density matrices of the driven mixture, which possess the same coherence properties as the corresponding static quantities computed for the ground state of the trapped mixture.

One of the main goals of the present work is to ask and answer questions on the relation between many-body and mean-field theories of (trapped) interacting bosons. These have been dealt with in the literature for the ground state or for the dynamics with time-independent Hamiltonians, and we would like to extend the topic to the realm of Floquet many-boson systems. To discuss properties of the driven mixture at the limit of an infinite number of particles—specifically, the connection between the respective many-body and mean-field results for the time-dependent reduced density matrices, densities, the quasienergies that are unique for Floquet systems, as well as the variances—the solution of the coupled Floquet non-linear Schrödinger equations of the driven mixture has been explicitly derived.

With closed-form expressions at the many-body and mean-field levels of theory, we have proven explicitly that, at the limit of an infinite number of particles, the intraspecies time-dependent reduced one-particle and two-particle density matrices per particle are 100% condensed and given by the respective mean-field quantities. Furthermore, in this limit, we have proven that the interspecies time-dependent reduced two-particle density matrix per particle is separable, i.e., given by the product of the time-dependent reduced one-particle density matrices per particle of species 1 and species 2 bosons, and is given by the respective mean-field quantity. These results generalize the literature results for the ground state and the dynamics with time-independent Hamiltonians to a Floquet bosonic system.

We have found that the relation between the many-body and mean-field quasienergies per particle is intriguing, namely, that the many-body and mean-field results need not coincide. This is unlike the situation for the many-body and mean-field energies per particle of the ground state of trapped bosons and mixtures. Specifically, the coupled non-linear Schrödinger equations cannot appropriately renormalize the intensity of both poles of the oscillating mixture in the time-dependent mean-field wavefunction, and hence, in the mean-field quasienergy, in comparison with the many-body treatment. Thus, the many-body quasienergy per particle coincides with the mean-field value at the infinite-particle-number limit in the specific case where only the center-of-mass motion of the driven mixture is activated by the driving forces. In the general case when the relative center-of-mass motion is also activated, the many-body quasienergy per particle differs from the mean-field one even at the limit of an infinite number of particles.

We then investigated an application in two spatial dimensions, in which only species 1 bosons are circularly driven by a time-periodic force, and through their interspecies interaction with the un-steered species 2 bosons, induce rotation and imprint angular momentum on the latter. The dynamics are solved analytically and analyzed within many-body and mean-field theories, as presented above. We first concentrated on the average of the angular-momentum operators of each of the species. Despite steering species 1 bosons only, we show that the ratio of the angular momenta per particle of species 1 and species 2 bosons can be marginally small or arbitrarily large because of the interspecies interaction. Explicitly, the latter induces a zero in the radius of rotation of species 1 bosons at a frequency just between the center-of-mass and relative center-of-mass resonances. We have also shown that the expectation values per particle of the angular-momentum operators for the many-body and mean-field solutions of the time-periodic mixture coincide (for finite systems and) at the limit of an infinite number of particles.

Finally, we investigated the variances of the angular-momentum operators of each of the species and of the mixture. To this end, it is useful to express the Floquet quasienergy state of the steered mixture using translation and boost operators along the *x* and *y* directions. Consequently, we can express the angular-momentum variance computed from a translated and boosted wavefunction using the position, momentum, and angular-momentum variances of the un-translated/boosted wavefunction. The angular-momentum variances of species 1 and species 2 bosons as well as of the mixture were computed explicitly and compared at the many-body and mean-field levels of theory. The mean-field variances depend on the resonances and all interactions, and can diverge due to either the attractive or repulsive intraspecies and interspecies interactions. Interestingly, at the mean-field level of theory, the individual species’ angular-momentum variances are additive and sum up to the mixture’s angular-momentum variance. On the other hand, the many-body quantities depend on the resonances and interspecies interaction only, can diverge due to either the attractive or repulsive interspecies interaction, and are non-additive because of the correlation term in the time-dependent many-boson wavefunction. These differences persist at the limit of an infinite number of particles, where each of the species comprising the Floquet time-dependent mixture is 100% condensed.

Last but not least, a brief outlook: There are several directions worth pursuing of which we list four. We have discussed, in the present work, the driving of a bosonic mixture by a monochromatic force, and can envision that using a polychromatic force would be instructive. We have studied the driving of structureless bosons, and foresee that solvable models for driving structured targets embedded in a Bose–Einstein condensate would be interesting. We have investigated the imprinting of angular-momentum in a steered two-species mixture in two spatial dimensions, and suggest that exploring three-species mixtures would be valuable. Finally, the present model can serve to benchmark numerical algorithms developed to compute real-space many-boson Floquet states, which would exhibit intriguing and both fundamental and applicative facets.

## Appendix A. Time-Dependent Intraspecies Two-Particle Reduced Density Matrices and Their Limit at an Infinite Number of Particles

In this appendix, we explicitly describe the many-body and mean-field time-dependent intraspecies two-particle reduced density matrices and establish their relation at the limit of an infinite number of particles. All wavefunctions appearing below are normalized to unity. We start from the single-species driven harmonic-interaction model and proceed to the harmonic-interaction model for driven mixtures.

The time-dependent two-particle reduced density matrix is defined as $\rho^{\left(2\right)}\left(x,x^{\prime },x^{\prime \prime },x^{\prime \prime \prime },t\right)=N\left(N-1\right)\int dx_{3}\cdots dx_{N}\Psi \left(x,x^{\prime },x_{3},\ldots ,x_{N},t\right)\Psi^{*}\left(x^{\prime \prime },x^{\prime \prime \prime },x_{3},\ldots ,x_{N},t\right)$ and given for the ground Floquet solution of the driven harmonic-interaction model (13) by

(A1) $$\begin{array}{ccc} & & \rho^{\left(2\right)}\left(x,x^{\prime },x^{\prime \prime },x^{\prime \prime \prime },t\right)=N\left(N-1\right){\left(\frac{\alpha +C_{1}}{\pi }\right)}^{\frac{1}{2}}{\left(\frac{\alpha +C_{2}}{\pi }\right)}^{\frac{1}{2}}\times \\ & & \times e^{-\frac{\alpha }{2}\left\{{\left[x-x(t)\right]}^{2}+{\left[x^{\prime }-x\left(t\right)\right]}^{2}+{\left[x^{\prime \prime }-x\left(t\right)\right]}^{2}+{\left[x^{\prime \prime \prime }-x\left(t\right)\right]}^{2}\right\}}\times \\ & & \times e^{-\beta \left\{\left[x-x(t)\right]\left[x^{\prime }-x\left(t\right)\right]+\left[x^{\prime \prime }-x\left(t\right)\right]\left[x^{\prime \prime \prime }-x\left(t\right)\right]\right\}}\times \\ & & \times e^{-\frac{1}{4}C_{2}{\left\{\left[x-x(t)\right]+\left[x^{\prime }-x\left(t\right)\right]+\left[x^{\prime \prime }-x\left(t\right)\right]+\left[x^{\prime \prime \prime }-x\left(t\right)\right]\right\}}^{2}}\times \\ & & \times e^{+im\dot{x}\left(t\right)\left\{\left[x-x(t)\right]+\left[x^{\prime }-x\left(t\right)\right]-\left[x^{\prime \prime }-x\left(t\right)\right]-\left[x^{\prime \prime \prime }-x\left(t\right)\right]\right\}},\;C_{2}=-\beta^{2}\frac{N-2}{(\alpha -\beta )+(N-2)\beta }, \end{array}$$

where $C_{1}$ can be read from (15). The diagonal of (A1) is the time-dependent two-particle density and reads

(A2) $$\begin{array}{ccc} & & \rho^{\left(2\right)}\left(x,x^{\prime },t\right)=N\left(N-1\right){\left(\frac{\alpha +C_{1}}{\pi }\right)}^{\frac{1}{2}}{\left(\frac{\alpha +C_{2}}{\pi }\right)}^{\frac{1}{2}}\times \\ & & \times e^{-\left(\alpha +C_{2}\right)\left\{{\left[x-x(t)\right]}^{2}+{\left[x^{\prime }-x\left(t\right)\right]}^{2}\right\}}e^{-2\left(\beta +C_{2}\right)\left[x-x(t)\right]\left[x^{\prime }-x\left(t\right)\right]}=\\ & & =N\left(N-1\right){\left(\frac{\alpha +C_{1}}{\pi }\right)}^{\frac{1}{2}}{\left(\frac{\alpha +C_{2}}{\pi }\right)}^{\frac{1}{2}}\times \\ & & \times e^{-\left(\alpha +C_{2}\right){\left[x-\frac{f_{L}}{m(\omega^{2}-\omega_{L}^{2})}\cos\left(\omega_{L}t\right)\right]}^{2}}e^{-\left(\alpha +C_{2}\right){\left[x^{\prime }-\frac{f_{L}}{m(\omega^{2}-\omega_{L}^{2})}\cos\left(\omega_{L}t\right)\right]}^{2}}\times \\ & & \times e^{-2\left(\beta +C_{2}\right)\left[x-\frac{f_{L}}{m(\omega^{2}-\omega_{L}^{2})}\cos\left(\omega_{L}t\right)\right]\left[x^{\prime }-\frac{f_{L}}{m(\omega^{2}-\omega_{L}^{2})}\cos\left(\omega_{L}t\right)\right]}. \end{array}$$

From the mean-field wavefunction (17), the reduced two-particle density matrix at the mean-field level reads

(A3) $$\rho_{MF}^{\left(2\right)}\left(x,x^{\prime },x^{\prime \prime },x^{\prime \prime \prime },t\right)=N\left(N-1\right)\rho_{GP}^{\left(1\right)}\left(x,x^{\prime \prime },t\right)\rho_{GP}^{\left(1\right)}\left(x^{\prime },x^{\prime \prime \prime },t\right),$$

where $\rho_{GP}^{\left(1\right)}\left(x,x^{\prime },t\right)={\left(\frac{m\Omega_{GP}}{\pi }\right)}^{\frac{1}{2}}e^{-\frac{m\Omega_{GP}}{2}\left\{{\left[x-x(t)\right]}^{2}+{\left[x^{\prime }-x\left(t\right)\right]}^{2}\right\}}e^{+im\dot{x}\left(t\right)\left\{\left[x-x(t)\right]-\left[x^{\prime }-x\left(t\right)\right]\right\}}$ [Equation (22)].

The two-particle mean-field density reads $\rho_{MF}^{\left(2\right)}\left(x,x^{\prime },t\right)=N\left(N-1\right)\rho_{GP}^{\left(1\right)}\left(x,t\right)\rho_{GP}^{\left(1\right)}\left(x^{\prime },t\right)$, where $\rho_{GP}^{\left(1\right)}\left(x,t\right)={\left(\frac{m\Omega_{GP}}{\pi }\right)}^{\frac{1}{2}}e^{-m\Omega_{GP}{\left[x-x(t)\right]}^{2}}$.

Thus, at the limit of an infinite number of particles, one finds that

(A4) $$\lim_{N\to \infty }\frac{\rho^{\left(2\right)}\left(x,x^{\prime },x^{\prime \prime },x^{\prime \prime \prime },t\right)}{N(N-1)}=\rho_{GP}^{\left(1\right)}\left(x,x^{\prime \prime },t\right)\rho_{GP}^{\left(1\right)}\left(x^{\prime },x^{\prime \prime \prime },t\right)$$

for the time-dependent reduced two-particle density matrix per particle and $\lim_{N\to \infty }\frac{\rho^{\left(2\right)}\left(x,x^{\prime },t\right)}{N(N-1)}=\rho_{GP}^{\left(1\right)}\left(x,t\right)\rho_{GP}^{\left(1\right)}\left(x^{\prime },t\right)$ for the time-dependent two-particle density per particle.

For the driven mixture, we have for the ground Floquet solution (35,36) the following closed-form expressions:

(A5) $$\begin{array}{ccc} & & \rho_{1}^{\left(2\right)}\left(x_{1},x_{1}^{\prime },x_{1}^{\prime \prime },x_{1}^{\prime \prime \prime },t\right)=N_{1}\left(N_{1}-1\right){\left(\frac{\alpha_{1}+C_{1,0}}{\pi }\right)}^{\frac{1}{2}}{\left(\frac{\alpha_{1}+C_{2,0}}{\pi }\right)}^{\frac{1}{2}}\times \\ & & \times e^{-\frac{\alpha_{1}}{2}\left\{{\left[x_{1}-x_{1}\left(t\right)\right]}^{2}+{\left[x_{1}^{\prime }-x_{1}\left(t\right)\right]}^{2}+{\left[x_{1}^{\prime \prime }-x_{1}\left(t\right)\right]}^{2}+{\left[x_{1}^{\prime \prime \prime }-x_{1}\left(t\right)\right]}^{2}\right\}}\times \\ & & \times e^{-\beta_{1}\left\{\left[x_{1}-x_{1}\left(t\right)\right]\left[x_{1}^{\prime }-x_{1}\left(t\right)\right]+\left[x_{1}^{\prime \prime }-x_{1}\left(t\right)\right]\left[x_{1}^{\prime \prime \prime }-x_{1}\left(t\right)\right]\right\}}\times \\ & & \times e^{-\frac{1}{4}C_{2,0}{\left\{\left[x_{1}-x_{1}\left(t\right)\right]+\left[x_{1}^{\prime }-x_{1}\left(t\right)\right]+\left[x_{1}^{\prime \prime }-x_{1}\left(t\right)\right]+\left[x_{1}^{\prime \prime \prime }-x_{1}\left(t\right)\right]\right\}}^{2}}\times \\ & & \times e^{+im_{1}{\dot{x}}_{1}\left(t\right)\left\{\left[x_{1}-x_{1}\left(t\right)\right]+\left[x_{1}^{\prime }-x_{1}\left(t\right)\right]-\left[x_{1}^{\prime \prime }-x_{1}\left(t\right)\right]-\left[x_{1}^{\prime \prime \prime }-x_{1}\left(t\right)\right]\right\}},\\ & & \;C_{2,0}=\frac{\left(\alpha_{1}-\beta_{1}\right)C_{N_{1},0}-\left(N_{1}-2\right)\left(C_{N_{1},0}+\beta_{1}\right)\beta_{1}}{\left(\alpha_{1}-\beta_{1}\right)+\left(N_{1}-2\right)\left(C_{N_{1},0}+\beta_{1}\right)}\; \end{array}$$

and

(A6) $$\begin{array}{ccc} & & \rho_{2}^{\left(2\right)}\left(x_{2},x_{2}^{\prime },x_{2}^{\prime \prime },x_{2}^{\prime \prime \prime },t\right)=N_{2}\left(N_{2}-1\right){\left(\frac{\alpha_{2}+C_{0,1}^{\prime }}{\pi }\right)}^{\frac{1}{2}}{\left(\frac{\alpha_{2}+C_{0,2}^{\prime }}{\pi }\right)}^{\frac{1}{2}}\times \\ & & \times e^{-\frac{\alpha_{2}}{2}\left\{{\left[x_{2}-x_{2}\left(t\right)\right]}^{2}+{\left[x_{2}^{\prime }-x_{2}\left(t\right)\right]}^{2}+{\left[x_{2}^{\prime \prime }-x_{2}\left(t\right)\right]}^{2}+{\left[x_{2}^{\prime \prime \prime }-x_{2}\left(t\right)\right]}^{2}\right\}}\times \\ & & \times e^{-\beta_{2}\left\{\left[x_{2}-x_{2}\left(t\right)\right]\left[x_{2}^{\prime }-x_{2}\left(t\right)\right]+\left[x_{2}^{\prime \prime }-x_{2}\left(t\right)\right]\left[x_{2}^{\prime \prime \prime }-x_{2}\left(t\right)\right]\right\}}\times \\ & & \times e^{-\frac{1}{4}C_{0,2}^{\prime }{\left\{\left[x_{2}-x_{2}\left(t\right)\right]+\left[x_{2}^{\prime }-x_{2}\left(t\right)\right]+\left[x_{2}^{\prime \prime }-x_{2}\left(t\right)\right]+\left[x_{2}^{\prime \prime \prime }-x_{2}\left(t\right)\right]\right\}}^{2}}\times \\ & & \times e^{+im_{2}{\dot{x}}_{2}\left(t\right)\left\{\left[x_{2}-x_{2}\left(t\right)\right]+\left[x_{2}^{\prime }-x_{2}\left(t\right)\right]-\left[x_{2}^{\prime \prime }-x_{2}\left(t\right)\right]-\left[x_{2}^{\prime \prime \prime }-x_{2}\left(t\right)\right]\right\}},\\ & & \;C_{0,2}^{\prime }=\frac{\left(\alpha_{2}-\beta_{2}\right)C_{0,N_{2}}^{\prime }-\left(N_{2}-2\right)\left(C_{0,N_{2}}^{\prime }+\beta_{2}\right)\beta_{2}}{\left(\alpha_{2}-\beta_{2}\right)+\left(N_{2}-2\right)\left(C_{0,N_{2}}^{\prime }+\beta_{2}\right)}, \end{array}$$

where the time-dependent two-body densities are given explicitly by

(A7) $$\begin{array}{ccc} & & \;\;\;\;\;\;\;\;\;\;\;\;\;\;\;\rho_{1}^{\left(2\right)}\left(x_{1},x_{1}^{\prime },t\right)=N_{1}\left(N_{1}-1\right){\left(\frac{\alpha_{1}+C_{1,0}}{\pi }\right)}^{\frac{1}{2}}{\left(\frac{\alpha_{1}+C_{2,0}}{\pi }\right)}^{\frac{1}{2}}\times \\ & & \;\;\;\;\;\;\;\;\;\;\;\;\;\;\;\times e^{-\left(\alpha_{1}+C_{2,0}\right)\left\{{\left[x_{1}-x_{1}\left(t\right)\right]}^{2}+{\left[x_{1}^{\prime }-x_{1}\left(t\right)\right]}^{2}\right\}}e^{-2\left(\beta_{1}+C_{2,0}\right)\left[x_{1}-x_{1}\left(t\right)\right]\left[x_{1}^{\prime }-x_{1}\left(t\right)\right]}=\\ & & \;\;\;\;\;\;\;\;\;\;\;\;\;\;\;=N_{1}\left(N_{1}-1\right){\left(\frac{\alpha_{1}+C_{1,0}}{\pi }\right)}^{\frac{1}{2}}{\left(\frac{\alpha_{1}+C_{2,0}}{\pi }\right)}^{\frac{1}{2}}\times \\ & & \;\;\;\;\;\;\;\;\;\;\;\;\;\;\;\times e^{-\left(\alpha_{1}+C_{2,0}\right){\left\{x_{1}-\frac{1}{M}\left[\frac{N_{1}f_{L,1}+N_{2}f_{L,2}}{\omega^{2}-\omega_{L}^{2}}+\frac{m_{2}N_{2}\left(\frac{f_{L,1}}{m_{1}}-\frac{f_{L,2}}{m_{2}}\right)}{\Omega_{12}^{2}-\omega_{L}^{2}}\right]\cos\left(\omega_{L}t\right)\right\}}^{2}}\times \\ & & \;\;\;\;\;\;\;\;\;\;\;\;\;\;\;\times e^{-\left(\alpha_{1}+C_{2,0}\right){\left\{x_{1}^{\prime }-\frac{1}{M}\left[\frac{N_{1}f_{L,1}+N_{2}f_{L,2}}{\omega^{2}-\omega_{L}^{2}}+\frac{m_{2}N_{2}\left(\frac{f_{L,1}}{m_{1}}-\frac{f_{L,2}}{m_{2}}\right)}{\Omega_{12}^{2}-\omega_{L}^{2}}\right]\cos\left(\omega_{L}t\right)\right\}}^{2}}\times \\ & & \;\;\;\;\;\;\;\;\;\;\;\;\;\;\;\times e^{-2\left(\beta_{1}+C_{2,0}\right)\left\{x_{1}-\frac{1}{M}\left[\frac{N_{1}f_{L,1}+N_{2}f_{L,2}}{\omega^{2}-\omega_{L}^{2}}+\frac{m_{2}N_{2}\left(\frac{f_{L,1}}{m_{1}}-\frac{f_{L,2}}{m_{2}}\right)}{\Omega_{12}^{2}-\omega_{L}^{2}}\right]\cos\left(\omega_{L}t\right)\right\}\left\{x_{1}^{\prime }-\frac{1}{M}\left[\frac{N_{1}f_{L,1}+N_{2}f_{L,2}}{\omega^{2}-\omega_{L}^{2}}+\frac{m_{2}N_{2}\left(\frac{f_{L,1}}{m_{1}}-\frac{f_{L,2}}{m_{2}}\right)}{\Omega_{12}^{2}-\omega_{L}^{2}}\right]\cos\left(\omega_{L}t\right)\right\}}\; \end{array}$$

and

(A8) $$\begin{array}{ccc} & & \;\;\;\;\;\;\;\;\;\;\;\;\;\;\;\rho_{1}^{\left(2\right)}\left(x_{2},x_{2}^{\prime },t\right)=N_{2}\left(N_{2}-1\right){\left(\frac{\alpha_{2}+C_{0,1}^{\prime }}{\pi }\right)}^{\frac{1}{2}}{\left(\frac{\alpha_{2}+C_{0,2}^{\prime }}{\pi }\right)}^{\frac{1}{2}}\times \\ & & \;\;\;\;\;\;\;\;\;\;\;\;\;\;\;\times e^{-\left(\alpha_{2}+C_{0,2}^{\prime }\right)\left\{{\left[x_{2}-x_{2}\left(t\right)\right]}^{2}+{\left[x_{2}^{\prime }-x_{2}\left(t\right)\right]}^{2}\right\}}e^{-2\left(\beta_{2}+C_{0,2}^{\prime }\right)\left[x_{2}-x_{2}\left(t\right)\right]\left[x_{2}^{\prime }-x_{2}\left(t\right)\right]}=\\ & & \;\;\;\;\;\;\;\;\;\;\;\;\;\;\;=N_{2}\left(N_{2}-1\right){\left(\frac{\alpha_{2}+C_{0,1}^{\prime }}{\pi }\right)}^{\frac{1}{2}}{\left(\frac{\alpha_{2}+C_{0,2}^{\prime }}{\pi }\right)}^{\frac{1}{2}}\times \\ & & \;\;\;\;\;\;\;\;\;\;\;\;\;\;\;\times e^{-\left(\alpha_{2}+C_{0,2}^{\prime }\right){\left\{x_{2}-\frac{1}{M}\left[\frac{N_{1}f_{L,1}+N_{2}f_{L,2}}{\omega^{2}-\omega_{L}^{2}}-\frac{m_{1}N_{1}\left(\frac{f_{L,1}}{m_{1}}-\frac{f_{L,2}}{m_{2}}\right)}{\Omega_{12}^{2}-\omega_{L}^{2}}\right]\cos\left(\omega_{L}t\right)\right\}}^{2}}\times \\ & & \;\;\;\;\;\;\;\;\;\;\;\;\;\;\;\times e^{-\left(\alpha_{2}+C_{0,2}^{\prime }\right){\left\{x_{2}^{\prime }-\frac{1}{M}\left[\frac{N_{1}f_{L,1}+N_{2}f_{L,2}}{\omega^{2}-\omega_{L}^{2}}-\frac{m_{1}N_{1}\left(\frac{f_{L,1}}{m_{1}}-\frac{f_{L,2}}{m_{2}}\right)}{\Omega_{12}^{2}-\omega_{L}^{2}}\right]\cos\left(\omega_{L}t\right)\right\}}^{2}}\times \\ & & \;\;\;\;\;\;\;\;\;\;\;\;\;\;\;\times e^{-2\left(\beta_{2}+C_{0,2}^{\prime }\right)\left\{x_{2}-\frac{1}{M}\left[\frac{N_{1}f_{L,1}+N_{2}f_{L,2}}{\omega^{2}-\omega_{L}^{2}}-\frac{m_{1}N_{1}\left(\frac{f_{L,1}}{m_{1}}-\frac{f_{L,2}}{m_{2}}\right)}{\Omega_{12}^{2}-\omega_{L}^{2}}\right]\cos\left(\omega_{L}t\right)\right\}\left\{x_{2}^{\prime }-\frac{1}{M}\left[\frac{N_{1}f_{L,1}+N_{2}f_{L,2}}{\omega^{2}-\omega_{L}^{2}}-\frac{m_{1}N_{1}\left(\frac{f_{L,1}}{m_{1}}-\frac{f_{L,2}}{m_{2}}\right)}{\Omega_{12}^{2}-\omega_{L}^{2}}\right]\cos\left(\omega_{L}t\right)\right\}}.\; \end{array}$$

From the mean-field wavefunction (43), the time-dependent intraspecies reduced two-particle density matrices are given at the mean-field level of theory by

(A9) $$\begin{array}{ccc} & & \rho_{1,MF}^{\left(2\right)}\left(x_{1},x_{1}^{\prime },x_{1}^{\prime \prime },x_{1}^{\prime \prime \prime },t\right)=N_{1}\left(N_{1}-1\right)\rho_{1,GP}^{\left(1\right)}\left(x_{1},x_{1}^{\prime \prime },t\right)\rho_{1,GP}^{\left(1\right)}\left(x_{1}^{\prime },x_{1}^{\prime \prime \prime },t\right),\\ & & \rho_{2,MF}^{\left(2\right)}\left(x_{2},x_{2}^{\prime },x_{2}^{\prime \prime },x_{2}^{\prime \prime \prime },t\right)=N_{2}\left(N_{2}-1\right)\rho_{2,GP}^{\left(1\right)}\left(x_{2},x_{2}^{\prime \prime },t\right)\rho_{2,GP}^{\left(1\right)}\left(x_{2}^{\prime },x_{2}^{\prime \prime \prime },t\right),\; \end{array}$$

with $\rho_{1,GP}^{\left(1\right)}\left(x_{1},x_{1}^{\prime },t\right)={\left(\frac{m_{1}\Omega_{1,GP}}{\pi }\right)}^{\frac{1}{2}}e^{-\frac{m_{1}\Omega_{1,GP}}{2}\left\{{\left[x_{1}-x_{1}\left(t\right)\right]}^{2}+{\left[x_{1}^{\prime }-x_{1}\left(t\right)\right]}^{2}\right\}}e^{+im_{1}{\dot{x}}_{1}\left(t\right)\left\{\left[x_{1}-x_{1}\left(t\right)\right]-\left[x_{1}^{\prime }-x_{1}\left(t\right)\right]\right\}}$ and $\rho_{2,GP}^{\left(1\right)}\left(x_{2},x_{2}^{\prime },t\right)={\left(\frac{m_{2}\Omega_{2,GP}}{\pi }\right)}^{\frac{1}{2}}e^{-\frac{m_{2}\Omega_{2,GP}}{2}\left\{{\left[x_{2}-x_{2}\left(t\right)\right]}^{2}+{\left[x_{2}^{\prime }-x_{2}\left(t\right)\right]}^{2}\right\}}e^{+im_{2}{\dot{x}}_{2}\left(t\right)\left\{\left[x_{2}-x_{2}\left(t\right)\right]-\left[x_{2}^{\prime }-x_{2}\left(t\right)\right]\right\}}$ [Equation (48)]. The two-particle mean-field intraspecies densities read $\rho_{1,MF}^{\left(2\right)}\left(x_{1},x_{1}^{\prime },t\right)=$$=N_{1}\left(N_{1}-1\right)\rho_{1,GP}^{\left(1\right)}\left(x_{1},t\right)\rho_{1,GP}^{\left(1\right)}\left(x_{1}^{\prime },t\right)$ and $\rho_{2,MF}^{\left(2\right)}\left(x_{2},x_{2}^{\prime },t\right)=N_{2}\left(N_{2}-1\right)\rho_{2,GP}^{\left(1\right)}\left(x_{2},t\right)\rho_{2,GP}^{\left(1\right)}\left(x_{2}^{\prime },t\right)$, where $\rho_{1,GP}^{\left(1\right)}\left(x_{1},t\right)={\left(\frac{m_{1}\Omega_{1,GP}}{\pi }\right)}^{\frac{1}{2}}e^{-m_{1}\Omega_{1,GP}{\left[x_{1}-x_{1}\left(t\right)\right]}^{2}}$ and $\rho_{2,GP}^{\left(1\right)}\left(x_{2},t\right)={\left(\frac{m_{2}\Omega_{2,GP}}{\pi }\right)}^{\frac{1}{2}}\times$$\times e^{-m_{2}\Omega_{2,GP}{\left[x_{2}-x_{2}\left(t\right)\right]}^{2}}$.

Therefore, with the mean-field quantities explicitly prescribed, one obtains at the limit of an infinite number of particles that

(A10) limN1→∞N2→∞ρ1(2)(x1,x1′,x1″,x1‴,t)N1(N1−1)=ρ1,GP(1)(x1,x1″,t)ρ1,GP(1)(x1′,x1‴,t),limN1→∞N2→∞ρ2(2)(x2,x2′,x2″,x2‴,t)N2(N2−1)=ρ2,GP(1)(x2,x2″,t)ρ2,GP(1)(x2′,x2‴,t)

for the reduced two-particle density matrices per particle and limN1→∞N2→∞ρ1(2)(x1,x1′,t)N1(N1−1)=$=\rho_{1,GP}^{\left(1\right)}\left(x_{1},t\right)\rho_{1,GP}^{\left(1\right)}\left(x_{1}^{\prime },t\right)$, limN1→∞N2→∞ρ2(2)(x2,x2′,t)N2(N2−1)=ρ2,GP(1)(x2,t)ρ2,GP(1)(x2′,t) for the time-dependent two-particle intraspecies densities per particle themselves.

## Appendix B. Further Details of Solving the Time-Dependent Coupled Gross–Pitaevskii Mean-Field Equations

In this appendix, we derive the solution of the Floquet non-linear Schrödinger equations of the driven mixture. To this end, we first obtain the respective solution of the driven single-species harmonic-interaction model and thereafter generalize the techniques used.

The Gross–Pitaevskii equation for the driven harmonic-interaction model (see (18)) is $\left[-\frac{1}{2m}\frac{\partial^{2}}{\partial x^{2}}+\frac{1}{2}m\omega^{2}x^{2}-xf_{L}\cos\left(\omega_{L}t\right)+\Lambda \int dx^{\prime }{\left|\phi \left(x^{\prime },t\right)\right|}^{2}{\left(x-x^{\prime }\right)}^{2}\right]\phi \left(x,t\right)=i\frac{\partial \phi (x,t)}{\partial t}$. We suspect that and examine whether the solution can be cast as

(A11) $$\phi \left(x,t\right)={\left(\frac{m\Omega_{GP}}{\pi }\right)}^{\frac{1}{4}}e^{-i\mu (t)}e^{-\frac{m\Omega_{GP}}{2}{\left[x-a\cos(\omega_{L}t)\right]}^{2}}e^{-im\omega_{L}a\sin\left(\omega_{L}t\right)x},$$

where the interaction-dressed frequency, $\Omega_{GP}$, time-dependent phase, μ(t), and amplitude of oscillations, *a*, are to be determined self-consistently. Substituting the function (A11) into the square brackets on the left-hand side of the time-dependent Gross–Pitaevskii equation, expanding the interaction term using the driven oscillations,

(A12) $$\begin{array}{ccc} & & {\left(x-x^{\prime }\right)}^{2}=x^{2}-2ax\cos\left(\omega_{L}t\right)+a^{2}\cos^{2}\left(\omega_{L}t\right)-2\left[x-a\cos\left(\omega_{L}t\right)\right]\left[x^{\prime }-a\cos\left(\omega_{L}t\right)\right]+\\ & & \;+{\left[x^{\prime }-a\cos\left(\omega_{L}t\right)\right]}^{2},\; \end{array}$$

and collecting terms, we find, for the square brackets, $\left[\;\;-\frac{1}{2m}\frac{\partial^{2}}{\partial x^{2}}+\frac{1}{2}m\left(\omega^{2}+\frac{2\Lambda }{m}\right)x^{2}-x\left(f_{L}+2a\Lambda \right)\cos\left(\omega_{L}t\right)+\Lambda a^{2}\cos^{2}\left(\omega_{L}t\right)+\frac{\Lambda }{2\Omega_{GP}}\right]$. This implies $\Omega_{GP}=\sqrt{\omega^{2}+\frac{2\Lambda }{m}}$ to hold, just like in the static problem. Next, as the solution of a driven oscillator with the interaction-dressed frequency $\Omega_{GP}$, the amplitude *a* must satisfy

(A13) $$a=\frac{f_{L}+2a\Lambda }{m\left(\Omega_{GP}^{2}-\omega_{L}^{2}\right)}\;⟹\;a=\frac{f_{L}}{m\left(\omega^{2}-\omega_{L}^{2}\right)},$$

i.e., resulting in the same amplitude of oscillations as for the bare driven harmonic oscillator and for the many-body solution in the laboratory frame. To verify consistency, we re-expand $\frac{1}{2}m\Omega_{GP}^{2}x^{2}=\frac{1}{2}m\Omega_{GP}^{2}{\left[x-a\cos(\omega_{L}t)\right]}^{2}+m\Omega_{GP}^{2}xa\cos\left(\omega_{L}t\right)-\frac{1}{2}m\Omega_{GP}^{2}a^{2}\cos^{2}\left(\omega_{L}t\right)$ and indeed find $m\Omega_{GP}^{2}a-\left(f_{L}+2a\Lambda \right)=m\omega_{L}^{2}a=f_{L}\frac{\omega_{L}^{2}}{\omega^{2}-\omega_{L}^{2}}$ for the linear-in-*x* term and $-\frac{1}{2}m\Omega_{GP}^{2}a^{2}\cos^{2}\left(\omega_{L}t\right)+\Lambda a^{2}\cos^{2}\left(\omega_{L}t\right)=-\frac{1}{2}m\omega^{2}a^{2}\cos^{2}\left(\omega_{L}t\right)=-\frac{f_{L}^{2}\omega^{2}}{2m{\left(\omega^{2}-\omega_{L}^{2}\right)}^{2}}\cos^{2}\left(\omega_{L}t\right)$ for the contribution to the phase, as is the case with the bare system. Finally, when operating from the left-hand side of the Gross–Pitaevskii equation with the kinetic-energy operator and from the right-hand side with the derivative in time on (A11), the remaining pieces of the time-dependent phase are collected, $\mu \left(t\right)=\left(\frac{\Omega_{GP}}{2}+\frac{\Lambda }{2\Omega_{GP}}\right)t+\int^{t}dt^{\prime }\left[\frac{f_{L}^{2}\omega_{L}^{2}}{2m{\left(\omega^{2}-\omega_{L}^{2}\right)}^{2}}\sin^{2}\left(\omega_{L}t^{\prime }\right)-\frac{f_{L}^{2}\omega^{2}}{2m{\left(\omega^{2}-\omega_{L}^{2}\right)}^{2}}\cos^{2}\left(\omega_{L}t^{\prime }\right)\right]=\left(\frac{\Omega_{GP}}{2}+\frac{\Lambda }{2\Omega_{GP}}\right)t+\int^{t}dt^{\prime }\left[\frac{1}{2}m{\dot{x}}^{2}\left(t\right)-\frac{1}{2}m\omega^{2}x^{2}\left(t\right)\right]$, determining the time-dependent mean-field solution (19). Summing up, despite the interaction dressing of the frequency of the (driven) oscillator from ω to $\Omega_{GP}$, the second and third contributions (see main text) to the time-dependent phase of ϕ(x,t) are renormalized ‘back’ to those of the bare system.

We employ the same strategy to derive the more involved solution of the coupled time-dependent Gross–Pitaevskii equations for the driven-mixture system. The coupled Gross–Pitaevskii equations for the harmonic-interaction model for a driven mixture are $\left\{-\frac{1}{2m_{1}}\frac{\partial^{2}}{\partial x_{1}^{2}}+\frac{1}{2}m_{1}\omega^{2}x_{1}^{2}-x_{1}f_{L,1}\cos\left(\omega_{L}t\right)+\Lambda_{1}\int dx_{1}^{\prime }|\phi_{1}\left(x_{1}^{\prime },t\right){|}^{2}{\left(x_{1}-x_{1}^{\prime }\right)}^{2}+\Lambda_{21}\int dx_{2}{\left|\phi_{2}\left(x_{2},t\right)\right|}^{2}{\left(x_{1}-x_{2}\right)}^{2}\right\}\phi_{1}\left(x_{1},t\right)=i\frac{\partial \phi_{1}\left(x_{1},t\right)}{\partial t}$, $\left\{-\frac{1}{2m_{2}}\frac{\partial^{2}}{\partial x_{2}^{2}}+\frac{1}{2}m_{2}\omega^{2}x_{2}^{2}-x_{2}f_{L,2}\cos\left(\omega_{L}t\right)+\Lambda_{2}\int dx_{2}^{\prime }|\phi_{2}\left(x_{2}^{\prime },t\right){|}^{2}{\left(x_{2}-x_{2}^{\prime }\right)}^{2}+\Lambda_{12}\int dx_{1}{\left|\phi_{1}\left(x_{1},t\right)\right|}^{2}{\left(x_{1}-x_{2}\right)}^{2}\right\}\phi_{2}\left(x_{2},t\right)=i\frac{\partial \phi_{2}\left(x,t\right)}{\partial t}$ (see (44)). We guess that and check whether the solution can be cast as

(A14) $$\begin{array}{ccc} & & \phi_{1}\left(x_{1},t\right)={\left(\frac{m_{1}\Omega_{1,GP}}{\pi }\right)}^{\frac{1}{4}}e^{-i\mu_{1}\left(t\right)}e^{-\frac{m_{1}\Omega_{GP,1}}{2}{\left[x_{1}-a_{1}\cos\left(\omega_{L}t\right)\right]}^{2}}e^{-im_{1}\omega_{L}a_{1}\sin\left(\omega_{L}t\right)x_{1}},\\ & & \phi_{2}\left(x_{2},t\right)={\left(\frac{m_{2}\Omega_{2,GP}}{\pi }\right)}^{\frac{1}{4}}e^{-i\mu_{2}\left(t\right)}e^{-\frac{m_{2}\Omega_{GP,2}}{2}{\left[x_{2}-a_{2}\cos\left(\omega_{L}t\right)\right]}^{2}}e^{-im_{2}\omega_{L}a_{2}\sin\left(\omega_{L}t\right)x_{2}},\; \end{array}$$

where the interaction-dressed frequencies, $\Omega_{1,GP}$ and $\Omega_{2,GP}$, time-dependent phases, $\mu_{1}\left(t\right)$ and $\mu_{2}\left(t\right)$, and amplitudes of oscillations, $a_{1}$ and $a_{2}$, are to be determined self-consistently. Substituting the functions (A14) into the curly brackets on the left-hand sides of the two coupled time-dependent Gross–Pitaevskii equations, expanding the interaction terms with the respective oscillation of each orbital, and collecting the terms in each set of curly brackets, we find

(A15) $$\begin{array}{ccc} & & \{-\frac{1}{2m_{1}}\frac{\partial^{2}}{\partial x_{1}^{2}}+\frac{1}{2}m_{1}\left[\omega^{2}+\frac{2(\Lambda_{1}+\Lambda_{21})}{m_{1}}\right]x_{1}^{2}-x_{1}\left(f_{L,1}+2a_{1}\Lambda_{1}+2a_{2}\Lambda_{21}\right)\cos\left(\omega_{L}t\right)+\\ & & +\left(\Lambda_{1}a_{1}^{2}+\Lambda_{21}a_{2}^{2}\right)\cos^{2}\left(\omega_{L}t\right)+\frac{\Lambda_{1}}{2\Omega_{1,GP}}+\frac{\Lambda_{21}}{2\Omega_{2,GP}}\},\\ & & \{-\frac{1}{2m_{2}}\frac{\partial^{2}}{\partial x_{2}^{2}}+\frac{1}{2}m_{2}\left[\omega^{2}+\frac{2(\Lambda_{2}+\Lambda_{12})}{m_{2}}\right]x_{2}^{2}-x_{2}\left(f_{L,2}+2a_{2}\Lambda_{2}+2a_{1}\Lambda_{12}\right)\cos\left(\omega_{L}t\right)+\\ & & +\left(\Lambda_{2}a_{2}^{2}+\Lambda_{12}a_{1}^{2}\right)\cos^{2}\left(\omega_{L}t\right)+\frac{\Lambda_{2}}{2\Omega_{2,GP}}+\frac{\Lambda_{12}}{2\Omega_{1,GP}}\}. \end{array}$$

These imply that $\Omega_{1,GP}=\sqrt{\omega^{2}+\frac{2(\Lambda_{1}+\Lambda_{21})}{m_{1}}}$ and $\Omega_{2,GP}=\sqrt{\omega^{2}+\frac{2(\Lambda_{2}+\Lambda_{12})}{m_{2}}}$ hold, just as in the static mixture’s problem.

Next, as the solutions of driven coupled (many-particle) oscillators with the interaction-dressed frequencies $\Omega_{1,GP}$ and $\Omega_{2,GP}$, the amplitudes $a_{1}$ and $a_{2}$ have to satisfy the coupled linear relations

(A16) $$a_{1}=\frac{f_{L,1}+2a_{1}\Lambda_{1}+2a_{2}\Lambda_{21}}{m_{1}\left(\Omega_{1,GP}^{2}-\omega_{L}^{2}\right)},\;a_{2}=\frac{f_{L,2}+2a_{2}\Lambda_{2}+2a_{1}\Lambda_{12}}{m_{2}\left(\Omega_{2,GP}^{2}-\omega_{L}^{2}\right)},$$

the solution of which is readily found:

(A17) $$\begin{array}{ccc} & & a_{1}=\frac{1}{m_{1}\Lambda_{12}+m_{2}\Lambda_{21}}\left[\frac{\Lambda_{12}f_{L,1}+\Lambda_{21}f_{L,2}}{\omega^{2}-\omega_{L}^{2}}+\frac{m_{2}\Lambda_{21}\left(\frac{f_{L,1}}{m_{1}}-\frac{f_{L,2}}{m_{2}}\right)}{\Omega_{12}^{2}-\omega_{L}^{2}}\right],\\ & & a_{2}=\frac{1}{m_{1}\Lambda_{12}+m_{2}\Lambda_{21}}\left[\frac{\Lambda_{12}f_{L,1}+\Lambda_{21}f_{L,2}}{\omega^{2}-\omega_{L}^{2}}-\frac{m_{1}\Lambda_{12}\left(\frac{f_{L,1}}{m_{1}}-\frac{f_{L,2}}{m_{2}}\right)}{\Omega_{12}^{2}-\omega_{L}^{2}}\right].\; \end{array}$$

Interestingly, these are the same amplitudes of oscillations, with the same poles, as for the many-body solution (see (34)). We may say that the non-linear mean-field terms renormalize the poles from the values of the interaction-dressed oscillators $\Omega_{1,GP}^{2}$ and $\Omega_{2,GP}^{2}$ (see (A16)) to the oscillators’ frequencies of the many-body problem, $\omega^{2}$ and $\Omega_{12}^{2}$ (see (A17)).

To proceed for the terms linear in $x_{1}$ and $x_{2}$, after re-expanding $\frac{1}{2}m_{1}\Omega_{1,GP}^{2}x_{1}^{2}=$$=\frac{1}{2}m_{1}\Omega_{1,GP}^{2}{\left[x_{1}-a_{1}\cos\left(\omega_{L}t\right)\right]}^{2}+m_{1}\Omega_{1,GP}^{2}x_{1}a_{1}\cos\left(\omega_{L}t\right)-\frac{1}{2}m_{1}\Omega_{1,GP}^{2}a_{1}^{2}\cos^{2}\left(\omega_{L}t\right)$ and likewise for $\frac{1}{2}m_{2}\Omega_{2,GP}^{2}x_{2}^{2}$, we find

(A18) $$\begin{array}{ccc} & & m_{1}\Omega_{1,GP}^{2}a_{1}-\left(f_{L,1}+2a_{1}\Lambda_{1}+2a_{2}\Lambda_{21}\right)=\\ & & =\frac{m_{1}\omega_{L}^{2}}{m_{1}\Lambda_{12}+m_{2}\Lambda_{21}}\left[\frac{\Lambda_{12}f_{L,1}+\Lambda_{21}f_{L,2}}{\omega^{2}-\omega_{L}^{2}}+\frac{m_{2}\Lambda_{21}\left(\frac{f_{L,1}}{m_{1}}-\frac{f_{L,2}}{m_{2}}\right)}{\Omega_{12}^{2}-\omega_{L}^{2}}\right]=m_{1}\omega_{L}^{2}a_{1},\\ & & m_{2}\Omega_{2,GP}^{2}a_{2}-\left(f_{L,2}+2a_{2}\Lambda_{2}+2a_{1}\Lambda_{12}\right)=\\ & & =\frac{m_{2}\omega_{L}^{2}}{m_{1}\Lambda_{12}+m_{2}\Lambda_{21}}\left[\frac{\Lambda_{12}f_{L,1}+\Lambda_{21}f_{L,2}}{\omega^{2}-\omega_{L}^{2}}-\frac{m_{1}\Lambda_{12}\left(\frac{f_{L,1}}{m_{1}}-\frac{f_{L,2}}{m_{2}}\right)}{\Omega_{12}^{2}-\omega_{L}^{2}}\right]=m_{2}\omega_{L}^{2}a_{2},\; \end{array}$$

and for the contributions to the phases

(A19) $$\begin{array}{ccc} & & -\frac{1}{2}m_{1}\Omega_{1,GP}^{2}a_{1}^{2}\cos^{2}\left(\omega_{L}t\right)+\left(\Lambda_{1}a_{1}^{2}+\Lambda_{21}a_{2}^{2}\right)\cos^{2}\left(\omega_{L}t\right)=\\ & & =-\left[\frac{1}{2}m_{1}\omega^{2}a_{1}^{2}+\Lambda_{21}\left(a_{1}^{2}-a_{2}^{2}\right)\right]\cos^{2}\left(\omega_{L}t\right),\\ & & -\frac{1}{2}m_{2}\Omega_{2,GP}^{2}a_{2}^{2}\cos^{2}\left(\omega_{L}t\right)+\left(\Lambda_{2}a_{2}^{2}+\Lambda_{12}a_{1}^{2}\right)\cos^{2}\left(\omega_{L}t\right)=\\ & & =-\left[\frac{1}{2}m_{2}\omega^{2}a_{2}^{2}-\Lambda_{12}\left(a_{1}^{2}-a_{2}^{2}\right)\right]\cos^{2}\left(\omega_{L}t\right).\; \end{array}$$

The meaning of the above is that the linear terms (A18) are appropriately renormalized in comparison with the many-body treatment by the non-linear interaction terms, whereas the phases (A19) are not (also see below). In summary, the mean-field treatment correctly captures the amplitudes of oscillations and contributions of the linear terms (associated with operation of the kinetic-energy operator) to the wavefunction, but not the contribution of the time-dependent phases. These contributions do not match both poles’ frequencies, $\omega^{2}$ and $\Omega_{12}^{2}$, appearing in the oscillations’ terms at the mean-field level.

Adding up all terms, we have, for the time-dependent phases in (A14),

(A20) $$\begin{array}{ccc} & & \mu_{1}\left(t\right)=\left(\frac{\Omega_{1,GP}}{2}+\frac{\Lambda_{1}}{2\Omega_{1,GP}}+\frac{\Lambda_{21}}{2\Omega_{2,GP}}\right)t+\\ & & +\int^{t}dt^{\prime }\left\{\frac{1}{2}m_{1}\omega_{L}^{2}a_{1}^{2}\sin^{2}\left(\omega_{L}t^{\prime }\right)-\left[\frac{1}{2}m_{1}\omega^{2}a_{1}^{2}+\Lambda_{21}\left(a_{1}^{2}-a_{2}^{2}\right)\right]\cos^{2}\left(\omega_{L}t^{\prime }\right)\right\}=\\ & & =\mu_{1,F}-\frac{1}{8}\left[m_{1}\left(\omega^{2}+\omega_{L}^{2}\right)a_{1}^{2}+2\Lambda_{21}\left(a_{1}^{2}-a_{2}^{2}\right)\right]\sin\left(2\omega_{L}t\right),\\ & & \mu_{2}\left(t\right)=\left(\frac{\Omega_{2,GP}}{2}+\frac{\Lambda_{2}}{2\Omega_{2,GP}}+\frac{\Lambda_{12}}{2\Omega_{1,GP}}\right)t+\\ & & +\int^{t}dt^{\prime }\left\{\frac{1}{2}m_{2}\omega_{L}^{2}a_{2}^{2}\sin^{2}\left(\omega_{L}t^{\prime }\right)-\left[\frac{1}{2}m_{2}\omega^{2}a_{2}^{2}-\Lambda_{12}\left(a_{1}^{2}-a_{2}^{2}\right)\right]\cos^{2}\left(\omega_{L}t^{\prime }\right)\right\}=\\ & & =\mu_{2,F}-\frac{1}{8}\left[m_{2}\left(\omega^{2}+\omega_{L}^{2}\right)a_{2}^{2}-2\Lambda_{12}\left(a_{1}^{2}-a_{2}^{2}\right)\right]\sin\left(2\omega_{L}t\right),\; \end{array}$$

where

(A21) $$\begin{array}{ccc} & & \mu_{1,F}=\left(\frac{\Omega_{1,GP}}{2}+\frac{\Lambda_{1}}{2\Omega_{1,GP}}+\frac{\Lambda_{21}}{2\Omega_{2,GP}}\right)-\frac{1}{4}\left[m_{1}\left(\omega^{2}-\omega_{L}^{2}\right)a_{1}^{2}+2\Lambda_{21}\left(a_{1}^{2}-a_{2}^{2}\right)\right],\\ & & \mu_{2,F}=\left(\frac{\Omega_{2,GP}}{2}+\frac{\Lambda_{2}}{2\Omega_{2,GP}}+\frac{\Lambda_{12}}{2\Omega_{1,GP}}\right)-\frac{1}{4}\left[m_{2}\left(\omega^{2}-\omega_{L}^{2}\right)a_{2}^{2}-2\Lambda_{12}\left(a_{1}^{2}-a_{2}^{2}\right)\right]\; \end{array}$$

are the quasichemical potentials, determining the time-dependent mean-field solution of the driven mixture in the main text (see (45)). After substitution of the amplitudes $a_{1}$ and $a_{2}$, the final expressions for $\mu_{1,F}$ and $\mu_{2,F}$ are obtained (see (46)).

The final expression for the quasienergy per particle at the mean-field level of theory simplifies, since the interspecies interaction terms in (A21) cancel each other upon the addition $N_{1}\mu_{1}\left(t\right)+N_{2}\mu_{2}\left(t\right)$ within the time-dependent phase of the mean-field Floquet wavefunction, and reads:

(A22) $$\begin{array}{ccc} & & \varepsilon_{F}^{GP}=\frac{N_{1}}{N}\frac{\Omega_{1,GP}}{2}+\frac{N_{2}}{N}\frac{\Omega_{2,GP}}{2}-\frac{1}{4}\left(\omega^{2}-\omega_{L}^{2}\right)\left(\frac{N_{1}}{N}m_{1}a_{1}^{2}+\frac{N_{2}}{N}m_{2}a_{2}^{2}\right). \end{array}$$

Upon substitution of the oscillations’ amplitudes $a_{1}$ and $a_{2}$, the expression for the mixture’s quasienergy given in the main text is obtained (see (49)).

## Appendix C. Angular-Momentum Variances in Presence of Translations and Boosts

In this appendix, we derive expressions for the angular-momentum variance of translated and boosted many-particle wavefunctions in terms of quantities computed from the un-translated and un-boosted wavefunctions. We start with the analysis for single-species bosons (for the case of translations only, see [74]), then generalize it to the two-species mixture or interacting-impurity system.

Consider the translated–boosted many-particle wavefunction in two spatial dimensions, $e^{-i({\hat{P}}_{X}a+{\hat{P}}_{Y}b)}e^{+i(\hat{X}\alpha +\hat{Y}\beta )}\Psi \left(X,Y\right)\equiv \Psi \left(a,b;\alpha ,\beta \right)$, where ${\hat{P}}_{X}=\sum_{j=1}^{N}{\hat{p}}_{x,j},{\hat{P}}_{Y}=\sum_{j=1}^{N}{\hat{p}}_{y,j}$ and $\hat{X}=\sum_{j=1}^{N}{\hat{x}}_{j},\hat{Y}=\sum_{j=1}^{N}{\hat{y}}_{j}$, and the shorthand collective notation for the coordinates $\Psi \left(X,Y\right)\equiv \Psi \left(x_{1},y_{1},\ldots ,x_{N},y_{N}\right)$ is implied. The order of the translation and boost operators does not matter for the computation of matrix elements. The wavefunction (before translations and boosts) as well as the translation and boost operators themselves can be time dependent. What are the implications for the variances of observables when computed with respect to the translated–boosted wavefunction Ψ(a,b;α,β)?

For the position operator $\hat{X}$ (and equivalently for $\hat{Y}$), we have $e^{-i(\hat{X}\alpha +\hat{Y}\beta )}e^{+i({\hat{P}}_{X}a+{\hat{P}}_{Y}b)}\hat{X}\times$ $\times e^{-i({\hat{P}}_{X}a+{\hat{P}}_{Y}b)}e^{+i(\hat{X}\alpha +\hat{Y}\beta )}=\hat{X}+Na$, and using its square, one has

(A23) $$\frac{1}{N}\Delta_{\hat{X}}^{2}{|}_{\Psi (a,b;\alpha ,\beta )}=\frac{1}{N}\Delta_{\hat{X}}^{2}{|}_{\Psi }.$$

Similarly, for the momentum operator ${\hat{P}}_{X}$ (and equivalently for ${\hat{P}}_{Y}$), we have $e^{-i(\hat{X}\alpha +\hat{Y}\beta )}\times$$\times e^{+i({\hat{P}}_{X}a+{\hat{P}}_{Y}b)}{\hat{P}}_{X}e^{-i({\hat{P}}_{X}a+{\hat{P}}_{Y}b)}e^{+i(\hat{X}\alpha +\hat{Y}\beta )}={\hat{P}}_{X}+N\alpha$, and using its square leads to

(A24) $$\frac{1}{N}\Delta_{{\hat{P}}_{X}}^{2}{|}_{\Psi (a,b;\alpha ,\beta )}=\frac{1}{N}\Delta_{{\hat{P}}_{X}}^{2}{|}_{\Psi },$$

i.e., both the position and momentum variances are invariant under translations, boosts, and their combined operation.

For the angular-momentum operator ${\hat{L}}_{Z}=\sum_{j=1}^{N}\left({\hat{x}}_{j}{\hat{p}}_{y,j}-{\hat{y}}_{j}{\hat{p}}_{x,j}\right)$, the situation is more intricate because it consists of the position and momentum operators, which do not commute with each other. From

(A25) $$\begin{array}{ccc} & & e^{-i(\hat{X}\alpha +\hat{Y}\beta )}e^{+i({\hat{P}}_{X}a+{\hat{P}}_{Y}b)}{\hat{L}}_{Z}e^{-i({\hat{P}}_{X}a+{\hat{P}}_{Y}b)}e^{+i(\hat{X}\alpha +\hat{Y}\beta )}=\\ & & ={\hat{L}}_{Z}+a{\hat{P}}_{Y}-b{\hat{P}}_{X}+\beta \hat{X}-\alpha \hat{Y}+N\left(a\beta -b\alpha \right)\; \end{array}$$

and its square, we have

(A26) $$\begin{array}{ccc} & & \frac{1}{N}\Delta_{{\hat{L}}_{Z}}^{2}{|}_{\Psi (a,b;\alpha ,\beta )}=\frac{1}{N}\Delta_{{\hat{L}}_{Z}}^{2}{|}_{\Psi }+a^{2}\frac{1}{N}\Delta_{{\hat{P}}_{Y}}^{2}{|}_{\Psi }+b^{2}\frac{1}{N}\Delta_{{\hat{P}}_{X}}^{2}{|}_{\Psi }+\alpha^{2}\frac{1}{N}\Delta_{\hat{Y}}^{2}{|}_{\Psi }+\beta^{2}\frac{1}{N}\Delta_{\hat{X}}^{2}{|}_{\Psi }+\\ & & \;+\frac{1}{N}[a\left(\langle \Psi |{\hat{L}}_{Z}{\hat{P}}_{Y}+{\hat{P}}_{Y}{\hat{L}}_{Z}|\Psi \rangle -2\langle \Psi |{\hat{L}}_{Z}\left|\Psi \rangle \langle \Psi \right|{\hat{P}}_{Y}|\Psi \rangle \right)-\\ & & \;-b\left(\langle \Psi |{\hat{L}}_{Z}{\hat{P}}_{X}+{\hat{P}}_{X}{\hat{L}}_{Z}|\Psi \rangle -2\langle \Psi |{\hat{L}}_{Z}\left|\Psi \rangle \langle \Psi \right|{\hat{P}}_{X}|\Psi \rangle \right)-\\ & & \;-2ab\left(\langle \Psi |{\hat{P}}_{Y}{\hat{P}}_{X}|\Psi \rangle -\langle \Psi |{\hat{P}}_{Y}\left|\Psi \rangle \langle \Psi \right|{\hat{P}}_{X}|\Psi \rangle \right)+\\ & & \;+\beta \left(\langle \Psi |{\hat{L}}_{Z}\hat{X}+\hat{X}{\hat{L}}_{Z}|\Psi \rangle -2\langle \Psi |{\hat{L}}_{Z}\left|\Psi \rangle \langle \Psi \right|\hat{X}|\Psi \rangle \right)-\\ & & \;-\alpha \left(\langle \Psi |{\hat{L}}_{Z}\hat{Y}+\hat{Y}{\hat{L}}_{Z}|\Psi \rangle -2\langle \Psi |{\hat{L}}_{Z}\left|\Psi \rangle \langle \Psi \right|\hat{Y}|\Psi \rangle \right)-\\ & & \;-2\alpha \beta \left(\langle \Psi |\hat{X}\hat{Y}|\Psi \rangle -\langle \Psi |\hat{X}\left|\Psi \rangle \langle \Psi \right|\hat{Y}|\Psi \rangle \right)-\\ & & \;-a\alpha \left(\langle \Psi |{\hat{P}}_{Y}\hat{Y}+\hat{Y}{\hat{P}}_{Y}|\Psi \rangle -2\langle \Psi |{\hat{P}}_{Y}\left|\Psi \rangle \langle \Psi \right|\hat{Y}|\Psi \rangle \right)-\\ & & \;-b\beta \left(\langle \Psi |{\hat{P}}_{X}\hat{X}+\hat{X}{\hat{P}}_{X}|\Psi \rangle -2\langle \Psi |{\hat{P}}_{X}\left|\Psi \rangle \langle \Psi \right|\hat{X}|\Psi \rangle \right)+\\ & & \;+2a\beta \left(\langle \Psi |{\hat{P}}_{Y}\hat{X}|\Psi \rangle -\langle \Psi |{\hat{P}}_{Y}\left|\Psi \rangle \langle \Psi \right|\hat{X}|\Psi \rangle \right)+\\ & & \;+2b\alpha \left(\langle \Psi |{\hat{P}}_{X}\hat{Y}|\Psi \rangle -\langle \Psi |{\hat{P}}_{X}\left|\Psi \rangle \langle \Psi \right|\hat{Y}|\Psi \rangle \right)].\; \end{array}$$

Equation (A26) warrants a discussion. We see that the angular-momentum variance of a translated–boosted wavefunction can be decomposed into various terms of physical meaning computed from the un-translated/boosted wavefunction. These include, for a start, the angular-momentum variance of the wavefunction and the momentum and position variances in each direction (see the first line in (A26)). Then, there are terms that originate from translations only, as well as ‘interference’ between them, followed by terms that originate from boosts only, as well as ‘interference’ between them, and, finally, ‘interference’ terms between translations and boosts. For the most general wavefunction, all involved expectation values contribute. When symmetries set in (see below), or when the wavefunction is real (excluding a trivial phase factor), corresponding terms fall.

For our needs, let us examine translated and boosted rotationally symmetric systems. Consider the ground state Ψ of an interacting many-boson system in a rotationally symmetric trap (to be more precise, we do not consider the ground state of a rotationally symmetric system in the rotating frame). Clearly, $\frac{1}{N}\Delta_{{\hat{L}}_{Z}}^{2}{|}_{\Psi }=0$ holds. Now, when the ground state is translated and boosted, the angular-momentum variance can be written as

(A27) $$\frac{1}{N}\Delta_{{\hat{L}}_{Z}}^{2}{|}_{\Psi (a,b;\alpha ,\beta )}=a^{2}\frac{1}{N}\Delta_{{\hat{P}}_{Y}}^{2}{|}_{\Psi }+b^{2}\frac{1}{N}\Delta_{{\hat{P}}_{X}}^{2}{|}_{\Psi }+\alpha^{2}\frac{1}{N}\Delta_{\hat{Y}}^{2}{|}_{\Psi }+\beta^{2}\frac{1}{N}\Delta_{\hat{X}}^{2}{|}_{\Psi },$$

and seen to solely be described by the momentum and position variances along the *y* and *x* directions multiplied by the respective values of the translations and boosts along the *x* and *y* directions.

Interestingly, the angular-momentum variance of a translated-boosted many-boson system $\frac{1}{N}\Delta_{{\hat{L}}_{Z}}^{2}{|}_{\Psi (a,b;\alpha ,\beta )}$ differs at the many-body and mean-field levels of theory, i.e., when a,b≠0, α,β≠0, and λ≠0. This, as can be seen in (A27), is because of the respective many-body and mean-field momentum, $\frac{1}{N}\Delta_{{\hat{P}}_{X}}^{2}{|}_{\Psi }$ and $\frac{1}{N}\Delta_{{\hat{P}}_{Y}}^{2}{|}_{\Psi }$, and position, $\frac{1}{N}\Delta_{\hat{X}}^{2}{|}_{\Psi }$ and $\frac{1}{N}\Delta_{\hat{Y}}^{2}{|}_{\Psi }$, variances. For instance, in harmonic traps, these variances do not dependent on the interaction strength at the many-body level of theory, but are dressed by the interaction at the mean-field level of theory (see, in this context, [64]). The analytical result (A27) is employed to compute and analyze the findings for the angular-momentum variance of a many-boson Floquet state in the main text. Generally, as mentioned above, in the absence of spatial symmetries, more terms contribute to the translated and boosted angular-momentum variance (see (A26)).

We now generalize the above analysis to the mixture or impurity system. The notation used hereafter is a straightforward generalization of the notation employed above in the single-species case. Starting from the many-particle wavefunction $\Psi (X_{1},Y_{1},X_{2},Y_{2})$, we note that each species can be translated and boosted differently, $e^{-i({\hat{P}}_{X_{1}}a_{1}+{\hat{P}}_{Y_{1}}b_{1})}e^{+i({\hat{X}}_{1}\alpha_{1}+{\hat{Y}}_{1}\beta_{1})}\times$$\times e^{-i({\hat{P}}_{X_{2}}a_{2}+{\hat{P}}_{Y_{2}}b_{2})}e^{+i({\hat{X}}_{2}\alpha_{2}+{\hat{Y}}_{2}\beta_{1})}\Psi \left(X_{1},Y_{1},X_{2},Y_{2}\right)\equiv \Psi \left(\vec{a},\vec{b};\vec{\alpha },\vec{\beta }\right)$, as is, for instance, the result in the main text for the steered species 1 bosons embedded in un-steered species 2 bosons (see Section 4).

With $\Psi (\vec{a},\vec{b};\vec{\alpha },\vec{\beta })$, it is straightforward to see that the variance of the (mass-weighted) position operator, $\frac{N}{M}\left(m_{1}{\hat{X}}_{1}+m_{2}{\hat{X}}_{2}\right)$, total momentum operator, ${\hat{P}}_{1}+{\hat{P}}_{2}$, or any linear combination of ${\hat{X}}_{1}$ and ${\hat{X}}_{2}$ or of ${\hat{P}}_{1}$ and ${\hat{P}}_{2}$ is invariant under translations, boosts, and combinations thereof.

For the variance of the angular-momentum operator of either species 1 or 2, we just use (A26) with the corresponding notations, since $\frac{1}{N_{1}}\Delta_{{\hat{L}}_{Z_{1}}}^{2}{|}_{\Psi (\vec{a},\vec{b};\vec{\alpha },\vec{\beta })}=\frac{1}{N_{1}}\Delta_{{\hat{L}}_{Z_{1}}}^{2}{|}_{\Psi (a_{1},b_{1};\alpha_{1},\beta_{1})}$ and $\frac{1}{N_{2}}\Delta_{{\hat{L}}_{Z_{2}}}^{2}{|}_{\Psi (\vec{a},\vec{b};\vec{\alpha },\vec{\beta })}=\frac{1}{N_{2}}\Delta_{{\hat{L}}_{Z_{2}}}^{2}{|}_{\Psi (a_{2},b_{2};\alpha_{2},\beta_{2})}$. Symmetry can be of assistance as well, yet note that $\frac{1}{N_{1}}\Delta_{{\hat{L}}_{Z_{1}}}^{2}{|}_{\Psi }$ and $\frac{1}{N_{2}}\Delta_{{\hat{L}}_{Z_{2}}}^{2}{|}_{\Psi }$ generally do not vanish at the many-body level (and differences between the mean-field and many-body treatments can thus occur), even for the ground state of a mixture in spherically symmetric traps, due to the coupling introduced by the interspecies interaction $\lambda_{12}$ [121]. All in all, in our case, we have

(A28) $$\begin{array}{ccc} & & \frac{1}{N_{1}}\Delta_{{\hat{L}}_{Z_{1}}}^{2}{|}_{\Psi (\vec{a},\vec{b};\vec{\alpha },\vec{\beta })}=\frac{1}{N_{1}}\Delta_{{\hat{L}}_{Z_{1}}}^{2}{|}_{\Psi }+a_{1}^{2}\frac{1}{N_{1}}\Delta_{{\hat{P}}_{Y_{1}}}^{2}{|}_{\Psi }+b_{1}^{2}\frac{1}{N_{1}}\Delta_{{\hat{P}}_{X_{1}}}^{2}{|}_{\Psi }+\alpha_{1}^{2}\frac{1}{N_{1}}\Delta_{{\hat{Y}}_{1}}^{2}{|}_{\Psi }+\beta_{1}^{2}\frac{1}{N_{1}}\Delta_{{\hat{X}}_{1}}^{2}{|}_{\Psi },\\ & & \frac{1}{N_{2}}\Delta_{{\hat{L}}_{Z_{2}}}^{2}{|}_{\Psi (\vec{a},\vec{b};\vec{\alpha },\vec{\beta })}=\frac{1}{N_{2}}\Delta_{{\hat{L}}_{Z_{2}}}^{2}{|}_{\Psi }+a_{2}^{2}\frac{1}{N_{2}}\Delta_{{\hat{P}}_{Y_{2}}}^{2}{|}_{\Psi }+b_{2}^{2}\frac{1}{N_{2}}\Delta_{{\hat{P}}_{X_{2}}}^{2}{|}_{\Psi }+\alpha_{2}^{2}\frac{1}{N_{2}}\Delta_{{\hat{Y}}_{2}}^{2}{|}_{\Psi }+\beta_{2}^{2}\frac{1}{N_{2}}\Delta_{{\hat{X}}_{2}}^{2}{|}_{\Psi }\; \end{array}$$

for the angular-momentum variances of the individual species in the driven mixture.

To derive the working expression for the variance of the total angular-momentum operator ${\hat{L}}_{Z}={\hat{L}}_{Z_{1}}+{\hat{L}}_{Z_{2}}$ of the mixture, we begin from the transformed quantity

(A29) $$\begin{array}{ccc} & & e^{-i({\hat{X}}_{2}\alpha_{2}+{\hat{Y}}_{2}\beta_{1})}e^{+i({\hat{P}}_{X_{2}}a_{2}+{\hat{P}}_{Y_{2}}b_{2})}e^{-i({\hat{X}}_{1}\alpha_{1}+{\hat{Y}}_{1}\beta_{1})}e^{+i({\hat{P}}_{X_{1}}a_{1}+{\hat{P}}_{Y_{1}}b_{1})}{\hat{L}}_{Z}\times \\ & & \times e^{-i({\hat{P}}_{X_{1}}a_{1}+{\hat{P}}_{Y_{1}}b_{1})}e^{+i({\hat{X}}_{1}\alpha_{1}+{\hat{Y}}_{1}\beta_{1})}e^{-i({\hat{P}}_{X_{2}}a_{2}+{\hat{P}}_{Y_{2}}b_{2})}e^{+i({\hat{X}}_{2}\alpha_{2}+{\hat{Y}}_{2}\beta_{1})}=\\ & & ={\hat{L}}_{Z}+a_{1}{\hat{P}}_{Y_{1}}+a_{2}{\hat{P}}_{Y_{2}}-b_{1}{\hat{P}}_{X_{1}}-b_{2}{\hat{P}}_{X_{2}}+\beta_{1}{\hat{X}}_{1}+\beta_{2}{\hat{X}}_{2}-\alpha_{1}{\hat{Y}}_{1}-\alpha_{2}{\hat{Y}}_{2}+\\ & & +N_{1}\left(a_{1}\beta_{1}-b_{1}\alpha_{1}\right)+N_{2}\left(a_{2}\beta_{2}-b_{2}\alpha_{2}\right)\; \end{array}$$

and its square. The final result is lengthier than (A26), but is otherwise straightforward. For spherically symmetric traps, many terms fall, and the final result can be written as

(A30) $$\begin{array}{ccc} & & \frac{1}{N}\Delta_{{\hat{L}}_{Z}}^{2}{|}_{\Psi (\vec{a},\vec{b};\vec{\alpha },\vec{\beta })}=\\ & & =\frac{1}{N}\Delta_{a_{1}{\hat{P}}_{Y_{1}}+a_{2}{\hat{P}}_{Y_{2}}}^{2}{|}_{\Psi }+\frac{1}{N}\Delta_{b_{1}{\hat{P}}_{X_{1}}+b_{2}{\hat{P}}_{X_{2}}}^{2}{|}_{\Psi }+\frac{1}{N}\Delta_{\beta_{1}{\hat{X}}_{1}+\beta_{2}{\hat{X}}_{2}}^{2}{|}_{\Psi }+\frac{1}{N}\Delta_{\alpha_{1}{\hat{Y}}_{1}+\alpha_{2}{\hat{Y}}_{2}}^{2}{|}_{\Psi }+\\ & & =\frac{N_{1}}{N}\left(a_{1}^{2}\frac{1}{N_{1}}\Delta_{{\hat{P}}_{Y_{1}}}^{2}{|}_{\Psi }+b_{1}^{2}\frac{1}{N_{1}}\Delta_{{\hat{P}}_{X_{1}}}^{2}{|}_{\Psi }+\alpha_{1}^{2}\frac{1}{N_{1}}\Delta_{{\hat{Y}}_{1}}^{2}{|}_{\Psi }+\beta_{1}^{2}\frac{1}{N_{1}}\Delta_{{\hat{X}}_{1}}^{2}{|}_{\Psi }\right)+\\ & & +\frac{N_{2}}{N}\left(a_{2}^{2}\frac{1}{N_{2}}\Delta_{{\hat{P}}_{Y_{2}}}^{2}{|}_{\Psi }+b_{2}^{2}\frac{1}{N_{2}}\Delta_{{\hat{P}}_{X_{2}}}^{2}{|}_{\Psi }+\alpha_{2}^{2}\frac{1}{N_{2}}\Delta_{{\hat{Y}}_{2}}^{2}{|}_{\Psi }+\beta_{2}^{2}\frac{1}{N_{2}}\Delta_{{\hat{X}}_{2}}^{2}{|}_{\Psi }\right)+\\ & & +2\frac{1}{N}\left(a_{1}a_{2}\langle \Psi |{\hat{P}}_{Y_{1}}{\hat{P}}_{Y_{2}}|\Psi \rangle +b_{1}b_{2}\langle \Psi |{\hat{P}}_{X_{1}}{\hat{P}}_{X_{2}}|\Psi \rangle +\alpha_{1}\alpha_{2}\langle \Psi |{\hat{Y}}_{1}{\hat{Y}}_{2}|\Psi \rangle +\beta_{1}\beta_{2}\langle \Psi |{\hat{X}}_{1}{\hat{X}}_{2}|\Psi \rangle \right).\; \end{array}$$

When applied to the driven mixture in the main text, the angular-momentum variance (A30) can be expressed using the variances of time-dependent interspecies-weighted momenta, $a_{1}{\hat{P}}_{Y_{1}}+a_{2}{\hat{P}}_{Y_{2}}$ and $b_{1}{\hat{P}}_{X_{1}}+b_{2}{\hat{P}}_{X_{2}}$, and position, $\beta_{1}{\hat{X}}_{1}+\beta_{2}{\hat{X}}_{2}$ and $\alpha_{1}{\hat{Y}}_{1}+\alpha_{2}{\hat{Y}}_{2}$, operators. Alternatively, it can be expressed using the intraspecies momentum and position variances as well as the respective interspecies cross-terms. The contributions of the various intraspecies and interspecies terms to the mixture’s angular-momentum variance are different at the many-body and mean-field levels of theory, and are computed and discussed in the main text. In particular, the momentum and position interspecies cross-terms in (A30) vanish at the mean-field treatment of the (driven) mixture. References
